# Testing the impact of morphological rate heterogeneity on ancestral state reconstruction of five floral traits in angiosperms

**DOI:** 10.1038/s41598-018-27750-1

**Published:** 2018-06-21

**Authors:** Elisabeth Reyes, Sophie Nadot, Maria von Balthazar, Jürg Schönenberger, Hervé Sauquet

**Affiliations:** 1 Ecologie Systématique Evolution, Univ. Paris-Sud, CNRS, AgroParisTech, Université Paris-Saclay, 91400 Orsay, France; 2 Department of Botany and Biodiversity Research, University of Vienna, Rennweg 14, Vienna, A-1030 Austria; 3 National Herbarium of New South Wales (NSW), Royal Botanic Gardens and Domain Trust, Sydney, Australia

## Abstract

Ancestral state reconstruction is an important tool to study morphological evolution and often involves estimating transition rates among character states. However, various factors, including taxonomic scale and sampling density, may impact transition rate estimation and indirectly also the probability of the state at a given node. Here, we test the influence of rate heterogeneity using maximum likelihood methods on five binary perianth characters, optimized on a phylogenetic tree of angiosperms including 1230 species sampled from all families. We compare the states reconstructed by an equal-rate (Mk1) and a two-rate model (Mk2) fitted either with a single set of rates for the whole tree or as a partitioned model, allowing for different rates on five partitions of the tree. We find strong signal for rate heterogeneity among the five subdivisions for all five characters, but little overall impact of the choice of model on reconstructed ancestral states, which indicates that most of our inferred ancestral states are the same whether heterogeneity is accounted for or not.

## Introduction

The evolution of morphological characters can be studied at various scales and by diverse approaches. One approach is to use a phylogenetic tree of the examined species and to reconstruct the ancestral states of a given character set. Maximum likelihood using the continuous-time Markov model^1–4^ is one of the most common methods available to reconstruct ancestral states for discrete morphological characters. It calculates the probability (proportional relative likelihood) of each character state at each node. To do so, instantaneous transition rates among states must first be estimated by finding the set of parameter values that maximizes the overall likelihood of the data, given a phylogenetic tree with branch lengths and a distribution of observed values at the tips^3^. However, the transition rate itself can affect the probabilities of states. On a time-calibrated tree, one of the advantages of maximum likelihood over parsimony (which simply infers the minimum number of changes necessary to explain the distribution of tip states) is that it accounts for the time of a character change. Thus, under low transition rates (or short divergence times between species), the common ancestor of two sister species sharing the same state will have a much higher probability of being in the same state as the two sister species, than of being in any other state. Under higher rates (or longer divergence times between species), the probability of a state change happening between a node and its descendant or tip increases.

Transition rates calculated using maximum likelihood are conditioned by the model chosen. Moreover, the estimated rates may depend on the size of the tree (i.e. number of terminal taxa) as larger trees increase the likelihood for transition rates to be different across the tree^5^. While transition rates may certainly impact reconstructed ancestral states, it remains unknown how strong the impact on ancestral states and, hence, the reconstructed evolutionary history is, as these issues have so far only been very little explored^5,6^.

Angiosperms (flowering plants) comprise approximately 300,000 described species. The large size of the clade entails that two main approaches in the study of character evolution have been applied: an angiosperm-wide approach with a very limited number of representative taxa sampled from each subclade^7,8^ or a focus on individual subclades with more extensive, clade-specific taxon sampling^9–12^. The first approach inevitably conceals the true extent to which some characters change across angiosperms. The second approach, while more likely to capture most, if not all, of the character state changes in the clade of focus, reveals only parts of a much bigger picture. However, detailed present knowledge of angiosperm relationships based on molecular phylogenetic analyses^13^ and the ever increasing record of flower morphology^14^, allow us now to combine both these approaches, and thus, to evaluate the impact of various assumptions and modifications of the model on reconstructed ancestral states.

The flower is the most distinctive attribute of the angiosperms and, despite a well-conserved ground plan with sterile organs in the periphery, the perianth, followed towards the centre by first male (stamens) and then female (carpels) reproductive organs, it is remarkably variable^15^. Features of the perianth such as symmetry, fusion, phyllotaxis, merism, and organ differentiation are important structural characters of the flower that can be identified and compared across all angiosperms. Bilateral symmetry and perianth fusion both contribute to plant-pollinator specialization by restricting flower access to specific animal pollinators. Phyllotaxis, on the other hand, has an important impact on perianth architecture, as some floral innovations (e.g., long corolla tubes) are only possible with whorled but not with spiral phyllotaxis. Pentamery is the most abundant type of perianth merism in angiosperms, for reasons that have yet to be revealed. Perianth organ differentiation results in the specialization of perianth parts for different functions, including the well-known division between protective but inconspicuous sepals and showy but fragile petals. Because of their functional significance, these characters have received much attention in previous works and have been suggested as potential key innovations at the scale of angiosperms^16,17^. Confirming the key innovation status of a given character state (e.g., perianth fusion) requires showing that its origin is correlated with a higher rate of species diversification, which in turn necessitates a clear understanding of where in the tree that state originates. Although floral evolution has been clarified using parsimony among early diverging angiosperms^7,18–20^ and was recently reconstructed on the entire angiosperm phylogeny^14^, we here provide a much more densely sampled dataset to address this question using the maximum likelihood method in addition to parsimony approaches. We further explore the sensitivity of our results to various assumptions for evolutionary models, in particular among-lineage rate constancy.

The first studies of models that took into account the possibility of among-lineage evolutionary rate variation were published in the 1990s^21,22^. Earlier applications of such models on non-molecular data include those of Collar *et al*.^23^ and O’Meara *et al*.^24^ for continuous traits, and Thomas *et al*.^25^ for discrete traits. Beaulieu *et al*.^6^ proposed a model to take hidden transition rate changes into account and applied it to plant habit in Campanulidae. As part of exploring the evolution of traits that enable plants to survive freezing temperatures, Zanne *et al*.^26^ compared a set of transition rate estimates at the level of the angiosperms to those estimated for Magnoliidae, Monocotyledoneae, Superrosidae and Superasteridae. These four clades had noticeably different transition rates from each other and from angiosperms as a whole, as well as different frequencies of the occurrence of character state combinations.

Using parsimony on a 761-species dataset, we previously reconstructed ancestral states for perianth symmetry across angiosperms^27^. In the present study, we expand our dataset to include five floral (perianth) traits recorded in 1230 species, representing 414 of the 416 families (all except Macarthuriaceae and Kewaceae) and all 64 orders recognized by APG IV^28^. We use this new dataset to test the impact of various assumptions of models on estimated transition rates and ancestral states. First, we test the impact of symmetric (Mk1) vs. asymmetric (Mk2) rate models on discrete binary character evolution^5,29–31^. Further, we compare both best-fit models and estimated transition rates among the five distinct characters in order to measure among-character rate heterogeneity^26,30,32–35^. Last, we explore the impact of whole-tree rate constancy by relaxing this assumption and allowing five partitions (ANA + Magnoliidae, Monocotyledoneae, basal eudicots, Superrosidae and Superasteridae, see definition in Methods) of the angiosperm tree to evolve at their own rates^26^. The impact of model change was tested on two sets of selected nodes: one consisting of 15 angiosperm key nodes (Supplementary Tables S2 and S4), and the second of the common ancestors of most (54) of the recognized orders (Supplementary Tables S3 and S5).

## Results

### Rate differences between full tree and partitions

**Figure 1:**
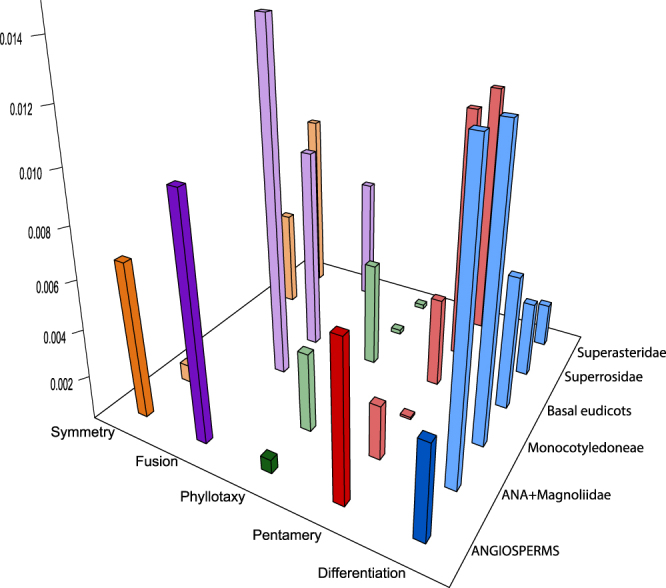
Histogram of the Mk1 transition rates (number of transitions per Ma) for the full tree (dark colours) and five partitions (light colours) for each of the five characters.

We estimated transition rates (q) on the angiosperm-wide tree using the Mk1 and Mk2 models. For each model, one set of estimates corresponds to the entire tree under the same model (referred to as full tree) and another set to different models for the five partitions of the tree (referred to as partitioned tree). The Mk1 transition rates of the five characters, which are shown in Fig. 1 for both the full and the partitioned tree, are used here for comparison.

Some partitions have very high transition rates compared to the full tree. They may be so high that they cause every single node to have the same state probability regardless of the state of the tips sampled (see high transition rate equilibrium in Methods for more details). These rates are not shown on the transition rate histogram (Fig. 1), because they can also be several orders of magnitude higher than rates that are within the range that enables informative ancestral state reconstruction. The [ANA + Magnoliidae] (including Chloranthales, not in partition name to keep designation short) and Monocotyledoneae partitions are the most notable cases of having a higher transition rate than the full tree: both have a transition rate just under 0.013 for perianth differentiation, while the character is found to have a transition rate of 0.0041 when optimized on the full tree. In contrast, some partitions are found to have much lower transition rates than full tree estimates; e.g., merism has a transition rate of 0.0001 in Monocotyledoneae, while that of angiosperms is of 0.0068.

Five combinations are absent from the transition rate histogram shown in Fig. 1. Two concern fusion, once in [ANA + Magnoliidae] and once in Superrosidae, and two concern symmetry, once in Monocotyledoneae and once in basal eudicots. In all of these cases, transition rates are so high that the ancestral states of all nodes examined are equivocal; their estimated transition rates range from 0.064 to 7.174; for comparison, the highest transition rate in our data not causing all nodes to be equivocal is 0.015 (fusion in Monocotyledoneae). In addition, there is no change of perianth phyllotaxis in Monocotyledoneae in our dataset, making the inclusion of their estimated Mk1 rate (q = 1.03 × 10^−10^) in the histogram irrelevant to the test as well.

### Best-fit tree models

**Figure 2:**
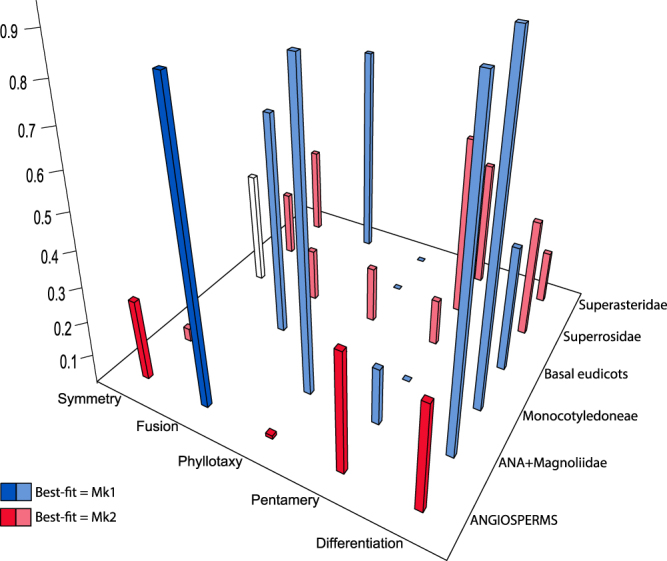
Histogram of Mk2 symmetry ratios (lowest value divided by the highest) for the full tree (dark colours) and partitions (light colours). The scale goes from 0 (unidirectional evolution) to 1 (rates identical, effectively a Mk1 model). Blue bars are best fitted by Mk1 or have no difference in fit between Mk1 and Mk2. Red bars are best fitted by Mk2. The white bar is from a partition in which the transition rate was exceptionally high under Mk1 but not Mk2, making the latter the only one available for comparison.

The evolution of four out of five characters is better fitted by Mk2 than by Mk1 when mapped on the full tree (Fig. 2; Supplementary Table S1), suggesting strong rate asymmetry for most traits. Assuming that all partitions follow the same type of model (i.e., five Mk1 or five Mk2 models), a better fit to the data is consistently obtained when each partition is given its own model instead of using a single model for the entire angiosperm tree (Supplementary Table S1), suggesting strong among-lineage rate heterogeneity in the evolution of these characters. The smallest AIC difference between the full tree and the corresponding combination of five partition models is 10 (for the Mk2 model applied to the characters symmetry and phyllotaxis), which is high enough for the models to be considered very different. In reality, the transition rate differences between partitions can result in best-fitted model differences (Fig. 2; Supplementary Table S1). If the models best fitted to each individual partition are combined for a given character, they will fit the entire angiosperm tree better than five Mk1 models or five Mk2 models. For instance, according to Fig. 2, the best composite model for differentiation would consist of Mk1 models for [ANA + Magnoliidae], Monocotyledoneae and basal eudicots, and Mk2 models for Superrosidae and Superasteridae.

There is a general tendency for full trees and partitions that have a high transition rate symmetry ratio under Mk2 (e.g. the two estimated transition rates are similar to each other) to be best fitted by Mk1 or have neither model fit better than the other, and for those that have a low symmetry ratio (e.g. the transition rates are different from each other) to be best fitted by Mk2. When Mk1 rates are imposed on the tree, fusion, symmetry, merism and differentiation have their lowest AIC at q = 0.01. Phyllotaxis has its lowest AIC at q = 0.001.

### Character optimization results

Parsimony results for our test nodes and the morphological data contributed by each species of our tree are shown in Fig. S1. Overall, most of the nodes tested are not very sensitive to model changes, although we observe more differences at shallow nodes (e.g. those corresponding to crown group nodes of orders or lower taxa; Supplementary Tables S3 and S5) than at deep nodes (e.g., the 15 key clades; Supplementary Tables S2 and S4). Moreover, model sensitivity is greater for perianth differentiation than for the other four characters considered in this study.

#### Full tree and partitions under Mk1 and Mk2

Changes in the most likely state induced by switching from the full tree model to a partition model are more frequent under Mk2 than under Mk1. The partitioned tree is less affected by a change from Mk1 to Mk2 than the full tree. To simplify the interpretation of results, we classify the proportional relative likelihoods of character states in three categories: a probability ≥ 0.90 (default in Mesquite) is considered as strong support and indicated by two asterisks in Table 1 and Supplementary Tables S2 and S3; values between 0.61 and 0.89 are considered as moderate support and indicated by a single asterisk; values ≤ 0.60 are considered as weak support. While the term “equivocal” is commonly used for probabilities that are covered by weak and moderate support, in this study, we use it for state probabilities of exactly 0.5. Table 1 summarizes the results for the crown group node of Caryophyllales, as an example of a node subject to changes in both fusion state and differentiation state depending on the reconstruction model.

**Table 1:** Ancestral state reconstruction for Caryophyllales; most parsimonious character state; most-likely character state of full tree and partitions under Mk1 and Mk2 (full = full-tree model; part = partitioned-tree model).

Caryophyllales	Parsimony	Mk1 (full)	Mk1 (part)	Mk2 (full)	Mk2 (part)
Symmetry	actinomorphic	actinomorphic**	actinomorphic**	actinomorphic**	actinomorphic*
Fusion	fused	free*	fused*	free*	fused*
Phyllotaxis	whorled	whorled**	whorled**	whorled**	whorled**
Merism	pentamerous	pentamerous**	pentamerous**	pentamerous**	pentamerous*
Differentiation	undifferentiated	differentiated**	differentiated**	differentiated*	differentiated*

Asterisks in the maximum likelihood columns: none = weak support (0.51–0.60), one = moderate support (0.61–0.89), two = strong support (0.90–1).

The results for the full tree and the five partitions, under both Mk1 and Mk2, for the 15 key nodes are given in Supplementary Tables S2. Table 2 shows the number of nodes that change for each pair of tests under Mk1 versus Mk2 and the full versus the partitioned tree. The results for the full tree and the five partitions, under both Mk1 and Mk2, for the 54 tested order nodes, were assembled in Supplementary Tables S3. Table 3 shows the number of nodes that change for each pair of tests under Mk1 vs Mk2 and the full versus the partitioned tree. It should be noted that in many of the above cases, the order nodes in which fusion and differentiation states change between models are directly derived from key nodes that exhibit a similar behaviour. For instance, all key nodes and the vast majority of order nodes that change differentiation states between models belong to the three basal-most partitions. This indicates that sensitivity of most-likely states to model changes tends to affect clusters of closely related nodes.

**Table 2:** Number of changes in most-likely estimated ancestral state between models and tree types across 15 key nodes.

15 nodes (full) 13 nodes (part)	Mk1 (full) ≠ (part)	Mk2 (full) ≠ (part)	(full) Mk1 ≠ Mk2	(part) Mk1 ≠ Mk2
Symmetry	0	0	1	0
Fusion	1	1	0	0
Phyllotaxis	0	4	5	0
Merism	0	0	0	0
Differentiation	1	4	6	2

The method used to partition the tree reduced the reconstructable key node number from 15 to 13. The nodes that were lost (Mesangiospermae, Pentapetalae) have their names written in italics in Fig. 4. Each column compares the results of two of the model and tree type combinations and reports the number of nodes that change their most-likely state in each case. Mk1 = one rate ML model; Mk2 = two-rate ML model; full = full tree; part = partitioned tree.

**Table 3:** Number of changes in most-likely estimated ancestral state between models and tree types across 54 botanical orders.

54 nodes	Mk1 (full) ≠ (part)	Mk2 (full) ≠ (part)	(full) Mk1 ≠ Mk2	(part) Mk1 ≠ Mk2
Symmetry	0	2	3	2
Fusion	1	1	0	0
Phyllotaxis	1	8	7	0
Merism	0	0	0	0
Differentiation	2	9	16	2

Each column compares the results of two of the model and tree type combinations and reports the number of nodes that change most-likely state in each case. Mk1 = one rate ML model; Mk2 = two-rate ML model; full = full tree; part = partitioned tree.

Most states reconstructed by maximum likelihood are identical to those inferred by parsimony. There are a few cases of conflict between parsimony and at least one of the likelihood reconstructions, both at the key node level and the order node level. For instance, the ancestral state for Superrosidae and Rosidae is “fused” according to parsimony, but “free” according to the Mk1 and Mk2 full tree models. The number of such cases is five or fewer (depending on the character) at key node level and less than ten at order node level.

#### Constrained Mk1 rates

All nodes for all characters, including those reported in the present study but also any shallow nodes, reach their high transition rate equilibrium (0.5 probability for both states) at q = 0.1 changes per Ma, suggesting that such a rate of evolution is too high to make any inference on ancestral states in angiosperms. Fusion and differentiation are found to be more sensitive to these arbitrary rate changes than symmetry, phyllotaxis and merism. Table 4 summarizes the results for Caryophyllales, as an example of a node subject to change depending on the transition rate. The results for all test nodes are assembled in Supplementary Tables S4 (key nodes) and Supplementary Tables S5 (order nodes).

**Table 4:** Most-likely ancestral state and state probability under constrained rates for Caryophyllales.

Caryophyllales	Parsimony	q = 0.0001	q = 0.0010	q = 0.0100	q = 0.1000
Symmetry	actinomorphic	actinomorphic (1)	actinomorphic (1)	actinomorphic (1)	equivocal (hr)
Fusion	fused	fused (1)	fused (0.93)	free (0.63)	equivocal (hr)
Phyllotaxis	whorled	whorled (1)	whorled (1)	whorled (1)	equivocal (hr)
Merism	pentamerous	pentamerous (1)	pentamerous (1)	pentamerous (1)	equivocal (hr)
Differentiation	differentiated	differentiated (1)	differentiated (1)	differentiated (0.90)	equivocal (hr)

q = imposed Mk1 transition rate. The indicated state is the most-likely, followed by its relative probability. (hr) = high rate equilibrium.

**Figure 3:**
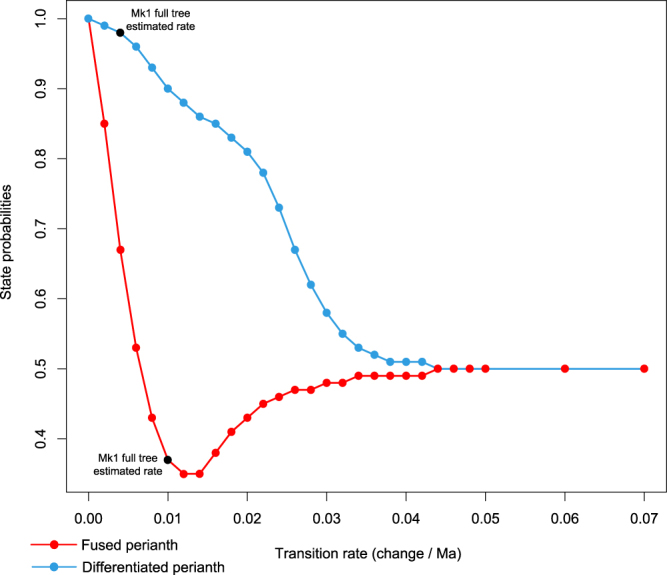
Caryophyllales state probabilities in function of fixed Mk1 transition rate. Black dots correspond to the points closest to the rate and probability obtained by estimation. Each of these states is also the one inferred by parsimony reconstruction. In both cases, the probability of the other state has the exact opposite evolution.

Two main types of results were observed. Figure 3 shows, for the Caryophyllales node, the evolution of the state probability of a fused and a differentiated perianth as a function of the transition rate with denser sampling of transition rates than all other nodes. On the one hand, the probability of a fused perianth shows the general pattern of a node that can change most-probable states. On the other hand, the probability of a differentiated perianth presents the pattern of a node that does not change state, with a gradual probability decrease that eventually reaches the high transition rate equilibrium (0.5) and does not leave it. The most noticeable difference between the two curves is the dip formed by the fusion curve as the result of going under 0.5, then later converging towards it.

#### Parsimony reconstructions

The number of origins and reversals was counted on parsimony instead of maximum likelihood reconstructions. The latter posed problems when data were contradictory or when the basal-most taxon of a clade lacked data for the character (the data in our tables is for the deepest internal node in such cases). The number of transitions in angiosperms as a whole, along with the number range for origins and reversals, is shown in Table 5. A NEXUS file containing the tree and both the rescored and original data matrices is provided as Supplementary Data S1. A per-partition breakdown of the origins and reversals is shown in Supplementary Table S6. There are at least 148 origins of bilateral symmetry, 71 of perianth fusion, 23 of spiral phyllotaxis, 25 of a pentamerous perianth, and 44 of a differentiated perianth.

**Table 5:** Number of transitions with origin and reversal number range.

	State changes	Origins (min-max)	Reversals (min-max)
Symmetry	217	148–182	35–69
Fusion	212	71–130	82–141
Phyllotaxis	31	23–28	3–8
Merism	226	25–59	167–201
Differentiation	161	44–76	85–117

### Sensitivity of results to the dataset and tree used

In the main results presented here, polymorphic data were rescored according to the presumed derived state (see Methods). We explored the sensitivity of our results to this assumption by conducting all analyses again on the original dataset and found very few differences (see Supplementary Note 1 and Tables 1–6). In addition, we conducted all analyses again using a different ultrametric tree of 1215 taxa extracted from a recently published mega-tree of 353,185 taxa^36^. Likewise, we found that nearly all of our results are robust to the method used to build an ultrametric tree matching our trait dataset (see Supplementary Note 2 and Tables S1–S6).

## Discussion

We show that reconstructed ancestral states are robust to the different models we tested. Similar robustness of ancestral state inference to varying rates and models had previously been reported for parametric biogeographic methods using the DEC model^37–39^. We further show that altering transition rates, while all other parameters remain unchanged, can impact the most likely state of a node. In addition, our analyses indicate that the probability of a given character state as a function of the Mk1 transition rate can follow two different types of curves, which always converge towards an equilibrium of 0.5 at high transition rates. This means that as transition rates increase, it becomes less and less likely that character states are reconstructed with strong support. To some extent, our estimated rates are probably influenced by the sampling strategy used in this study. However, a comparison with previous work suggests that global rate patterns remain reliable here, with the exception of extremely high rates that are clearly unrealistic, and which we interpret as an artefact (see Supplementary Discussion).

The model made of five angiosperm partitions is always better fitted than the model that made rates uniform across the full tree. These results are consistent with those of recent studies that found a significant signal for morphological rate heterogeneity in large clades^5,6,26^. This suggests that adequate modelling of morphological rate variation is an important direction of future, macroevolutionary research^32,40^. However, our results indicate that, at least for the five traits considered here in angiosperms and the specific partition scheme we used, failing to account for morphological rate variation had only a minor impact on reconstructed ancestral states at key nodes of the phylogeny. This may be in part due to the fact that the transition rates of the characters do not necessarily vary according to the partition scheme that we used, and results may be different in partition schemes that reflect the actual transition rate shifts of each character better.

There are four character and tree partition combinations for which Mk1 and Mk2 are equally well fitted to the data. This is the case despite of a low symmetry ratio between the Mk2 transition rates (e.g. the rates are extremely different from each other), which should make Mk2 a significantly better fit than Mk1. These combinations correspond to characters for which, in that specific partition of the tree, one of the states has very few origins with no reversals, yet the estimated reversal rate is five to 143 times higher than the very low origin rate. We suspect that these high reversal rates are an artefact of ML optimization with the Mk2 model when reversals are not observed in the data. A similar phenomenon was observed in earlier studies^14,30,41^ and appears to emerge when a particular state is rare and when, at the same time, asymmetric rates are allowed such as in the Mk2 model. Clearly, more work is needed on identifying, characterizing, and preventing such best model identification problems.

In general, when mapping character evolution on the full tree (i.e., unpartitioned analyses), greater differences between the Mk1 and Mk2 models are observed than when allowing among-lineage rate heterogeneity (i.e., partitioned tree analyses). For phyllotaxis, one of the characters strongly subjected to this phenomenon, we suspect that the results from the [ANA + Magnoliidae] partition may be more accurate than the results of the Mk2 full tree. A possible reason may be that basal angiosperms are highly variable for phyllotaxis compared to the rest of angiosperms^14^, and a model fitted to the partition takes the local heterogeneity better into account. It may imply that the model that fits the phyllotaxis full tree best, Mk2, is actually less accurate than the less well supported Mk1^42^. Simulations may be required to confirm this observation. King and Lee^5^ used simulated trees and tested if the model that was used to generate the tree would be favoured in a model fit test. In doing so, they discovered that the highest likelihood was occasionally found for inaccurate models. In our case, we suspect that the advantage of the Mk2 model over the Mk1 model for the full phyllotaxis tree may be an artefact due to the artificially high reversal rate of this character, which in turn caused the Mk2 model to be favoured by AIC despite being inaccurate. Another phenomenon observed by King and Lee^5^, which could also be affecting our full tree reconstructions, was the Mk2 model compensating for poorly modelled among-lineage rate heterogeneity by inferring many reversals in the clades changing rapidly for any given character.

We also compared the ratio of origins and reversals of each character under parsimony (Table 5) with the forward-reverse ratio of transition rates estimated with the Mk2 model for the full tree (Supplementary Table 1). We found no relationship between these two ratios, which were in most cases substantially different. For instance, the parsimony ratio for perianth symmetry ranged from 2.1 to 5.2 (indicating that gains of zygomorphy are more frequent than reversals), whereas the Mk2 transition rate ratio was 0.2 (indicating that origins occur at a slower rate than reversals). This apparent inconsistency might be interpreted as additional evidence that either parsimony or the Mk2 model applied to the full tree incorrectly accounts for the true process underlying the evolution of this trait. However, we warrant caution with this comparison because the parsimony analyses conducted in this study assumed equal weight of forward and reverse changes and are thus more comparable to the Mk1 model (for which the transition rate ratio is always 1 by definition).

We found two patterns for the evolution of state probability in function of transition rate value. In the first pattern, state probabilities that are different from each other at low rates gradually converge until they reach the high rate equilibrium, in which they are both at 0.5 (e.g., perianth fusion, Fig. 3). In the second pattern, the state that is the least probable of the two at low transition rates becomes more and more probable as transition rates increase, then temporarily becomes more probable than the other state, and then decreases in probability as it converges towards the high rate equilibrium (e.g., perianth differentiation, Fig. 3). In the latter case, the state that the node most likely has under lower transition rates is consistently identical to the one it has under parsimony, while the other state is the most probable under higher rates. This is consistent with the observation by previous authors^43,44^ that use of parsimony is only suitable when rates of character state change are low.

Our analyses also reveal a pattern of many origins and reversals in all our characters (217 total changes for symmetry, 212 for fusion, 31 for phyllotaxis, 226 for merism and 161 for differentiation; Supplementary Data S1, Supplementary Discussion, Supplementary Table 6). We note that parsimony likely underestimates these numbers compared to maximum likelihood^44^. The lowest number of state changes is present in phyllotaxis where most changes occur in two non-Pentapetalae groups, whereas Pentapetalae are fairly homogenous, contributing less than a third of the total number of changes. Merism has the highest number of state changes, due to the high number of changes from ancestral pentamery to non-pentamery in the large Pentapetalae clade. Overall, we find more origins of derived states than in any previous reconstruction for the same characters (Supplementary Discussion). We note that our sampling approach may be underestimating the number of changes (due to the limited phylogenetic resolution of some families in our tree) or overestimating the rates (Supplementary Discussion), but we found very similar results using a different, entirely resolved tree (Supplementary Note 2, Table S6).

### Conclusion and perspectives

We found that most ancestral states for deep as well as shallow nodes of the angiosperm tree were largely unaffected by changes in models (number of transition rates in model, value of transition rates, tree partitioning). However, we found a strong signal for among-lineage rate heterogeneity in perianth evolution, and we observed that accounting for this heterogeneity may influence some reconstructed ancestral states. On the one hand, this may mean that most ancestral states reconstructed in this and other model-based studies^14,45^ are similar irrespective of whether rate heterogeneity is taken into account or not. On the other hand, we do not know whether the number of model-sensitive nodes in our dataset is normal, unusually low, or even unusually high, which makes predictions for other data than our own uncertain. Therefore, it will be necessary to conduct additional work on other clades, traits and rate heterogeneity models (including new approaches for automatic identification of rate shifts without a priori tree partitioning) before it can be asserted whether the results of this study are general. We further observed that, for the equal-rate (Mk1) model, low rates lead to results most similar to the parsimony optimization, while very high rates lead to complete uncertainty, consistent with the predictions of the Markov model. These factors will need to be taken into account in future studies focusing on ancestral state reconstruction.

## Methods

We recorded five perianth characters (see below) for a large sample of angiosperm species using an exemplar approach. We included at least one species from each currently recognized family (except Kewaceae and Macarthuriaceae; see details below). Families lacking a perianth, such as Piperaceae and Ceratophyllaceae, were treated as missing data, but maintained in our sample in order to present a complete overview of floral trait evolution mapped on phylogenetic trees^27^. All data were scored in the PROTEUS database^46^ (the complete list of 5924 explicit records and linked references is provided as Supplementary Data S2; for a general explanation of the database and scoring philosophy, see Supplementary Information of Sauquet *et al*.^14^). APG IV^28^ was followed for order and family delimitations. Higher-level clades above the rank of order follow the definitions given by Cantino *et al*.^47^ and Soltis *et al*.^48^.

### Choice of characters

To measure the impact of transition rate heterogeneity on ancestral state reconstruction, we selected five important perianth characters, chosen because of their broad applicability across angiosperms, their biological significance, and their variability in most larger clades.

#### Symmetry

Perianth symmetry has two main states: actinomorphy (radial symmetry, polysymmetry), which is ancestral in angiosperms^14,16,27^, and zygomorphy (bilateral symmetry, monosymmetry). Zygomorphy is found in all major clades of angiosperms^27,49^. Rare cases of asymmetry and disymmetry are treated here as missing data, as in Reyes *et al*.^27^.

#### Fusion

All floral organs are considered to be ancestrally free from each other^14,16^. Sympetaly, the fusion of petals, appeared repeatedly across angiosperms and is considered to be a key innovation in Asteridae^16,50^. Here, we considered within-whorl fusion for the perianth as a whole, scored on a continuous scale of 0 to 1, and then transformed in PROTEUS as a binary character using a threshold of 0.05. In practice, regardless of the extent of inter-whorl fusion, free whorls were coded as 0, fused whorls were coded as 1, and the fusion state of the whole perianth could be 0 (all whorls free), 1 (all whorls fused) or 0–1 (polymorphic).

#### Phyllotaxis

Phyllotaxis of perianth organs exhibits two main states: whorled and spiral. Spiral perianth phyllotaxis is much more frequent in basal angiosperms (the ANA grade and Magnoliidae) than in the rest of the group and has been hypothesized to represent an obstacle to various forms of floral elaboration via fusion of organs that evolved in many whorled flowers^51,52^. Some characteristics of spiral flowers have led many authors to consider that it is the ancestral state in angiosperms^51,53–55^. However, most recent ancestral state reconstructions using parsimony find the two states to be equivocal at the root of angiosperms^7,18,19,52,55,56^ and recent work by Sauquet *et al*.^14,57^ suggest it might be whorled.

#### Merism

Flowers with a pentamerous perianth are characteristic of one of the largest clades in angiosperms, Pentapetalae^47^, representing 75% of all angiosperms. This makes pentamery the most frequent form of perianth merism found across angiosperms^58^. Although pentamery is present in other clades, such as in Ranunculaceae (basal eudicots) and Siparunaceae in Magnoliidae^16,59,60^, the reason why it is so predominant in Pentapetalae remains unknown. Sauquet *et al*.^14^ inferred the ancestral perianth to be trimerous and clearly demonstrated that pentamery is derived in angiosperms.

#### Differentiation

A perianth made entirely of parts identical to each other, i.e., undifferentiated, is the most common type in basal angiosperms. By contrast, most flowers of angiosperms are characterized by a differentiated perianth, comprising at least two whorls of parts different from each other in colour, size, shape, and anatomy. According to several studies, the ancestral state in angiosperms has been considered as undifferentiated^7,8,14,19,20,55^, or equivocal^18,56^.

Following initial recording in PROTEUS, the matrix of five characters and 1230 species for this study was exported as a NEXUS file from the database and transformed in Mesquite 3.04^61^ in such a way that all polymorphic cells were converted to the presumed derived state (rescored data; Supplementary Data S1), for the purposes of the present study. In the case of phyllotaxis, the derived state was assumed to be spiral, as suggested by Bayesian analyses^14^. The transformation affected character states inferred for 25 species (symmetry), 134 species (fusion), seven species (phyllotaxis), 100 species (merism) and eight species (differentiation), respectively. This was done because most algorithms treat polymorphic terminal taxa as uncertainties rather than entities possessing both states (e.g., due to infra-specific polymorphism or sexual dimorphism), hence causing a possible under-estimation of the number of origins of the derived states. However, we also acknowledge that terminal taxon rescoring comes with the assumption that all species presenting the derived state are in the process of fixing this state, which may not always be true, and hence causes a problem antithetic to that of *not* rescoring the states, which is over-estimation of origins. All analyses were then conducted again with the original, untransformed matrix (see Supplementary Note 1 and Tables S1–S6).

### Character coding and taxon sampling

Family-level descriptions were used to identify families presenting variation in the studied characters. A total of 661 sources were then used to score the perianth traits of particular species, including a large number of scientific articles, *The Families of Flowering Plants*^62^, and the online versions of the *Flora of North America*, *Flora of China* and *Flora of Pakistan*; the latter four sources were also those used for family-level descriptions. Drawings linked to taxonomic descriptions and photos from *Encyclopedia of Life* (ww.eol.org) that had been marked as trusted were used to determine the state of perianth symmetry, merism, differentiation, and fusion when it was not mentioned in the descriptions themselves; photographs were considered unreliable for determining phyllotaxis.

Families with variation in none of the five characters were represented by a single species. For families that were polymorphic for any of the characters, we included as many species as necessary to represent the variation in character states (incl. at least one species in each state), including multiple transitions and/or reversals according to current phylogenetic knowledge of the family when available, and accounting for possible multiple changes in non-resolved families through the inclusion of at least two non-congeneric species in each state when possible, as in our previous study of perianth symmetry^27^. This method resulted in a taxon sample of 1230 species. We acknowledge that this sampling strategy may have resulted in a bias towards overall higher rate estimates and lower rate heterogeneity (see Supplementary Discussion).

### Phylogenetic tree

The backbone tree used here is based on the consensus tree from the Angiosperm Phylogeny Website^13^ and the one from Soltis *et al*.^48^, with the internal phylogenetic structure of families resolved according to the same studies as in Reyes *et al*.^27^ (see Appendix 1 of that article). We used an alternative method to approximate branching times for this study, since many of the species sampled do not have sufficient data in public DNA sequence databases (e.g., GenBank) and it was beyond the scope of this study to generate new sequence data for these species. We employed the bladj function of phylocom^63^ to transform the consensus tree topology into an ultrametric tree, via constraining 399 nodes defining families and deeper clades (Supplementary Fig. S2) to fit the ages of Magallón *et al*.^64^. This approach is not expected to produce accurate divergence times, but may be sufficient for the main purposes of this study. To explore the sensitivity of our results to this question, we also conducted most analyses again using a pruned version of a recently published mega-tree containing 353,185 species of seed plants^36^ (see Supplementary Methods 2 and Note 2).

### Ancestral state reconstruction and transition rate estimation

**Figure 4:**
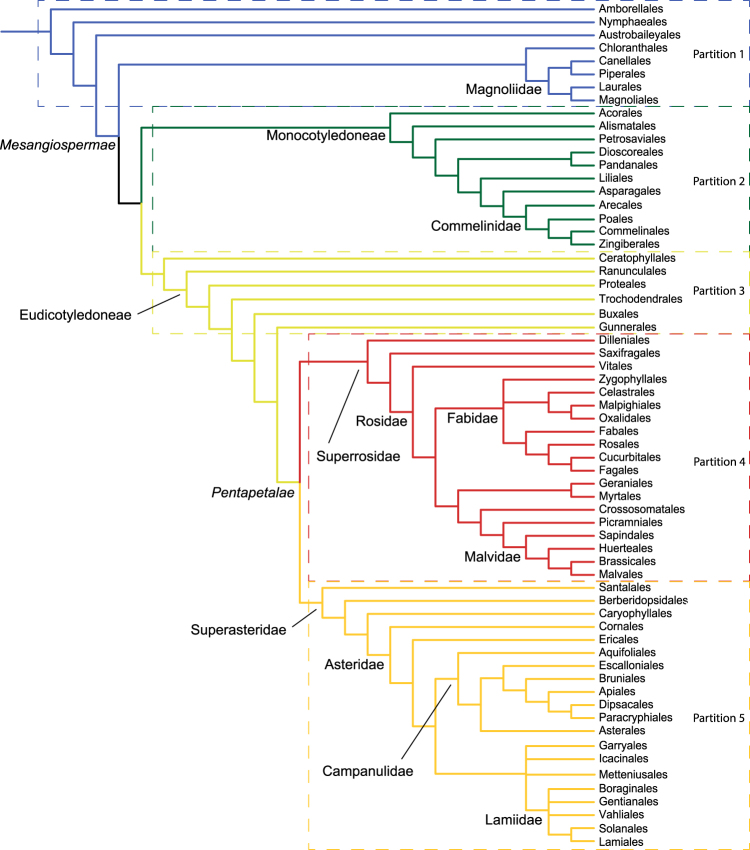
Order-level phylogeny of angiosperms. The members of each partition/subdivision are contained in rectangles. The key nodes in italics are those not reconstructed in the partitioned tree.

Ancestral state reconstruction was performed using parsimony and maximum likelihood in Mesquite 3.04^61^. We used two models for maximum likelihood character optimization: an equal-rate model (Mk1; i.e., single rate of transition for both forward and backward change) and a two-rate model (Mk2; i.e., a model allowing asymmetric rates of forward and backward change). To test and account for among-lineage rate heterogeneity, we used a similar approach as Zanne *et al*.^26^ and subdivided the tree into five partitions consisting of two grades and three large clades, each with its own model and transition rates (Fig. 4; Supplementary Methods). The first partition comprises the ANA grade (Amborellales, Nymphaeales, Austrobaileyales), Chloranthales, and Magnoliidae, the second corresponds to Monocotyledoneae, the third includes Ceratophyllales and basal eudicots (Ranunculales, Proteales, Trochodendrales, Buxales and Gunnerales), the fourth equals Superrosidae, and the fifth Superasteridae (incl. Dilleniales; Soltis *et al*.^48^). The rationale for including two grades in our tree partitioning scheme is that members of these grades may have inherited ancestral transition rates, while the large clades nested in them may have shifted to distinct evolutionary regimes characterized by different transition rates. We acknowledge that this a priori partition scheme is only one of a very large number of possible ways to divide the tree and it would be desirable instead to detect shifts automatically (e.g., King & Lee^5^), an approach we reserve for future work. However, the partitioning scheme used here is in fact meaningful from a floral morphology standpoint, as observed by botanists for a long time^65^ and recently confirmed by the angiosperm-wide analyses of Sauquet *et al*.^14^. For instance, “basal angiosperms” (our ANA + Magnoliidae + Chloranthaceae grade) have long been recognized for their highly diverse floral structures (but usually with many parts, actinomorphic, and without fusion), whereas monocots are particularly homogeneous in basic floral structure (but with much variation in other traits such as symmetry and fusion).

In practice, we pruned the tree to isolate the taxa from a given partition and performed ancestral state reconstruction on the resulting subtree, effectively making each of the partition models independent from each other (as in the “censored” approach of O’Meara *et al*.^24^). With such conditions, the likelihood of a tree-wide model in which each partition has its own rate is proportional to the product of the likelihoods of the model fitting each of the isolated partitions for a given character^24,26^. The Akaike Information Criterion (AIC) was then used to compare the relative fit of the data of the partitioned model to those of the default unpartitioned model.

We also tested the impact of four arbitrary transition rate values on the inferred character states and their probabilities under the Mk1 model on the full tree. Values for each node were recorded fixing transition rates (q) at 0.0001, 0.001, 0.01, and 0.1. An expectation of the model is that a binary character under a Mk1 model always reaches the equilibrium frequency, 0.5, for the probability of both states at any node when transition rates are very high^27,66^; we refer to this phenomenon as the high transition rate equilibrium. This equilibrium is possible for all our test nodes under the higher test rates, whether or not the value of the transition rate affects which state is the most probable. This causes nodes sensitive to arbitrary transition rate change to be detectable *a priori* only via an observed change of most likely state depending on the transition rate. Most-probable states only seen decreasing in probability were considered to be simply converging towards the high transition rate equilibrium.

We also tested for transition rate asymmetry by fitting the Mk1 and the Mk2 models to each trait, both for angiosperms as a whole and for each partition of the tree. The relative fits of the two models were evaluated using the AIC. Further, to compare the relative magnitudes of transition rate asymmetry in Mk2 implementations, we here report on the transition rate symmetry ratio, calculated by dividing the lowest rate by the highest, creating a scale from 0 (unidirectional evolution) to 1 (both rates almost identical, virtually the Mk1 model), and reflecting the similarity between the forward and backward rates of Mk2. We also used the AIC to test which arbitrary rate best fitted the evolution of each character.

Finally, we recorded the number of derived state origins using Fitch parsimony (assuming the same number of steps for gains and reversals). The minimum number of origins was found by treating all equivocal branches as being in the derived state, while the maximum number was found via treating the equivocal branches as being in the ancestral state, as in Reyes *et al*.^27^.

### Data availability

The data are available in the form of Supplementary Data 1, Data 2, and Data 3.

## Electronic supplementary material

Supplementary text and figures

Supplementary Dataset 2

Supplementary Datasets 1 and 3
