# Supplementary material for: Testing the impact of morphological rate heterogeneity on ancestral state reconstruction of five floral traits in angiosperms
Source: Sci Rep. 2018 Jun 21;8:9473. doi: 10.1038/s41598-018-27750-1 (PMC6013437; doi:10.1038/s41598-018-27750-1)
Supplement: Supplementary file 1 — Supplementary text and figures [file 41598_2018_27750_MOESM1_ESM.pdf]

Testing the impact of morphological rate heterogeneity on ancestral state reconstruction of five floral traits in angiosperms

Supplementary Note 1: Results from the original data

Elisabeth Reyes, Sophie Nadot, Maria von Balthazar, Jürg Schönenberger, Hervé Sauquet

Supplementary Note 1: Results from the original data

Original and rescored data. When comparing the original and rescored versions of the Mk1 and Mk2 full and partitioned trees, differences between the two are rare (Tables SN1 and SN2). Differences are most frequently found for fusion and pentamery. The nodes that display differences tend to be ancestors of clades in which there are several rescored species. The Mk2 partitioned tree has the most frequent difference between the original data and the rescored data.

Table SN1: Number of changes in inferred most likely ancestral state between models and tree types across 15 key nodes. Each column compares the results of two of the model and tree type combinations and reports the number of nodes that change most likely state between rescored and original data in each case. Mk1= one rate maximum likelihood model; Mk2= two-rate maximum likelihood model; full= full tree; part= partitioned tree; rescor= rescored data used in article; orig= data as it was entered in PROTEUS.

15 nodes (full)<br>13 nodes (part)	**Mk1 (full)**<br>(rescor) ≠ (orig)	**Mk2 (full)**<br>(rescor) ≠ (orig)	**Mk1 (part)**<br>(rescor) ≠ (orig)	**Mk2 (part)**<br>(rescor) ≠ (orig)
symmetry	0	0	0	0
fusion	1	1	0	4
phyllotaxis	0	0	0	0
pentamery	0	0	0	4
differentiation	0	0	2	0

Table SN2: Number of changes in inferred most likely ancestral state between models and tree types across 54 botanical orders. Each column compares the results of two of the model and tree type combinations and reports the number of nodes that change most likely state between rescored and original data in each case. Mk1= one rate maximum likelihood model; Mk2= two-rate maximum likelihood model; full= full tree; part= partitioned tree; rescor= rescored data used in article; orig= data as it was entered in PROTEUS.

54 nodes	**Mk1 (full)**<br>(rescor) ≠ (orig)	**Mk2 (full)**<br>(rescor) ≠ (orig)	**Mk1 (part)**<br>(rescor) ≠ (orig)	**Mk2 (part)**<br>(rescor) ≠ (orig)
symmetry	1	0	0	0
fusion	3	2	2	8
phyllotaxis	0	1	0	0
pentamery	2	1	0	7
differentiation	1	0	3	1

Full tree and partitions under Mk1 and Mk2. The states of all the tested nodes under Mk1 and Mk2 models data can be found in Supplementary Tables S2 (15 key nodes) and S4 (54 order nodes). When compared with the rescored data, fusion and pentamery are both more prone to give different results between reconstructions (Tables SN3 and SN4). These characters are the ones with the largest number of species that have states affected by rescoring. The nodes in which there are differences between the two types of data (original as it was entered in PROTEUS and rescored for the purposes of the main article) tend to be ancestors of clades in which there are several rescored species. As in the rescored results, the most frequently found difference in inferred state are between the Mk2 full and partitioned trees and the Mk1 and Mk2 full trees. One of the differences compared to the rescored data is a more frequent occurrence of differences between the Mk1 and Mk2 partitioned trees.

Table SN3: Number of changes in inferred most likely ancestral state between models and tree types across 15 key nodes. Each column compares the results of two of the model and tree type combinations and reports the number of nodes in which the most likely state changes in each case. Mk1= one rate maximum likelihood model; Mk2= two-rate maximum likelihood model; full= full tree; part= partitioned tree.

15 nodes (full)<br>13 nodes (part)	**Mk1**<br>(full) ≠ (part)	**Mk2**<br>(full) ≠ (part)	**(full)**<br>Mk1 ≠ Mk2	**(part)**<br>Mk1 ≠ Mk2
symmetry	0	0	1	0
fusion	2	2	0	4
phyllotaxis	0	4	5	0
pentamery	0	4	0	4
differentiation	3	2	6	0

Table SN4: Number of changes in inferred most likely ancestral state between models and tree types across 54 botanical orders. Each column compares the results of two of the model and tree type combinations and reports the number of nodes that change most likely state in each case. Mk1= one rate maximum likelihood model; Mk2= two-rate maximum likelihood model; full= full tree; part= partitioned tree.

54 nodes	**Mk1**<br>(full) ≠ (part)	**Mk2**<br>(full) ≠ (part)	**(full)**<br>Mk1 ≠ Mk2	**(part)**<br>Mk1 ≠ Mk2
symmetry	0	1	4	1
fusion	1	6	1	7
phyllotaxis	1	7	6	0
pentamery	2	8	2	8
differentiation	6	8	15	1

Most inferred states are identical to those inferred by parsimony or can be both states according to parsimony. The number of nodes for which the state found by at least one of the maximum likelihood

models does not match with the parsimony state is a little higher with the original data than with the rescored data in both sets of nodes, but remains in the same order of magnitude.

Constrained Mk1 rates. The states of all nodes under constrained rates can be found in Supplementary Tables S3 (15 key nodes) and S4 (54 order nodes). All but one of the fusion nodes that have their state dependent on the transition rate with the rescored data show what seems to be a simple probability decrease pattern with the original data. At the order level, pentamery gains a state-changing node it did not have with the rescored data. The state-changing node numbers for symmetry, phyllotaxis and differentiation remain the same and are the same nodes as in the rescored data.

Parsimony reconstructions. The number of transitions in angiosperms as a whole, along with the number range for origins of derived states and reversals to ancestral states, is shown in Table SN5. Origins are at least 134 for zygomorphy, 81 for fusion, 13 for spiral phyllotaxis, 33 for pentamery and 44 for differentiation. A per-partition breakdown of the origins and reversals is shown in Supplementary Tables S6. The number of state changes is always lower than with rescored data. The phenomenon affects fusion the most, but actually increases the minimum number of origins and reduces minimum number of reversals. The same phenomenon happens for pentamery. Symmetry and phyllotaxis show a decrease in the number of origins along with the decrease in the number of steps.

Table SN5: Number of transitions with origin and reversal number range.

	State changes	Origins (min-max)	Reversals (min-max)
symmetry	191	134-160	31-57
fusion	154	81-119	35-73
phyllotaxis	29	21-29	0-8
pentamery	199	33-96	103-166
differentiation	159	44-76	83-115

Testing the impact of morphological rate heterogeneity on ancestral state reconstruction of five floral traits in angiosperms

Supplementary Note 2: Results for the rescored data mapped on the pruned Smith & Brown tree

Elisabeth Reyes, Sophie Nadot, Maria von Balthazar, Jürg Schönenberger, Hervé Sauquet

<u>Supplementary Note 2: Results for the rescored data mapped on the pruned Smith & Brown tree</u>

Original tree versus pruned Smith & Brown tree. Few nodes change state between the two trees (almost none at key node level, less than five for each character at order level; Tables SN6 and SN7), and most of the states that change are moderately to weakly supported in both trees. We note that the reconstruction of fusion on the partitioned trees had a particularly low number of nodes to compare to each other; three partitions were subject to the high rate equilibrium in at least one of the trees, enabling only the comparison of node states in the basal eudicot and Superasteridae partitions.

Table SN6: Number of changes in inferred most likely ancestral state between models and tree types across 15 key nodes. Each column compares the results of two of the model and tree type combinations and reports the number of nodes that change most likely state between rescored and original data in each case. Mk1= one rate maximum likelihood model; Mk2= two-rate maximum likelihood model; full= full tree; part= partitioned tree; ot= original tree used in article; sb= Smith & Brown tree.

15 nodes (full)<br>13 nodes (part)	**Mk1 (full)**<br>(ot) ≠ (sb)	**Mk2 (full)**<br>(ot) ≠ (sb)	**Mk1 (part)**<br>(ot) ≠ (sb)	**Mk2 (part)**<br>(ot) ≠ (sb)
symmetry	0	1	0	0
fusion	0	0	0	1
phyllotaxis	0	0	0	0
pentamery	0	0	0	0
differentiation	0	0	0	0

Table SN7: Number of changes in inferred most likely ancestral state between models and tree types across 54 botanical orders. Each column compares the results of two of the model and tree type combinations and reports the number of nodes that change most likely state between rescored and original data in each case. Mk1= one rate maximum likelihood model; Mk2= two-rate maximum likelihood model; full= full tree; part= partitioned tree; ot= original tree used in article; sb= Smith & Brown tree.

54 nodes	**Mk1 (full)**<br>(ot) ≠ (sb)	**Mk2 (full)**<br>(ot) ≠ (sb)	**Mk1 (part)**<br>(ot) ≠ (sb)	**Mk2 (part)**<br>(ot) ≠ (sb)
symmetry	0	4	0	1
fusion	0	0	2	8
phyllotaxis	0	0	0	1
pentamery	1	0	0	0
differentiation	1	1	0	4

Full tree and partitions under Mk1 and Mk2. The states of all the tested nodes under Mk1 and Mk2 models can be found in Supplementary Tables S2 (15 key nodes) and S4 (54 order nodes). Compared to the five-character tree, the pruned Smith & Brown tree has an equal or slightly lower number of nodes that change states between models (Tables SN8 and SN9). The only character (fusion) showing an increase has several of its nodes that change between models in Superrosidae, a large partition that was subject to the high rate equilibrium in the original tree (thus preventing state comparison between likelihood models), but not in the Smith & Brown one. There are nodes for which at least one of the likelihood models infers a state different from parsimony, but they remain fewer than five for each character among 15 key nodes, and fewer than ten among the 54 botanical orders. The most frequent differences are between the Mk1 and Mk2 full trees (Tables SN8, SN9).

Table SN8: Number of changes in inferred most likely ancestral state between models and tree types across 15 key nodes. Each column compares the results of two of the model and tree type combinations and reports the number of nodes in which the most likely state changes in each case. Mk1= one rate maximum likelihood model; Mk2= two-rate maximum likelihood model; full= full tree; part= partitioned tree.

15 nodes (full)<br>13 nodes (part)	**Mk1**<br>(full) ≠ (part)	**Mk2**<br>(full) ≠ (part)	**(full)**<br>Mk1 ≠ Mk2	**(part)**<br>Mk1 ≠ Mk2
Symmetry	0	0	0	0
Fusion	1	1	0	0
Phyllotaxis	0	4	5	0
Merism	0	0	0	0
Differentiation	1	1	5	0

Table SN9: Number of changes in inferred most likely ancestral state between models and tree types across 54 botanical orders. Each column compares the results of two of the model and tree type combinations and reports the number of nodes that change most likely state in each case. Mk1= one rate maximum likelihood model; Mk2= two-rate maximum likelihood model; full= full tree; part= partitioned tree.

54 nodes	**Mk1**<br>(full) ≠ (part)	**Mk2**<br>(full) ≠ (part)	**(full)**<br>Mk1 ≠ Mk2	**(part)**<br>Mk1 ≠ Mk2
Symmetry	0	1	1	0
Fusion	2	3	1	1
Phyllotaxis	1	7	7	1
Merism	0	0	1	0
Differentiation	1	7	15	5

Constrained Mk1 rates. The states of all nodes under constrained rates can be found in Supplementary Tables S3 (15 key nodes) and S4 (54 order nodes). The number of state-changing nodes is either identical or lower for the pruned Smith & Brown tree compared to our original ultrametric tree.

Parsimony reconstructions. A decrease in step number compared to our original tree was found in the pruned Smith & Brown tree for all characters except fusion (Table SN10). There are, however, small changes in the distribution of steps among origins and reversals, and among would-be partitions. The reduction of step number affects different aspects depending on the character: the number represents a lower number of minimum origins for both symmetry and differentiation, but accompanied by an increase in maximum reversal number in the former case. Merism has fewer reversals while keeping a stable number of origins, and phyllotaxis has losses in both origins and reversals. A per-partition breakdown can be found in Supplementary Tables 6.

Table SN10: Number of transitions with origin and reversal number range.

	State changes	Origins (min-max)	Reversals (min-max)
symmetry	207	130-173	34-77
fusion	212	70-135	77-142
phyllotaxis	28	21-27	1-7
pentamery	215	25-67	148-190
differentiation	151	33-66	85-118

Testing the impact of morphological rate heterogeneity on ancestral state reconstruction of five floral traits in angiosperms

Supplementary Discussion: Ancestral state reconstruction

Elisabeth Reyes, Sophie Nadot, Maria von Balthazar, Jürg Schönenberger, Hervé Sauquet

Supplementary Discussion: Ancestral state reconstruction

The Sauquet et al. [1] study is so far the only other study with a similarly large taxon sample. However, its sampling strategy was entirely guided by a previous study of angiosperm divergence times (792 species selected based on genetic marker availability in GenBank, independent of floral traits; Magallón et al. [2]), whereas our sampling was specifically designed to include most of the variation in five perianth traits, resulting in a data sample of 1230 species. Nevertheless, Sauquet et al. [1] reconstructed ancestral states, using three methods including parsimony and ML, for 21 characters on 15 key nodes identical to ours. Thus, we were able to compare our ML transition rates and symmetry ratios, as well as the state of the ancestral flower for all five of our test characters to those found by Sauquet et al. [1]. Transition rates were higher in our study than in Sauquet et al. [1] (about twice as high) and can be attributed to the fact that our sampling strategy favoured state diversity within clades, thus creating a bias towards higher transition rates. Despite this, symmetry ratios were largely equal for comparable characters (except for perianth fusion) [1]. Furthermore, the 15 shared key nodes have generally the same ancestral states for comparable characters. Both studies found the ancestral flower to have had a free, actinomorphic, whorled and undifferentiated perianth; merism was reconstructed as non-pentamerous in our study and as trimerous in Sauquet et al. [1].

Magallón et al. [3] estimated molecular substitution rates across a range of plastid coding genes in land plants. They found rates ranging from 0.0001 to 0.01 substitutions per million years. Such numbers are similar to the rates at which relevant inference of ancestral states was possible in our own study, but several orders of magnitude under some of the highest rates we found in some partitions, indicating the latter to be biologically implausible; any genetically-coded morphological change requires at least one mutation to happen.

The evolution of phyllotaxis, merism and differentiation can partially also be compared to Endress and Doyle [4], Ronse de Craene et al. [5], Zanis et al. [6] and Soltis et al. [7] while aspects of perianth fusion can be compared to Doyle and Endress [8]. However, it has to be kept in mind that the taxon sampling of these studies is generally less broad. In particular, these studies do not include Pentapetalae at all or only a limited number of representatives that show little to no variation in regards to the characters of this study. These reasons lead to a consistently lower number of origins of the derived state in these studies compared to our broadly and densely sampled study (e.g. less than ten changes in the entire tree, when the lowest number of changes for a character in our study is 31 for phyllotaxis).

Zygomorphy is here reconstructed to have originated at least 148 times in the angiosperms, with 69 reversals to actinomorphy. Our previous study on symmetry (Reyes et al. [9]), which used a similar sampling strategy, found 130 origins and 69 reversals. The extra steps found here are all origins, while the

number of reversals remains equal. Upon closer examination, it appears that accounting for the variation of five characters instead of only one as in Reyes et al. [9] results in more numerous and larger polytomies representing polymorphic non-resolved clades. Concurrently, most of the polytomies in which symmetry varies are reconstructed as ancestrally actinomorphic, making each zygomorphic species present in them its own origin. In this way, some of the additional origins can be explained. The present study agrees with Citerne et al. [10], Reyes et al. [9] and Sauquet et al. [1] that all key nodes are actinomorphic and transitions to zygomorphy are more frequent above order level to the point that only three of the order nodes from the present study can potentially be reconstructed as zygomorphic (Commelinales, Zingiberales and Lamiales).

We find a minimum of 71 origins of perianth fusion and 141 reversals to a free perianth. In our study, the Mk1 and Mk2 models are similarly fitted, meaning that transitions from free to fused happen almost as frequently as transitions from fused to free for the character. In the maximum likelihood reconstruction of Sauquet et al. [1] , however, the fit of models makes Mk2 a better choice than Mk1. Thus, our method found a higher reversal to origination ratio than Sauquet et al. [1] under maximum likelihood. Nevertheless, the symmetry ratio is of 0.80 in Sauquet et al. [1] while ours is 0.86, which make perianth fusion a labile state in both reconstructions. Furthermore, the perianth is ancestrally free in angiosperms and matches the results of Doyle and Endress [8] and Sauquet et al. [1]. At key nodes, however, our study usually finds fusion to have unequivocally occurred at deeper nodes within Pentapetalae (Superrosidae and the common ancestor between Asteridae and Caryophyllales) than Sauquet et al. [1] (Lamiidae, Campanulidae and various origins at order level or lower in the other clades), which is reflected in our higher symmetry ratio between rates.

Spiral phyllotaxis originated a minimum of 23 times, of which 15 origins are found in [ANA+Magnoliidae] and basal eudicots. Reversals to whorled phyllotaxis occurred eight times, three of which remain even in the scenario with the maximum number of origins. Our parsimony results overall agree with previous parsimony reconstructions of perianth phyllotaxis [1,4–8,11,12] in that the common ancestor of angiosperms remains equivocal. However, most of our ML reconstructions find the common ancestor of angiosperms to be whorled, consistent with the Bayesian results of the recent study by Sauquet et al. [1]. Among the models we tested, only Mk2 fitted to the full tree reconstructs the angiosperms as ancestrally spiral. This very same model reconstructs Monocotyledoneae as ancestrally spiral with moderate support despite the fact they do not contain any spiral species. Mk2 fitted to the full tree may hence represent a case of the artificially high inferred reversal rate artefact discussed above. We suspect spiral floral phyllotaxis to be a case of macroevolutionary self-destruction, as its appearance leads to lower diversifications of these groups compared to closely related groups [13]. Self-destructing traits are characterized by a tendency to be present in several small clades at the tips of the phylogenetic tree [13].

Indeed, spiral phyllotaxis shows this phenomenon, especially in Pentapetalae, where it appears only in eight relatively small clades in our study (e.g., Paeoniaceae, Theaceae).

We find at least 25 origins of a pentamerous perianth, of which 15 are in Pentapetalae and five in [ANA+Magnoliidae]. The origins of pentapetaly in the Pentapetalae clade are reversals to an ancestral pentamerous state (e.g., Fagales). The 25 origins are accompanied by 201 reversals, of which 194 are in Pentapetalae. Outside Pentapetalae, pentamery originated in Hernandiaceae (Magnoliidae) as well as in Ranunculaceae and in Sabiaceae (basal eudicots) as also reported by previous studies [1,5–7,14]. In the present study, we find additional origins in Canellales, Magnoliaceae, Siparunaceae (all Magnoliidae), Stemonaceae (Monocotyledoneae), and Platanaceae (basal eudicots) (Figure S1).

We found a minimum of 44 origins of perianth differentiation, 34 of which are in [ANA+Magnoliidae] and Monocotyledoneae, accompanied by 117 reversals. Most earlier reconstructions of perianth differentiation [1,5–8,15] agree that angiosperms ancestrally had an undifferentiated perianth. In these studies, Nymphaeales, Magnoliales, Canellales, Piperales and Alismatales all either had independent origins of a differentiated perianth or reconstructions left the ancestral state equivocal. In the studies with a dense sampling of the non-Alismatales monocots, Sauquet et al. [1], Ronse de Craene [5] and the present study, Asparagales and Liliales have their own origin(s) of perianth differentiation as a definite or equivocal scenario. The results for [[Commelinales+Zingiberales]+Poales] are less consensual: our study gives them an unequivocal common origin, Sauquet et al. [1] suggest a separate origin for each order (and Arecales sister only to Poales instead of all three), while Ronse de Craene [5] is equivocal for both possibilities. Our study shows high sensitivity of character state reconstruction within the non-Pentapetalae, whereas Pentapetalae remain stable under all models and show an ancestrally differentiated perianth that reverts to an undifferentiated state in a few orders and families. The ancestral states reconstructed in the Mk2 version of the full tree resemble those from Ronse De Craene [5], Soltis et al. [7] and Sauquet et al.[1], while the Mk1 full tree reconstructed ancestral states resemble those of Endress and Doyle [4], which infers differentiation to be ancestral for all angiosperms except Amborellales.

1. Sauquet, H. *et al.* The ancestral flower of angiosperms and its early diversification. *Nat. Commun.* **8,** 16047 (2017).
2. Magallón, S., Gómez-Acevedo, S., Sánchez-Reyes, L. L. & Hernández-Hernández, T. A metacalibrated time-tree documents the early rise of flowering plant phylogenetic diversity. *New Phytol.* **207,** 437–453 (2015).
3. Magallón, S., Hilu, K. W. & Quandt, D. Land plant evolutionary timeline: gene effects are secondary to fossil constraints in relaxed clock estimation of age and substitution rates. *Am. J. Bot.* **100,** 556–573 (2013).
4. Endress, P. K. & Doyle, J. A. Reconstructing the ancestral angiosperm flower and its initial specializations. *Am. J. Bot.* **96,** 22–66 (2009).

5. Ronse De Craene, L. P., Soltis, P. S. & Soltis, D. E. Evolution of floral structures in basal angiosperms. *Int. J. Plant Sci.* **164,** S329–S363 (2003).

6. Zanis, M. J., Soltis, P. S., Qiu, Y. L., Zimmer, E. & Soltis, D. E. Phylogenetic analyses and perianth evolution in basal angiosperms. *Ann. Missouri Bot. Gard.* **90,** 129–150 (2003).

7. Soltis, D. E., Soltis, P. S., Endress, P. K. & Chase, M. W. *Phylogeny and evolution of angiosperms*. (Sinauer Associates, 2005).

8. Doyle, J. A. & Endress, P. K. Morphological phylogenetic analysis of basal angiosperms: comparison and combination with molecular data. *Int. J. Plant Sci.* **161,** S121–S153 (2000).

9. Reyes, E., Sauquet, H. & Nadot, S. Perianth symmetry changed at least 199 times in angiosperm evolution. *Taxon* **65,** 945–964 (2016).

10. Citerne, H., Jabbour, F., Nadot, S. & Damerval, C. The evolution of floral symmetry. *Adv. Bot. Res.* **54,** 85–136 (2010).

11. Doyle, J. A. & Endress, P. K. Tracing the early evolutionary diversification of the angiosperm flower. in *Flowers on the Tree of Life* (eds. Wanntorp, L. & Ronse De Craene, L. P.) 88–119 (Cambridge University Press, 2011).

12. Endress, P. K. & Doyle, J. A. Floral phyllotaxis in basal angiosperms: development and evolution. *Curr. Opin. Plant Biol.* **10,** 52–57 (2007).

13. Bromham, L., Hua, X. & Cardillo, M. Detecting macroevolutionary self-destruction from phylogenies. *Syst. Biol.* **65,** 109–127 (2016).

14. Soltis, D. E. *et al.* Gunnerales are sister to other core eudicots: Implications for the evolution of pentamery. *Am. J. Bot.* **90,** 461–470 (2003).

15. Hileman, L. C. & Irish, V. F. More is better: the uses of developmental genetic data to reconstruct perianth evolution. *Am. J. Bot.* **96,** 83–95 (2009).

Testing the impact of morphological rate heterogeneity on ancestral state reconstruction of five floral traits in angiosperms

Supplementary Methods 1: Choice of nodes

Elisabeth Reyes, Sophie Nadot, Maria von Balthazar, Jürg Schönenberger, Hervé Sauquet

Supplementary Methods 1: Choice of nodes

The set of 15 key nodes is the same as the ones from Sauquet et al. [31]. It consists of Angiospermae, Mesangiospermae, Magnoliidae, Monocotyledoneae, Commelinidae, Eudicotyledoneae, Pentapetalae Superrosidae, Rosidae, Malvidae, Fabidae, Superasteridae, Asteridae, Campanulidae and Lamiidae. Mesangiospermae and Pentapetalae can be reconstructed on the full tree, but not the partitioned tree, where these nodes disappear (by pruning of partition subtrees). More details can be found in table SM1 (see also Fig. 4).

Table SM1: Definition of key nodes and partition to which they belong

Node name	Includes	Partition
Angiospermae	All angiosperms	[ANA+Magnoliidae] (partition 1)
Mesangiospermae	All angiosperms except ANA grade	Absent (descendants belong to different partitions)
Magnoliidae	Set of related orders	[ANA+Magnoliidae] (partition 1)
Monocotyledoneae	Monocotyledoneae	Monocotyledoneae (partition 2)
Commelinidae	Set of related orders	Monocotyledoneae (partition 2)
Eudicotyledoneae	Basal eudicots, Superrosidae, Superasteridae	Ceratophyllales + basal eudicots (partition 3)
Pentapetalae	Superrosidae, Superasteridae	Absent (descendants belong to different partitions)
Superrosidae	Rosidae, other orders	Superrosidae (partition 4)
Rosidae	Malvidae, Fabidae, other orders	Superrosidae (partition 4)
Malvidae	Set of related orders	Superrosidae (partition 4)
Fabidae	Set of related orders	Superrosidae (partition 4)
Superasteridae	Asteridae, other orders	Superasteridae (partition 5)
Asteridae	Campanulidae, Lamiidae, other orders	Superasteridae (partition 5)
Campanulidae	Set of related orders	Superasteridae (partition 5)
Lamiidae	Set of related orders	Superasteridae (partition 5)

The set of 54 order nodes consists of the most recent common ancestors of 54 of the 64 orders. Ten orders were not included due to being entirely perianthless, monospecific, or both monofamilial and monomorphic with respect to all five characters. All these factors resulted in them having only one representative in our tree and hence no node to reconstruct. Those orders are Amborellales and Chloranthales for partition 1, Acorales for partition 2, Ceratophyllales, Trochodendrales and Gunnerales for partition 3, Pircramniales for partition 4 and Dilleniales, Metteniusales and Vahliales for partition 5.

Testing the impact of morphological rate heterogeneity on ancestral state reconstruction of five floral traits in angiosperms

Supplementary Methods 2: Making the pruned Smith & Brown tree

Elisabeth Reyes, Sophie Nadot, Maria von Balthazar, Jürg Schönenberger, Hervé Sauquet

Supplementary Methods 2: Making the pruned Smith & Brown tree

The second tree on which we reconstructed ancestral states with the rescored states is a pruned and modified version of the Smith & Brown [16] ALLMB tree. This dated tree was reconstructed by the authors using a hierarchical clustering analysis of GenBank data (using an updated implementation of the PHLAWD pipeline), supplemented with taxonomic knowledge from the Open Tree of Life to graft taxa lacking suitable molecular data, and dated using treePL with secondary (fixed-age) calibrations drawn from all overlapping nodes with the dated tree of Magallón et al. [2]. As a result, Smith & Brown obtained an extremely large dated tree of 353,185 taxa, presumably including all known species of seed plants. Although such mega-trees contain limitations and unavoidable errors inherent to this approach, our own assessment is that this particular tree is of sufficient quality to explore the sensitivity of our results to the hand-crafted tree used in our main analyses. Importantly, the two trees should differ in specific branch lengths outside the calibrated nodes, as well as in topology for both some weakly supported deep nodes and some shallow nodes.

Among the 1230 species sampled in our dataset, 1183 were found in the initial ALLMB tree. Another 32 species were represented by at least one member of the same genus, or of the same small family in the initial ALLMB tree, which we used as placeholders for these species (on the assumption that these genera and small families are monophyletic). The last 15 species in our sample could not easily be replaced by taxa from the ALLMB tree and were discarded from these analyses. The initial ALLMB tree was thus pruned and transformed to a tree of 1215 species with names matching those of our dataset. The node names were left intact, but largely ignored in favour of those used in the main article to preserve the comparability of the states between the trees.

The results from these new analyses are reported in Supplementary Note 2. The pruned tree (referred to in this study as PrunedMB) is available in NEXUS format as Supplementary Data 3.

2. Magallón, S., Gómez-Acevedo, S., Sánchez-Reyes, L. L. & Hernández-Hernández, T. A metacalibrated time-tree documents the early rise of flowering plant phylogenetic diversity. *New Phytol.* **207,** 437–453 (2015)

16. Smith, S. A. & Brown, J. W. Constructing a broadly inclusive seed plant phylogeny. *Am. J. Bot.* **105,** 1–13 (2018).

Testing the impact of morphological rate heterogeneity on ancestral state reconstruction of five floral traits in angiosperms

Supplementary Tables 1: Parsimony steps, Mk1 and Mk2 transition rates, Mk2 rate symmetry ratios, logLikelihoods and AICs for the full tree and partitions

Elisabeth Reyes, Sophie Nadot, Maria von Balthazar, Jürg Schönenberger, Hervé Sauquet

Supplementary Tables 1: Parsimony steps, Mk1 and Mk2 transition rates, Mk2 rate symmetry ratios, logLikelihoods and AICs for the full tree and partitions

Green: transition rates too high to infer most likely states
Red: no state change at all on that part of the tree
Five-char: Results for main tree
PrunedMB: Results for the pruned Smith & Brown ALLMB tree

Symmetry

Parsi= parsimony; 0= actinomorphic; 1= zygomorphic; q= transition rate (changes per Ma)

RESCORED DATA (Five-char)	Angiosperms	ANA+ Magnoliidae	Monocotyledoneae	Basal eudicots	Superrosidae	Superasteridae	Mixed partitions
Parsi steps	217	3	40	23	63	88	
Mk1 q	0.0068	0.0008	0.1041	0.2640	0.0045	0.0084	
Mk1 logLik	-713.33	-14.41	-114.34	-67.92	-192.73	-280.86	-670.26
Mk1 AIC	1428.66	30.82	230.68	137.84	387.46	563.72	1350.52
Mk2 q01	0.0072	0.0059	0.0663	0.0126	0.0046	0.009	
Mk2 q10	0.0286	0.1116	0.1294	0.0359	0.0219	0.0317	
symmetry ratio	0.25	0.05	0.51	0.35	0.21	0.28	
Mk2 logLik	-627.83	-11.91	-105.67	-64.35	-172.91	-248.24	-603.08
Mk2 AIC	1259.66	27.82	215.34	132.70	349.82	500.48	1226.16
Δ AIC	169.00	3.00	15.34	5.14	37.64	63.24	124.36

Δ AIC for angiosperms and mixed partitions: 78.14 (Mk1); 33.50 (Mk2)

ORIGINAL DATA (Five-char)	Angiosperms	ANA+ Magnoliidae	Monocotyledoneae	Basal eudicots	Superrosidae	Superasteridae	Mixed partitions
Parsi steps	191	3	38	20	55	75	
Mk1 q	0.0056	0.0008	0.1261	0.0303	0.0043	0.0064	
Mk1 logLik	-647.63	-14.41	-112.28	-66.23	-188.92	-249.85	-631.69
Mk1 AIC	1297.26	30.82	226.56	134.46	379.84	501.70	1273.38
Mk2 q01	0.0060	0.0059	0.0992	0.0138	0.0043	0.0079	
Mk2 q10	0.0259	0.1116	0.2040	0.0392	0.0215	0.0302	
symmetry ratio	0.23	0.05	0.49	0.35	0.20	0.26	
Mk2 logLik	-581.77	-11.91	-102.41	-60.68	-168.86	-225.33	-569.19
Mk2 AIC	1167.54	27.82	208.82	125.36	341.72	454.66	1158.38
Δ AIC	129.72	3.00	17.74	9.10	38.12	47.04	115.00

Δ AIC for angioperms and mixed partitions: 28.33 (Mk1); 9.16 (Mk2)

RESCORED DATA (PrunedMB)	Angiosperms	ANA+ Magnoliidae	Monocotyledoneae	Basal eudicots	Superrosidae	Superasteridae	Mixed partitions
Parsi steps	207	3	42	22	59	81	
Mk1 q	0.0066	0.0008	75.0553	0.1756	0.0049	0.008	
Mk1 logLik	-695.64	-15.03	-113.68	-67.67	-199.15	-274.02	-669.55
Mk1 AIC	1393.28	32.06	229.36	137.34	400.3	550.04	1341.1
Mk2 q01	0.0074	0.0368	7.0106	0.0071	0.0055	0.0087	
Mk2 q10	0.0285	0.6851	13.8936	0.0312	0.0233	0.0309	
symmetry ratio	0.26	0.01	0.5	0.23	0.24	0.28	
Mk2 logLik	-618.28	-11.86	-104.62	-61.37	-179.46	-245.94	-603.25
Mk2 AIC	1240.56	27.72	213.24	126.74	362.92	495.88	1210.5
Δ AIC	152.72	4.34	16.12	10.6	37.38	54.16	130.6

Δ AIC for angiosperms and mixed partitions: 52.18 (Mk1); 30.06 (Mk2)

ORIGINAL DATA (PrunedMB)	Angiosperms	ANA+ Magnoliidae	Monocotyledoneae	Basal eudicots	Superrosidae	Superasteridae	Mixed partitions
Parsi steps	188	3	40	20	57	68	
Mk1 q	0.0057	0.0008	75.0553	0.2063	0.0046	0.0059	
Mk1 logLik	-644.99	-15.03	-111.6	-64.96	-195.06	-239	-625.65
Mk1 AIC	1291.98	32.06	225.2	131.92	392.12	480	1253.3
Mk2 q01	0.0068	0.0037	6.4502	0.0067	0.0051	0.0073	
Mk2 q10	0.0275	0.6851	13.5206	0.0329	0.0225	0.0286	
symmetry ratio	0.25	0.01	0.48	0.20	0.23	0.26	
Mk2 logLik	-581.05	-11.86	-101.28	-58.08	-175.54	-219.53	-566.29
Mk2 AIC	1166.1	27.72	206.56	120.16	355.08	443.06	1136.58
Δ AIC	125.88	4.34	18.64	11.76	37.04	36.94	116.72

Δ AIC for angioperms and mixed partitions: 38.68 (Mk1); 29.52 (Mk2)

Fusion

Parsi= parsimony; 0= free; 1= fused; q= transition rate (changes per Ma)

RESCORED DATA (Five-char)	Angiosperms	ANA+ Magnoliidae	Monocotyledoneae	Basal eudicots	Superrosidae	Superasteridae	Mixed partitions
Parsi steps	212	19	38	16	76	62	
Mk1 q	0.0103	7.1740	0.0149	0.0090	0.0672	0.0058	
Mk1 logLik	-549.68	-43.67	-93.93	-56.02	-159.28	-175.40	-528.30
Mk1 AIC	1101.36	89.34	189.86	114.04	320.56	352.80	1066.60
Mk2 q01	0.0109	5.0520	0.0127	0.0073	0.0670	0.0089	
Mk2 q10	0.0094	9.4200	0.0194	0.0439	0.0643	0.0057	
symmetry ratio	0.86	0.54	0.65	0.17	0.96	0.64	
Mk2 logLik	-548.84	-40.76	-91.99	-45.91	-159.24	-174.50	-512.40
Mk2 AIC	1101.68	85.52	187.98	95.82	322.48	353.00	1044.80
Δ AIC	0.32	3.82	1.88	18.22	1.92	-0.20	21.80

Δ AIC for angiosperms and mixed partitions: 34.76 (Mk1); 56.88 (Mk2)

ORIGINAL DATA (Five-char)	Angiosperms	ANA+ Magnoliidae	Monocotyledoneae	Basal eudicots	Superrosidae	Superasteridae	Mixed partitions
Parsi steps	154	13	34	6	54	47	
Mk1 q	0.0072	0.0056	0.0120	0.0026	0.0121	0.0055	
Mk1 logLik	-431.15	-37.21	-85.49	-32.35	-128.68	-140.35	-424.08
Mk1 AIC	864.30	76.42	172.98	66.70	259.36	282.70	858.16
Mk2 q01	0.0069	0.3581	0.0121	0.0030	0.0323	0.0091	
Mk2 q10	0.0082	0.9789	0.0222	0.0515	0.0484	0.003	
symmetry ratio	0.84	0.37	0.55	0.06	0.67	0.33	
Mk2 logLik	-430.55	-32.54	-81.91	-23.64	-126.11	-139.18	-403.38
Mk2 AIC	865.10	69.08	167.82	51.28	256.22	282.36	826.76
Δ AIC	0.80	7.34	5.16	15.42	3.14	0.34	31.40

Δ AIC for angiosperms and mixed partitions: 6.14 (Mk1); 38.34 (Mk2)

RESCORED DATA (PrunedMB)	Angiosperms	ANA+ Magnoliidae	Monocotyledoneae	Basal eudicots	Superrosidae	Superasteridae	Mixed partitions
Parsi steps	212	17	39	16	81	58	
Mk1 q	0.0114	0.1315	5.5324	0.0084	0.0355	0.0058	
Mk1 logLik	-561.79	-42.56	-94.27	-55.32	-159	-182.31	-533.46

**Mk1 AIC**	1125.58	87.12	190.54	112.64	320	366.62	1068.92
**Mk2 q01**	0.0123	0.0814	4.5855	0.0068	0.0347	0.0112	
**Mk2 q10**	0.0099	0.1507	6.1667	0.0422	0.0318	0.0057	
**symmetry ratio**	0.80	0.54	0.74	0.16	0.92	0.51	
**Mk2 logLik**	-559.71	-39.85	-92.79	-46.28	-158.8	-180.07	-517.79
**Mk2 AIC**	1123.42	83.7	189.58	96.56	321.6	364.14	1039.58
**Δ AIC**	-2.16	3.42	0.96	16.08	1.6	2.48	29.34

Δ AIC for angiosperms and mixed partitions: 56.66 (Mk1); 83.84 (Mk2)

ORIGINAL DATA (PrunedMB)	Angiosperms	ANA+ Magnoliidae	Monocotyledoneae	Basal eudicots	Superrosidae	Superasteridae	Mixed partitions
**Parsi steps**	155	13	35	6	54	47	
**Mk1 q**	0.0078	0.1283	4.868	0.0025	0.0116	0.006	
**Mk1 logLik**	-437.37	-37.86	-86.64	-30.6	-123.28	-144.24	-422.62
**Mk1 AIC**	876.74	77.72	175.28	63.2	248.56	290.48	847.24
**Mk2 q01**	0.0077	0.0903	3.7679	0.0035	0.013	0.0093	
**Mk2 q10**	0.0085	0.2386	6.2532	0.057	0.0165	0.0036	
**symmetry ratio**	0.91	0.38	0.60	0.06	0.78	0.39	
**Mk2 logLik**	-437.16	-32.14	-82.76	-23.6	-122.84	-143.11	-404.45
**Mk2 AIC**	878.32	68.28	169.52	51.2	249.68	290.22	812.9
**Δ AIC**	1.58	9.44	5.76	12	1.12	0.26	34.34

Δ AIC for angiosperms and mixed partitions: 29.50 (Mk1); 65.42 (Mk2)

Phyllotaxis

Parsi= parsimony; 0= whorled; 1= fused; q= transition rate (changes per Ma)

RESCORED DATA (Five-char)	Angiosperms	ANA+ Magnoliidae	Monocotyledoneae	Basal eudicots	Superrosidae	Superasteridae	Mixed partitions
Parsi steps	31	11	0	12	4	4	
Mk1 q	0.0006	0.0035	0.0000	0.0047	0.0002	0.0002	
Mk1 logLik	-154.39	-35.07	-0.69	-40.05	-23.83	-24.23	-123.87
Mk1 AIC	310.78	72.14	3.38	82.10	49.66	50.46	257.74
Mk2 q01	0.0003	0.0034	1.08E-12	0.0033	0.0008	0.0004	
Mk2 q10	0.015	0.003	7.51E-07	0.0188	0.0709	0.035	
symmetry ratio	0.02	0.88	695370.37	0.18	0.01	0.01	
Mk2 logLik	-128.77	-35.06	0.00	-34.67	-22.23	-22.41	-114.37
Mk2 AIC	261.54	74.12	4.00	73.34	48.46	48.82	248.74
Δ AIC	49.24	1.98	0.62	8.76	1.20	1.64	9.00

Δ AIC for angiosperms and mixed partitions: 53.04 (Mk1); 12.80 (Mk2)

ORIGINAL DATA (Five-char)	Angiosperms	ANA+ Magnoliidae	Monocotyledoneae	Basal eudicots	Superrosidae	Superasteridae	Mixed partitions
Parsi steps	29	11	0	11	3	4	
Mk1 q	0.0006	0.0034	0.0000	0.0041	0.0002	0.0002	
Mk1 logLik	-142.36	-34.43	-0.69	-35.32	-18.36	-24.23	-113.03
Mk1 AIC	286.72	70.86	3.38	72.64	38.72	50.46	236.06
Mk2 q01	0.0003	0.0034	0.0000	0.0037	0.0005	0.0004	
Mk2 q10	0.0157	0.0029	0.0000	0.0245	0.0656	0.0350	
symmetry ratio	0.02	0.85	695370.37	0.15	0.01	0.01	
Mk2 logLik	-119.75	-34.42	0.00	-30.66	-17.52	-22.41	-105.01
Mk2 AIC	243.50	72.84	4.00	65.32	39.04	48.82	230.02
Δ AIC	43.22	1.98	0.62	7.32	0.32	1.64	6.04

Δ AIC for angiosperms and mixed partitions: 50.66 (Mk1); 13.48 (Mk2)

RESCORED DATA (PrunedMB)	Angiosperms	ANA+ Magnoliidae	Monocotyledoneae	Basal eudicots	Superrosidae	Superasteridae	Mixed partitions
Parsi steps	28	10	0	10	4	4	
Mk1 q	0.0006	0.004	1.03E-10	0.0042	0.0002	0.0002	
Mk1 logLik	-146.98	-33.59	-0.69	-37.31	-23.03	-25.14	-119.76

**Mk1 AIC**	295.96	69.18	3.38	76.62	48.06	52.28	241.52
**Mk2 q01**	0.0003	0.0058	1.08E-12	0.0028	0.0006	0.0007	
**Mk2 q10**	0.0149	0.0175	7.51E-07	0.0196	0.0485	0.0681	
**symmetry ratio**	0.02	0.33	6.95E+05	0.14	0.01	0.01	
**Mk2 logLik**	-120.35	-31.92	-1.44E-06	-31.47	-22.02	-22.28	-107.69
**Mk2 AIC**	244.7	67.84	4.00	66.94	48.04	48.56	219.38
**Δ AIC**	51.26	-1.34	0.62	9.68	0.02	3.72	22.14

Δ AIC for angiosperms and mixed partitions: 54.44 (Mk1); 25.32 (Mk2)

**ORIGINAL DATA (PrunedMB)**	**Angiosperms**	**ANA+ Magnoliidae**	**Monocotyledoneae**	**Basal eudicots**	**Superrosidae**	**Superasteridae**	**Mixed partitions**
**Parsi steps**	25	10	0	8	3	4	
**Mk1 q**	0.0005	0.0038	1.03E-10	0.0033	0.0002	0.0002	
**Mk1 logLik**	-131.86	-32.3	-0.69	-31.51	-17.58	-25.14	-107.22
**Mk1 AIC**	265.72	66.6	3.38	65.02	37.16	52.28	216.44
**Mk2 q01**	0.0003	0.0058	1.08E-12	0.0027	0.0002	0.0007	
**Mk2 q10**	0.016	0.0176	7.51E-07	0.0265	0.0023	0.0681	
**symmetry ratio**	0.02	0.33	6.95E+05	0.1	0.09	0.01	
**Mk2 logLik**	-110.66	-30.99	-1.44E-06	-26.78	-17.01	-22.28	-97.06
**Mk2 AIC**	225.32	65.98	4.00000288	57.56	38.02	48.56	198.12
**Δ AIC**	40.4	0.62	0.62000288	7.46	0.86	3.72	18.32

Δ AIC for angiosperms and mixed partitions: 49.28 (Mk1); 27.20 (Mk2)

Merism

Parsi= parsimony; 0= non-pentamerous; 1= pentamerous; q= transition rate (changes per Ma)

RESCORED DATA (Five-char)	Angiosperms	ANA+ Magnoliidae	Monocotyledoneae	Basal eudicots	Superrosidae	Superasteridae	Mixed partitions
Parsi steps	226	6	1	9	101	108	
Mk1 q	0.0068	0.0024	0.0001	0.0040	0.0111	0.0113	
Mk1 logLik	-657.41	-21.56	-7.43	-34.43	-250.00	-285.17	-598.59
Mk1 AIC	1316.82	45.12	16.86	70.86	502.00	572.34	1207.18
Mk2 q01	0.0026	0.0041	0.0005	0.0020	0.0240	0.0464	
Mk2 q10	0.0073	0.0224	0.0715	0.0137	0.0129	0.018	
symmetry ratio	0.36	0.18	0.01	0.15	0.54	0.39	
Mk2 logLik	-644.41	-19.90	-6.09	-29.69	-240.31	-259.29	-555.28
Mk2 AIC	1292.82	43.80	16.18	63.38	484.62	522.58	1130.56
Δ AIC	24.00	1.32	0.68	7.48	17.38	49.76	76.62

Δ AIC for angiosperms and mixed partitions: 109.64 (Mk1); 162.26 (Mk2)

ORIGINAL DATA (Five-char)	Angiosperms	ANA+ Magnoliidae	Monocotyledoneae	Basal eudicots	Superrosidae	Superasteridae	Mixed partitions
Parsi steps	199	3	1	7	88	99	
Mk1 q	0.0064	0.0011	0.0001	0.0030	0.0113	0.0110	
Mk1 logLik	-586.58	-13.16	-7.43	-29.46	-216.65	-257.56	-524.26
Mk1 AIC	1175.16	28.32	16.86	60.92	435.30	517.12	1058.52
Mk2 q01	0.0017	0.0039	0.0005	0.0013	0.0167	0.0306	
Mk2 q10	0.0077	0.0540	0.0715	0.0120	0.0109	0.0137	
symmetry ratio	0.22	0.07	0.01	0.11	0.65	0.45	
Mk2 logLik	-564.63	-11.00	-6.09	-24.61	-213.80	-243.34	-498.84
Mk2 AIC	1133.26	26.00	16.18	53.22	431.60	490.68	1017.68
Δ AIC	41.90	2.32	0.68	7.70	3.70	26.44	40.84

Δ AIC for angiosperms and mixed partitions: 116.64 (Mk1); 115.68 (Mk2)

RESCORED DATA (PrunedMB)	Angiosperms	ANA+ Magnoliidae	Monocotyledoneae	Basal eudicots	Superrosidae	Superasteridae	Mixed partitions
Parsi steps	215	6	1	9	98	100	
Mk1 q	0.007	0.0027	0.0001	0.0041	0.0138	96.796	

**Mk1 logLik**	-697.35	-20.28	-7.09	-35.8	-265.7	-295.66	-624.53
**Mk1 AIC**	1396.7	42.56	16.18	73.6	533.4	593.32	1251.06
**Mk2 q01**	0.0034	0.0049	0.0003	0.002	0.0308	81.6345	
**Mk2 q10**	0.0074	0.0225	0.0446	0.014	0.0176	32.3754	
**symmetry ratio**	0.46	0.22	0.01	0.14	0.57	0.4	
**Mk2 logLik**	-688.31	-19.12	-6.02	-30.65	-254.54	-254.58	-564.91
**Mk2 AIC**	1380.62	42.24	16.04	65.3	513.08	513.16	1133.82
**Δ AIC**	16.08	0.32	0.14	8.3	20.32	67.2	117.24

Δ AIC for angiosperms and mixed partitions: 145.64 (Mk1); 246.8 (Mk2)

**ORIGINAL DATA (PrunedMB)**	**Angiosperms**	**ANA+ Magnoliidae**	**Monocotyledoneae**	**Basal eudicots**	**Superrosidae**	**Superasteridae**	**Mixed partitions**
**Parsi steps**	186	3	1	7	84	90	
**Mk1 q**	0.0064	0.0012	0.0001	0.0033	0.0134	0.0115	
**Mk1 logLik**	-610.63	-11.97	-7.09	-31.76	-223.58	-274.39	-548.79
**Mk1 AIC**	1223.26	25.94	16.18	65.52	449.16	550.78	1099.58
**Mk2 q01**	0.0024	0.0029	0.0003	0.0014	0.0198	83.0772	
**Mk2 q10**	0.0077	0.0315	0.0446	0.0145	0.014	37.4142	
**symmetry ratio**	0.31	0.09	0.01	0.1	0.71	0.45	
**Mk2 logLik**	-593.3	-10.52	-6.02	-25.77	-220.61	-241.89	-504.81
**Mk2 AIC**	1190.6	25.04	16.04	55.54	445.22	487.78	1013.62
**Δ AIC**	32.66	0.9	0.14	9.98	3.94	63	85.96

Δ AIC for angiosperms and mixed partitions: 123.68 (Mk1); 176.98 (Mk2)

Differentiation

Parsi= parsimony; 0= undifferentiated; 1= differentiated; q= transition rate (changes per Ma)

RESCORED DATA (Five-char)	Angiosperms	ANA+ Magnoliidae	Monocotyledoneae	Basal eudicots	Superrosidae	Superasteridae	Mixed partitions
Parsi steps	161	20	42	15	51	32	
Mk1 q	0.0041	0.0129	0.0126	0.0059	0.0034	0.0020	
Mk1 logLik	-516.70	-39.52	-104.11	-44.12	-173.26	-128.40	-489.41
Mk1 AIC	1035.40	81.04	210.22	90.24	348.52	258.80	988.82
Mk2 q01	0.0091	0.0137	0.013	0.0023	0.0094	0.0086	
Mk2 q10	0.0029	0.0126	0.0123	0.006	0.0034	0.0015	
symmetry ratio	0.32	0.92	0.95	0.38	0.36	0.17	
Mk2 logLik	-496.31	-39.49	-104.08	-43.5	-170.28	-121.63	-478.98
Mk2 AIC	996.62	82.98	212.16	91.00	344.56	247.26	977.96
Δ AIC	38.78	1.94	1.94	0.76	3.96	11.54	10.86

Δ AIC for angiosperms and mixed partitions: 46.58 (Mk1); 18.66 (Mk2)

ORIGINAL DATA (Five-char)	Angiosperms	ANA+ Magnoliidae	Monocotyledoneae	Basal eudicots	Superrosidae	Superasteridae	Mixed partitions
Parsi steps	159	19	42	16	50	32	
Mk1 q	0.0041	0.0118	0.0129	0.0059	0.0034	0.0020	
Mk1 logLik	-512.31	-38.62	-103.08	-44.12	-171.08	-128.28	-485.18
Mk1 AIC	1026.62	79.24	208.16	90.24	344.16	258.56	980.36
Mk2 q01	0.0088	0.0121	0.013	0.0023	0.0085	0.0086	
Mk2 q10	0.0028	0.0113	0.0126	0.0061	0.0033	0.0015	
symmetry ratio	0.32	0.93	0.97	0.38	0.39	0.17	
Mk2 logLik	-491.6	-38.6	-103.07	-43.5	-168.31	-121.54	-475.02
Mk2 AIC	987.20	81.20	210.14	91.00	340.62	247.08	970.04
delta AIC	39.42	1.96	1.98	0.76	3.54	11.48	10.32

Δ AIC for angiosperms and mixed partitions: 46.26 (Mk1); 17.16 (Mk2)

RESCORED DATA (PrunedMB)	Angiosperms	ANA+ Magnoliidae	Monocotyledoneae	Basal eudicots	Superrosidae	Superasteridae	Mixed partitions
Parsi steps	151	18	40	15	50	27	
Mk1 q	0.0041	5.6183	0.0132	0.006	0.0035	0.0018	
Mk1 logLik	-512.44	-39.51	-103.54	-43.69	-171.7	-120.04	-478.48

**Mk1 AIC**	1026.88	81.02	209.08	89.38	345.4	242.08	958.96
**Mk2 q01**	0.0094	6.3653	0.0136	0.0023	0.01	0.0099	
**Mk2 q10**	0.003	4.9728	0.0128	0.0064	0.0035	0.0013	
**symmetry ratio**	0.32	0.78	0.94	0.36	0.35	0.13	
**Mk2 logLik**	-492.08	-39.08	-103.5	-42.82	-167.93	-113.36	-466.69
**Mk2 AIC**	988.16	82.16	211	89.64	339.86	230.72	937.38
**Δ AIC**	38.72	1.14	1.92	0.26	5.54	11.36	21.58

Δ AIC for angiosperms and mixed partitions: 64.92 (Mk1); 50.78 (Mk2)

**ORIGINAL DATA (PrunedMB)**	**Angiosperms**	**ANA+ Magnoliidae**	**Monocotyledoneae**	**Basal eudicots**	**Superrosidae**	**Superasteridae**	**Mixed partitions**
**Parsi steps**	149	17	40	15	49	27	
**Mk1 q**	0.0041	6.477	0.0134	0.006	0.0034	0.0018	
**Mk1 logLik**	-504.19	-38.82	-102.38	-43.69	-169.23	-119.87	-473.99
**Mk1 AIC**	1010.38	79.64	206.76	89.38	340.46	241.74	949.98
**Mk2 q01**	0.0091	7.3	0.0137	0.0023	0.009	0.0099	
**Mk2 q10**	0.0029	5.887	0.013	0.0064	0.0034	0.0013	
**symmetry ratio**	0.32	0.81	0.95	0.36	0.38	0.13	
**Mk2 logLik**	-484.39	-38.49	-102.36	-42.82	-166.1	-113.24	-463.01
**Mk2 AIC**	972.78	80.98	208.72	89.64	336.2	230.48	930.02
**delta AIC**	37.6	1.34	1.96	0.26	4.26	11.26	19.96

Δ AIC for angiosperms and mixed partitions: 60.40 (Mk1); 42.76 (Mk2)

Testing the impact of morphological rate heterogeneity on ancestral state reconstruction of five floral traits in angiosperms

Supplementary Tables 2: Parsimony, Mk1 and Mk2 ancestral states for 15 key nodes

Elisabeth Reyes, Sophie Nadot, Maria von Balthazar, Jürg Schönenberger, Hervé Sauquet

Supplementary Tables 2: Parsimony, Mk1 and Mk2 ancestral states for 15 key nodes
Five-char: Results for main tree
PrunedMB: Results for the pruned Smith & Brown ALLMB tree
Asterisks in the maximum likelihood columns: none = weak support (0.51-0.60), one = moderate support (0.61-0.89), two = strong support (0.90-1)

Symmetry
high rate= high rate equilibrium; full = full tree; part = partitioned tree; **state differences between comparable models**

RESCORED DATA (Five-char)	Parsimony	Maximum Likelihood (Mk1 model)		Maximum Likelihood (Mk2 model)	
	Both "full" and "part"	Mk1 Likelihood (full)	Mk1 Likelihood (part)	Mk2 Likelihood (full)	Mk2 Likelihood (part)
Angiospermae	actinomorphic	actinomorphic**	actinomorphic**	actinomorphic*	actinomorphic**
Mesangiospermae	actinomorphic	actinomorphic**	?	actinomorphic*	?
Magnoliidae	actinomorphic	actinomorphic**	actinomorphic**	actinomorphic*	actinomorphic**
Monocotyledoneae	actinomorphic, zygomorphic	actinomorphic**	equivocal (high rate)	actinomorphic*	actinomorphic (high rate)
Commelinidae	actinomorphic	**actinomorphic****	equivocal (high rate)	**zygomorphic**	actinomorphic (high rate)
Eudicotyledoneae	actinomorphic	actinomorphic**	equivocal (high rate)	actinomorphic*	actinomorphic*
Pentapetalae	actinomorphic	actinomorphic**	?	actinomorphic*	?
Superrosidae	actinomorphic	actinomorphic**	actinomorphic**	actinomorphic*	actinomorphic**
Rosidae	actinomorphic	actinomorphic**	actinomorphic**	actinomorphic*	actinomorphic**
Malvidae	actinomorphic	actinomorphic**	actinomorphic**	actinomorphic*	actinomorphic*
Fabidae	actinomorphic	actinomorphic**	actinomorphic**	actinomorphic*	actinomorphic**
Superasteridae	actinomorphic	actinomorphic**	actinomorphic**	actinomorphic*	actinomorphic*
Asteridae	actinomorphic	actinomorphic**	actinomorphic**	actinomorphic*	actinomorphic*
Campanulidae	actinomorphic	actinomorphic**	actinomorphic**	actinomorphic*	actinomorphic*
Lamiidae	actinomorphic	**actinomorphic****	actinomorphic**	**equivocal**	**actinomorphic**

ORIGINAL DATA (Five-char)	Parsimony	Maximum Likelihood (Mk1 model)		Maximum Likelihood (Mk2 model)	
	Both "full" and "part"	Mk1 Likelihood (full)	Mk1 Likelihood (part)	Mk2 Likelihood (full)	Mk2 Likelihood (part)
Angiospermae	actinomorphic	actinomorphic**	actinomorphic**	actinomorphic*	actinomorphic**
Mesangiospermae	actinomorphic	actinomorphic**	?	actinomorphic*	?
Magnoliidae	actinomorphic	actinomorphic**	actinomorphic**	actinomorphic*	actinomorphic**
Monocotyledoneae	actinomorphic, zygomorphic	actinomorphic**	equivocal (high rate)	actinomorphic*	actinomorphic (high rate)

**Commelinidae**	actinomorphic	**actinomorphic****	equivocal (high rate)	**zygomorphic**	actinomorphic (high rate)
**Eudicotyledoneae**	actinomorphic	actinomorphic**	actinomorphic	actinomorphic*	actinomorphic*
**Pentapetalae**	actinomorphic	actinomorphic**	?	actinomorphic**	?
**Superrosidae**	actinomorphic	actinomorphic**	actinomorphic**	actinomorphic**	actinomorphic**
**Rosidae**	actinomorphic	actinomorphic**	actinomorphic**	actinomorphic**	actinomorphic**
**Malvidae**	actinomorphic	actinomorphic**	actinomorphic**	actinomorphic*	actinomorphic*
**Fabidae**	actinomorphic	actinomorphic**	actinomorphic**	actinomorphic**	actinomorphic**
**Superasteridae**	actinomorphic	actinomorphic**	actinomorphic**	actinomorphic**	actinomorphic*
**Asteridae**	actinomorphic	actinomorphic**	actinomorphic**	actinomorphic**	actinomorphic*
**Campanulidae**	actinomorphic	actinomorphic**	actinomorphic**	actinomorphic*	actinomorphic*
**Lamiidae**	actinomorphic	actinomorphic**	actinomorphic**	actinomorphic	actinomorphic*

**RESCORED DATA (PrunedMB)**	**Parsimony**	**Maximum Likelihood (Mk1 model)**		**Maximum Likelihood (Mk2 model)**	
	**Both "full" and "part"**	**Mk1 Likelihood (full)**	**Mk1 Likelihood (part)**	**Mk2 Likelihood (full)**	**Mk2 Likelihood (part)**
**Angiospermae**	actinomorphic	actinomorphic**	actinomorphic**	actinomorphic*	actinomorphic (high rate)
**Mesangiospermae**	actinomorphic	actinomorphic**	?	actinomorphic**	?
**Magnoliidae**	actinomorphic	actinomorphic**	actinomorphic**	actinomorphic**	actinomorphic (high rate)
**Monocotyledoneae**	actinomorphic. zygomorphic	actinomorphic**	equivocal (high rate)	actinomorphic*	actinomorphic (high rate)
**Commelinidae**	actinomorphic	actinomorphic**	equivocal (high rate)	actinomorphic	actinomorphic (high rate)
**Eudicotyledoneae**	actinomorphic	**actinomorphic****	**equivocal**	actinomorphic**	**actinomorphic***
**Pentapetalae**	actinomorphic	actinomorphic**	?	actinomorphic**	?
**Superrosidae**	actinomorphic	actinomorphic**	actinomorphic**	actinomorphic**	actinomorphic**
**Rosidae**	actinomorphic	actinomorphic**	actinomorphic**	actinomorphic**	actinomorphic**
**Malvidae**	actinomorphic	actinomorphic**	actinomorphic**	actinomorphic**	actinomorphic**
**Fabidae**	actinomorphic	actinomorphic**	actinomorphic**	actinomorphic**	actinomorphic**
**Superasteridae**	actinomorphic	actinomorphic**	actinomorphic**	actinomorphic**	actinomorphic**
**Asteridae**	actinomorphic	actinomorphic**	actinomorphic**	actinomorphic**	actinomorphic**
**Campanulidae**	actinomorphic	actinomorphic**	actinomorphic**	actinomorphic**	actinomorphic**
**Lamiidae**	actinomorphic	actinomorphic**	actinomorphic**	actinomorphic*	actinomorphic*

Fusion
high rate= high rate equilibrium; full = full tree; part = partitioned tree; **state differences between comparable models**

RESCORED DATA (Five-char)	Parsimony	Maximum Likelihood (Mk1 model)		Maximum Likelihood (Mk2 model)	
	Both "full" and "part"	Mk1 Likelihood (full)	Mk1 Likelihood (part)	Mk2 Likelihood (full)	Mk2 Likelihood (part)
Angiospermae	free	free**	equivocal (high rate)	free**	free (high rate)
Mesangiospermae	free	free**	?	free**	?
Magnoliidae	free	free**	equivocal (high rate)	free**	free (high rate)
Monocotyledoneae	free	free**	free*	free**	free*
Commelinidae	free	free**	free*	free**	free
Eudicotyledoneae	free	free**	free**	free**	free*
Pentapetalae	free, fused	free*	?	free**	?
Superrosidae	fused	free*	equivocal (high rate)	free**	equivocal (high rate)
Rosidae	fused	free**	equivocal (high rate)	free**	equivocal (high rate)
Malvidae	free, fused	free*	equivocal (high rate)	free*	equivocal (high rate)
Fabidae	free	free**	equivocal (high rate)	free**	equivocal (high rate)
Superasteridae	free, fused	**free***	**fused****	**free***	**fused***
Asteridae	fused	fused*	fused**	fused	fused**
Campanulidae	fused	fused**	fused**	fused**	fused**
Lamiidae	fused	fused**	fused**	fused**	fused**

ORIGINAL DATA (Five-char)	Parsimony	Maximum Likelihood (Mk1 model)		Maximum Likelihood (Mk2 model)	
	Both "full" and "part"	Mk1 Likelihood (full)	Mk1 Likelihood (part)	Mk2 Likelihood (full)	Mk2 Likelihood (part)
Angiospermae	free	free**	free**	free**	free (high rate)
Mesangiospermae	free	free**	?	free**	?
Magnoliidae	free	free**	free**	free**	free (high rate)
Monocotyledoneae	free	free**	free*	free**	free*
Commelinidae	free	free**	free**	free**	free*
Eudicotyledoneae	free	free**	free**	free**	free**
Pentapetalae	free	free**	?	free**	?

**Superrosidae**	free	free**	free**	free**	free*
**Rosidae**	free	free**	free**	free**	free*
**Malvidae**	free	free**	free**	free**	free
**Fabidae**	free	free**	free**	free**	free*
**Superasteridae**	free, fused	**free****	**fused***	free**	**free****
**Asteridae**	free, fused	**free***	**fused***	free*	**free****
**Campanulidae**	fused	fused*	**fused****	**fused****	**free****
**Lamiidae**	fused	fused*	**fused****	**fused****	**free****

RESCORED DATA (PrunedMB)	Parsimony	Maximum Likelihood (Mk1 model)		Maximum Likelihood (Mk2 model)	
	Both "full" and "part"	Mk1 Likelihood (full)	Mk1 Likelihood (part)	Mk2 Likelihood (full)	Mk2 Likelihood (part)
**Angiospermae**	free	**free****	**equivocal**	free**	**free***
**Mesangiospermae**	free	free**	?	free**	?
**Magnoliidae**	free	**free****	**equivocal**	free**	**free***
**Monocotyledoneae**	free	free**	equivocal (high rate)	free**	free (high rate)
**Commelinidae**	free	free**	equivocal (high rate)	free**	free (high rate)
**Eudicotyledoneae**	free	free**	free**	free**	free*
**Pentapetalae**	free, fused	free**	?	free**	?
**Superrosidae**	free, fused	free**	free*	free**	free*
**Rosidae**	free, fused	free**	free*	free**	free*
**Malvidae**	free, fused	free*	free	free*	free
**Fabidae**	free	free**	free*	free**	free*
**Superasteridae**	free, fused	**free***	**fused****	**free****	**fused***
**Asteridae**	fused	fused*	fused**	fused*	fused**
**Campanulidae**	fused	fused**	fused**	fused**	fused**
**Lamiidae**	fused	fused**	fused**	fused**	fused**

Phyllotaxis

high rate= high rate equilibrium; full = full tree; part = partitioned tree; **state differences between comparable models**

RESCORED DATA (Five-char)	Parsimony	Maximum Likelihood (Mk1 model)		Maximum Likelihood (Mk2 model)	
	Both "full" and "part"	Mk1 Likelihood (full)	Mk1 Likelihood (part)	Mk2 Likelihood (full)	Mk2 Likelihood (part)

**Angiospermae**	whorled, spiral	**whorled****	whorled**	**spiral****	**whorled****
**Mesangiospermae**	whorled	**whorled****	?	**spiral****	?
**Magnoliidae**	whorled	**whorled****	whorled**	**spiral****	**whorled****
**Monocotyledoneae**	whorled	**whorled****	whorled**	**spiral***	**whorled****
**Commelinidae**	whorled	whorled**	whorled**	whorled**	whorled**
**Eudicotyledoneae**	whorled	**whorled****	whorled**	**spiral****	**whorled***
**Pentapetalae**	whorled	whorled**	?	whorled**	?
**Superrosidae**	whorled	whorled**	whorled**	whorled**	whorled**
**Rosidae**	whorled	whorled**	whorled**	whorled**	whorled**
**Malvidae**	whorled	whorled**	whorled**	whorled**	whorled**
**Fabidae**	whorled	whorled**	whorled**	whorled**	whorled**
**Superasteridae**	whorled	whorled**	whorled**	whorled**	whorled**
**Asteridae**	whorled	whorled**	whorled**	whorled**	whorled**
**Campanulidae**	whorled	whorled**	whorled**	whorled**	whorled**
**Lamiidae**	whorled	whorled**	whorled**	whorled**	whorled**

ORIGINAL DATA (Five-char)	Parsimony	Maximum Likelihood (Mk1 model)		Maximum Likelihood (Mk2 model)	
	Both "full" and "part"	Mk1 Likelihood (full)	Mk1 Likelihood (part)	Mk2 Likelihood (full)	Mk2 Likelihood (part)
**Angiospermae**	whorled, spiral	**whorled****	whorled**	**spiral****	whorled**
**Mesangiospermae**	whorled	**whorled****	?	**spiral****	?
**Magnoliidae**	whorled	**whorled****	whorled**	**spiral****	whorled**
**Monocotyledoneae**	whorled	**whorled****	whorled**	**spiral***	whorled**
**Commelinidae**	whorled	whorled**	whorled**	whorled**	whorled**
**Eudicotyledoneae**	whorled	**whorled****	whorled**	**spiral****	whorled*
**Pentapetalae**	whorled	whorled**	?	whorled**	?
**Superrosidae**	whorled	whorled**	whorled**	whorled**	whorled**
**Rosidae**	whorled	whorled**	whorled**	whorled**	whorled**
**Malvidae**	whorled	whorled**	whorled**	whorled**	whorled**
**Fabidae**	whorled	whorled**	whorled**	whorled**	whorled**
**Superasteridae**	whorled	whorled**	whorled**	whorled**	whorled**
**Asteridae**	whorled	whorled**	whorled**	whorled**	whorled**
**Campanulidae**	whorled	whorled**	whorled**	whorled**	whorled**
**Lamiidae**	whorled	whorled**	whorled**	whorled**	whorled**

RESCORED DATA (PrunedMB)	Parsimony	Maximum Likelihood (Mk1 model)		Maximum Likelihood (Mk2 model)	
	Both "full" and "part"	Mk1 Likelihood (full)	Mk1 Likelihood (part)	Mk2 Likelihood (full)	Mk2 Likelihood (part)
Angiospermae	whorled. spiral	**whorled****	whorled**	**spiral****	**whorled**
Mesangiospermae	whorled	**whorled****	?	**spiral****	?
Magnoliidae	whorled	**whorled****	whorled**	**spiral****	**whorled**
Monocotyledoneae	whorled	**whorled****	whorled**	**spiral***	**whorled****
Commelinidae	whorled	whorled**	whorled**	whorled**	whorled**
Eudicotyledoneae	whorled	**whorled****	whorled**	**spiral****	**whorled***
Pentapetalae	whorled	whorled**	?	whorled**	?
Superrosidae	whorled	whorled**	whorled**	whorled**	whorled**
Rosidae	whorled	whorled**	whorled**	whorled**	whorled**
Malvidae	whorled	whorled**	whorled**	whorled**	whorled**
Fabidae	whorled	whorled**	whorled**	whorled**	whorled**
Superasteridae	whorled	whorled**	whorled**	whorled**	whorled**
Asteridae	whorled	whorled**	whorled**	whorled**	whorled**
Campanulidae	whorled	whorled**	whorled**	whorled**	whorled**
Lamiidae	whorled	whorled**	whorled**	whorled**	whorled**

Merism

high rate= high rate equilibrium; full = full tree; part = partitioned tree;

RESCORED DATA (Five-char)	Parsimony	Maximum Likelihood (Mk1 model)		Maximum Likelihood (Mk2 model)	
	Both "full" and "part"	Mk1 Likelihood (full)	Mk1 Likelihood (part)	Mk2 Likelihood (full)	Mk2 Likelihood (part)
Angiospermae	non-penta	non-penta**	non-penta**	non-penta**	non-penta*
Mesangiospermae	non-penta	non-penta**	?	non-penta**	?
Magnoliidae	non-penta	non-penta**	non-penta**	non-penta**	non-penta*
Monocotyledoneae	non-penta	non-penta**	non-penta**	non-penta**	non-penta**
Commelinidae	non-penta	non-penta**	non-penta**	non-penta**	non-penta**
Eudicotyledoneae	non-penta	non-penta**	non-penta**	non-penta**	non-penta*
Pentapetalae	pentamerous	pentamerous**	?	pentamerous**	?
Superrosidae	pentamerous	pentamerous**	pentamerous**	pentamerous**	pentamerous
Rosidae	pentamerous	pentamerous**	pentamerous**	pentamerous**	pentamerous
Malvidae	pentamerous	pentamerous**	pentamerous**	pentamerous**	pentamerous
Fabidae	pentamerous	pentamerous**	pentamerous**	pentamerous**	pentamerous

**Superasteridae**	pentamerous	pentamerous**	pentamerous**	pentamerous**	pentamerous*
**Asteridae**	pentamerous	pentamerous**	pentamerous**	pentamerous**	pentamerous*
**Campanulidae**	pentamerous	pentamerous**	pentamerous**	pentamerous**	pentamerous*
**Lamiidae**	pentamerous	pentamerous**	pentamerous**	pentamerous**	pentamerous*

**ORIGINAL DATA (Five-char)**	**Parsimony**	**Maximum Likelihood (Mk1 model)**		**Maximum Likelihood (Mk2 model)**	
	**Both "full" and "part"**	**Mk1 Likelihood (full)**	**Mk1 Likelihood (part)**	**Mk2 Likelihood (full)**	**Mk2 Likelihood (part)**
**Angiospermae**	non-penta	non-penta**	non-penta**	non-penta**	non-penta**
**Mesangiospermae**	non-penta	non-penta**	?	non-penta**	?
**Magnoliidae**	non-penta	non-penta**	non-penta**	non-penta**	non-penta**
**Monocotyledoneae**	non-penta	non-penta**	non-penta**	non-penta**	non-penta**
**Commelinidae**	non-penta	non-penta**	non-penta**	non-penta**	non-penta**
**Eudicotyledoneae**	non-penta	non-penta**	non-penta**	non-penta**	non-penta**
**Pentapetalae**	non-penta, pentamerous	pentamerous*	?	pentamerous**	?
**Superrosidae**	pentamerous	pentamerous**	**pentamerous****	**pentamerous****	**non-penta**
**Rosidae**	pentamerous	pentamerous**	**pentamerous****	**pentamerous****	**non-penta**
**Malvidae**	pentamerous	pentamerous**	**pentamerous****	**pentamerous****	**non-penta**
**Fabidae**	pentamerous	pentamerous**	**pentamerous****	**pentamerous****	**non-penta**
**Superasteridae**	non-penta, pentamerous	pentamerous*	pentamerous*	pentamerous**	pentamerous*
**Asteridae**	pentamerous	pentamerous**	pentamerous**	pentamerous**	pentamerous
**Campanulidae**	pentamerous	pentamerous**	pentamerous**	pentamerous**	pentamerous*
**Lamiidae**	pentamerous	pentamerous**	pentamerous**	pentamerous**	pentamerous*

**RESCORED DATA (PrunedMB)**	**Parsimony**	**Maximum Likelihood (Mk1 model)**		**Maximum Likelihood (Mk2 model)**	
	**Both "full" and "part"**	**Mk1 Likelihood (full)**	**Mk1 Likelihood (part)**	**Mk2 Likelihood (full)**	**Mk2 Likelihood (part)**
**Angiospermae**	non-penta	non-penta**	non-penta**	non-penta**	non-penta*
**Mesangiospermae**	non-penta	non-penta**	?	non-penta**	?
**Magnoliidae**	non-penta	non-penta**	non-penta**	non-penta**	non-penta*
**Monocotyledoneae**	non-penta	non-penta**	non-penta**	non-penta**	non-penta**
**Commelinidae**	non-penta	non-penta**	non-penta**	non-penta**	non-penta**
**Eudicotyledoneae**	non-penta	non-penta**	non-penta**	non-penta**	non-penta*
**Pentapetalae**	pentamerous	pentamerous**	?	pentamerous**	?

**Superrosidae**	pentamerous	pentamerous**	pentamerous**	pentamerous**	pentamerous*
**Rosidae**	pentamerous	pentamerous**	pentamerous**	pentamerous**	pentamerous*
**Malvidae**	pentamerous	pentamerous**	pentamerous**	pentamerous**	pentamerous*
**Fabidae**	pentamerous	pentamerous**	pentamerous**	pentamerous**	pentamerous*
**Superasteridae**	pentamerous	pentamerous**	equivocal (high rate)	pentamerous**	pentamerous (high rate)
**Asteridae**	pentamerous	pentamerous**	equivocal (high rate)	pentamerous**	pentamerous (high rate)
**Campanulidae**	pentamerous	pentamerous**	equivocal (high rate)	pentamerous**	pentamerous (high rate)
**Lamiidae**	pentamerous	pentamerous**	equivocal (high rate)	pentamerous**	pentamerous (high rate)

Differentiation

high rate= high rate equilibrium; full = full tree; part = partitioned tree; **state differences between comparable models**

**RESCORED DATA (Five-char)**	**Parsimony**	**Maximum Likelihood (Mk1 model)**		**Maximum Likelihood (Mk2 model)**	
	**Both "full" and "part"**	**Mk1 Likelihood (full)**	**Mk1 Likelihood (part)**	**Mk2 Likelihood (full)**	**Mk2 Likelihood (part)**
**Angiospermae**	undifferentiated	**differentiated***	differentiated	**undifferentiated ****	**equivocal**
**Mesangiospermae**	undifferentiated	**differentiated***	?	**undifferentiated ****	?
**Magnoliidae**	undifferentiated	**differentiated***	differentiated	**undifferentiated ****	**equivocal**
**Monocotyledoneae**	undifferentiated	**differentiated***	**undifferentiated**	**undifferentiated ****	undifferentiated
**Commelinidae**	undifferentiated, differentiated	**differentiated****	differentiated	**undifferentiated ****	**differentiated**
**Eudicotyledoneae**	differentiated	**differentiated****	differentiated**	**undifferentiated ***	**differentiated****
**Pentapetalae**	differentiated	differentiated**	?	differentiated**	?
**Superrosidae**	differentiated	differentiated**	differentiated**	differentiated**	differentiated**
**Rosidae**	differentiated	differentiated**	differentiated**	differentiated**	differentiated**
**Malvidae**	differentiated	differentiated**	differentiated**	differentiated**	differentiated**
**Fabidae**	differentiated	differentiated**	differentiated**	differentiated**	differentiated**
**Superasteridae**	differentiated	differentiated**	differentiated**	differentiated**	differentiated**
**Asteridae**	differentiated	differentiated**	differentiated**	differentiated**	differentiated**
**Campanulidae**	differentiated	differentiated**	differentiated**	differentiated**	differentiated**
**Lamiidae**	differentiated	differentiated**	differentiated**	differentiated**	differentiated**

ORIGINAL DATA (Five-char)	Parsimony	Maximum Likelihood (Mk1 model)		Maximum Likelihood (Mk2 model)	
	Both "full" and "part"	Mk1 Likelihood (full)	Mk1 Likelihood (part)	Mk2 Likelihood (full)	Mk2 Likelihood (part)
Angiospermae	undifferentiated	**differentiated***	**undifferentiated**	**undifferentiated ****	undifferentiated
Mesangiospermae	undifferentiated	**differentiated***	?	**undifferentiated ****	?
Magnoliidae	undifferentiated	**differentiated***	**undifferentiated**	**undifferentiated ****	undifferentiated
Monocotyledoneae	undifferentiated	**differentiated***	**undifferentiated**	**undifferentiated ****	undifferentiated
Commelinidae	undifferentiated, differentiated	**differentiated***	differentiated	**undifferentiated ****	**differentiated**
Eudicotyledoneae	differentiated	**differentiated****	differentiated**	**undifferentiated ***	**differentiated****
Pentapetalae	differentiated	differentiated**	?	differentiated**	?
Superrosidae	differentiated	differentiated**	differentiated**	differentiated**	differentiated**
Rosidae	differentiated	differentiated**	differentiated**	differentiated**	differentiated**
Malvidae	differentiated	differentiated**	differentiated**	differentiated**	differentiated**
Fabidae	differentiated	differentiated**	differentiated**	differentiated**	differentiated**
Superasteridae	differentiated	differentiated**	differentiated**	differentiated**	differentiated**
Asteridae	differentiated	differentiated**	differentiated**	differentiated**	differentiated**
Campanulidae	differentiated	differentiated**	differentiated**	differentiated**	differentiated**
Lamiidae	differentiated	differentiated**	differentiated**	differentiated**	differentiated**

RESCORED DATA (PrunedMB)	Parsimony	Maximum Likelihood (Mk1 model)		Maximum Likelihood (Mk2 model)	
	Both "full" and "part"	Mk1 Likelihood (full)	Mk1 Likelihood (part)	Mk2 Likelihood (full)	Mk2 Likelihood (part)
Angiospermae	undifferentiated	**differentiated***	equivocal (high rate)	**undifferentiated ****	differentiated (high rate)
Mesangiospermae	undifferentiated	**differentiated****	?	**undifferentiated ****	?
Magnoliidae	undifferentiated	**differentiated****	equivocal (high rate)	**undifferentiated ****	differentiated (high rate)
Monocotyledoneae	undifferentiated	**differentiated***	**undifferentiated**	**undifferentiated ****	undifferentiated
Commelinidae	differentiated	**differentiated****	differentiated*	**undifferentiated ****	**differentiated**
Eudicotyledoneae	differentiated	**differentiated****	differentiated**	**undifferentiated ***	**differentiated****
Pentapetalae	differentiated	differentiated**	?	differentiated**	?

**Superrosidae**	differentiated	differentiated**	differentiated**	differentiated**	differentiated**
**Rosidae**	differentiated	differentiated**	differentiated**	differentiated**	differentiated**
**Malvidae**	differentiated	differentiated**	differentiated**	differentiated**	differentiated**
**Fabidae**	differentiated	differentiated**	differentiated**	differentiated**	differentiated**
**Superasteridae**	differentiated	differentiated**	differentiated**	differentiated**	differentiated*
**Asteridae**	differentiated	differentiated**	differentiated**	differentiated**	differentiated**
**Campanulidae**	differentiated	differentiated**	differentiated**	differentiated**	differentiated**
**Lamiidae**	differentiated	differentiated**	differentiated**	differentiated**	differentiated**

Testing the impact of morphological rate heterogeneity on ancestral state reconstruction of five floral traits in angiosperms

Supplementary Tables 3: Parsimony, Mk1 and Mk2 ancestral states for the crown-group nodes of 54 botanical orders

Elisabeth Reyes, Sophie Nadot, Maria von Balthazar, Jürg Schönenberger, Hervé Sauquet

Supplementary Tables 3: Parsimony, Mk1 and Mk2 ancestral states for the crown-group nodes of 54 botanical orders

Five-char: Results for main tree
PrunedMB: Results for the pruned Smith & Brown ALLMB tree
Asterisks in the maximum likelihood columns: none = weak support (0.51-0.60), one = moderate support (0.61-0.89), two = strong support (0.90-1)

Symmetry

high rate= high rate equilibrium; full = full tree; part = partitioned tree; **state differences between comparable models**

RESCORED DATA (Five-char)	Parsimony	Maximum Likelihood (Mk1 model)		Maximum Likelihood (Mk2 model)	
	Both "full" and "part"	Mk1 Likelihood (full)	Mk1 Likelihood (part)	Mk2 Likelihood (full)	Mk2 Likelihood (part)
**Alismatales**	actinomorphic	actinomorphic**	equivocal (high rate)	actinomorphic*	actinomorphic (high rate)
**Apiales**	actinomorphic	actinomorphic**	actinomorphic**	actinomorphic*	actinomorphic*
**Aquifoliales**	actinomorphic	actinomorphic**	actinomorphic**	actinomorphic*	actinomorphic*
**Arecales**	actinomorphic	actinomorphic**	equivocal (high rate)	actinomorphic*	actinomorphic (high rate)
**Asparagales**	actinomorphic	**actinomorphic****	equivocal (high rate)	**equivocal**	actinomorphic (high rate)
**Asterales**	actinomorphic	actinomorphic**	actinomorphic**	actinomorphic	actinomorphic*
**Austrobaileyales**	actinomorphic	actinomorphic**	actinomorphic**	actinomorphic*	actinomorphic**
**Berberidopsidales**	actinomorphic	actinomorphic**	actinomorphic**	actinomorphic**	actinomorphic**
**Boraginales**	actinomorphic	actinomorphic**	actinomorphic**	actinomorphic	actinomorphic*
**Brassicales**	actinomorphic	actinomorphic**	**actinomorphic****	**actinomorphic**	**zygomorphic**
**Bruniales**	actinomorphic	actinomorphic**	actinomorphic**	actinomorphic*	actinomorphic*
**Buxales**	actinomorphic	actinomorphic**	equivocal (high rate)	actinomorphic*	actinomorphic*
**Canellales**	actinomorphic	actinomorphic**	actinomorphic**	actinomorphic*	actinomorphic**
**Caryophyllales**	actinomorphic	actinomorphic**	actinomorphic**	actinomorphic**	actinomorphic*
**Celastrales**	actinomorphic	actinomorphic**	actinomorphic**	actinomorphic*	actinomorphic**
**Commelinales**	actinomorphic, zygomorphic	zygomorphic*	equivocal (high rate)	zygomorphic**	actinomorphic (high rate)
**Cornales**	actinomorphic	actinomorphic**	actinomorphic**	actinomorphic*	actinomorphic*
**Crossosomatales**	actinomorphic	actinomorphic**	actinomorphic**	actinomorphic*	actinomorphic**
**Cucurbitales**	actinomorphic	actinomorphic**	actinomorphic**	actinomorphic**	actinomorphic**
**Dioscoreales**	actinomorphic	actinomorphic**	equivocal (high rate)	actinomorphic*	actinomorphic (high rate)
**Dipsacales**	actinomorphic	actinomorphic*	actinomorphic*	actinomorphic	actinomorphic
**Ericales**	actinomorphic	actinomorphic**	actinomorphic**	actinomorphic*	actinomorphic*

**Escalloniales**	actinomorphic	actinomorphic**	actinomorphic**	actinomorphic*	actinomorphic*
**Fabales**	actinomorphic	actinomorphic*	actinomorphic*	actinomorphic*	actinomorphic
**Fagales**	actinomorphic	actinomorphic**	actinomorphic**	actinomorphic*	actinomorphic**
**Garryales**	actinomorphic	actinomorphic**	actinomorphic**	actinomorphic**	actinomorphic**
**Gentianales**	actinomorphic	actinomorphic**	actinomorphic**	actinomorphic*	actinomorphic*
**Geraniales**	actinomorphic	actinomorphic**	actinomorphic**	actinomorphic*	actinomorphic*
**Huerteales**	actinomorphic	actinomorphic**	actinomorphic**	actinomorphic**	actinomorphic**
**Icacinales**	actinomorphic	actinomorphic**	actinomorphic**	actinomorphic*	actinomorphic*
**Lamiales**	actinomorphic, zygomorphic	**actinomorphic***	**actinomorphic***	**zygomorphic***	**zygomorphic***
**Laurales**	actinomorphic	actinomorphic**	actinomorphic**	actinomorphic*	actinomorphic**
**Liliales**	actinomorphic	actinomorphic**	equivocal (high rate)	actinomorphic	actinomorphic (high rate)
**Magnoliales**	actinomorphic	actinomorphic**	actinomorphic**	actinomorphic*	actinomorphic**
**Malpighiales**	actinomorphic	actinomorphic**	actinomorphic**	actinomorphic**	actinomorphic**
**Malvales**	actinomorphic	actinomorphic**	actinomorphic**	actinomorphic*	actinomorphic*
**Myrtales**	actinomorphic	actinomorphic**	actinomorphic**	actinomorphic*	actinomorphic*
**Nymphaeales**	actinomorphic	actinomorphic**	actinomorphic**	actinomorphic*	actinomorphic**
**Oxalidales**	actinomorphic	actinomorphic**	actinomorphic**	actinomorphic**	actinomorphic**
**Pandanales**	actinomorphic	actinomorphic**	equivocal (high rate)	actinomorphic*	actinomorphic (high rate)
**Paracryphiales**	actinomorphic	actinomorphic**	actinomorphic**	actinomorphic*	actinomorphic*
**Petrosaviales**	actinomorphic	actinomorphic**	equivocal (high rate)	actinomorphic*	actinomorphic (high rate)
**Piperales**	actinomorphic	actinomorphic**	actinomorphic**	actinomorphic*	actinomorphic**
**Poales**	actinomorphic	**actinomorphic****	equivocal (high rate)	**zygomorphic**	actinomorphic (high rate)
**Proteales**	actinomorphic	actinomorphic**	equivocal (high rate)	actinomorphic*	actinomorphic*
**Ranunculales**	actinomorphic	actinomorphic**	equivocal (high rate)	actinomorphic*	actinomorphic*
**Rosales**	actinomorphic	actinomorphic**	actinomorphic**	actinomorphic**	actinomorphic**
**Santalales**	actinomorphic	actinomorphic**	actinomorphic**	actinomorphic*	actinomorphic*
**Sapindales**	actinomorphic	actinomorphic**	actinomorphic**	actinomorphic*	actinomorphic**
**Saxifragales**	actinomorphic	actinomorphic**	actinomorphic**	actinomorphic**	actinomorphic**
**Solanales**	actinomorphic	**actinomorphic****	actinomorphic**	**zygomorphic**	actinomorphic
**Vitales**	actinomorphic	actinomorphic**	actinomorphic**	actinomorphic*	actinomorphic**
**Zingiberales**	zygomorphic	zygomorphic**	equivocal (high rate)	zygomorphic**	actinomorphic (high rate)

**Zygophyllales**	actinomorphic	actinomorphic*	actinomorphic*	actinomorphic*	actinomorphic*

**ORIGINAL DATA (Five-char)**	**Parsimony**	**Maximum Likelihood (Mk1 model)**		**Maximum Likelihood (Mk2 model)**	
	**Both "full" and "part"**	**Mk1 Likelihood (full)**	**Mk1 Likelihood (part)**	**Mk2 Likelihood (full)**	**Mk2 Likelihood (part)**
**Alismatales**	actinomorphic	actinomorphic**	equivocal (high rate)	actinomorphic*	actinomorphic (high rate)
**Apiales**	actinomorphic	actinomorphic**	actinomorphic**	actinomorphic*	actinomorphic*
**Aquifoliales**	actinomorphic	actinomorphic**	actinomorphic**	actinomorphic*	actinomorphic*
**Arecales**	actinomorphic	actinomorphic**	equivocal (high rate)	actinomorphic*	actinomorphic (high rate)
**Asparagales**	actinomorphic	**actinomorphic****	equivocal (high rate)	**zygomorphic**	actinomorphic (high rate)
**Asterales**	actinomorphic	actinomorphic**	actinomorphic**	actinomorphic*	actinomorphic*
**Austrobaileyales**	actinomorphic	actinomorphic**	actinomorphic**	actinomorphic*	actinomorphic**
**Berberidopsidales**	actinomorphic	actinomorphic**	actinomorphic**	actinomorphic**	actinomorphic**
**Boraginales**	actinomorphic	**actinomorphic****	actinomorphic**	**equivocal**	**actinomorphic**
**Brassicales**	actinomorphic	actinomorphic**	actinomorphic**	actinomorphic	zygomorphic
**Bruniales**	actinomorphic	actinomorphic**	actinomorphic**	actinomorphic*	actinomorphic*
**Buxales**	actinomorphic	actinomorphic**	actinomorphic	actinomorphic*	actinomorphic*
**Canellales**	actinomorphic	actinomorphic**	actinomorphic**	actinomorphic*	actinomorphic (high rate)
**Caryophyllales**	actinomorphic	actinomorphic**	actinomorphic**	actinomorphic**	actinomorphic**
**Celastrales**	actinomorphic	actinomorphic**	actinomorphic**	actinomorphic**	actinomorphic**
**Commelinales**	actinomorphic, zygomorphic	zygomorphic	equivocal (high rate)	zygomorphic**	actinomorphic (high rate)
**Cornales**	actinomorphic	actinomorphic**	actinomorphic**	actinomorphic**	actinomorphic*
**Crossosomatales**	actinomorphic	actinomorphic**	actinomorphic**	actinomorphic**	actinomorphic**
**Cucurbitales**	actinomorphic	actinomorphic**	actinomorphic**	actinomorphic**	actinomorphic**
**Dioscoreales**	actinomorphic	actinomorphic**	equivocal (high rate)	actinomorphic*	actinomorphic (high rate)
**Dipsacales**	actinomorphic	actinomorphic*	actinomorphic*	actinomorphic*	actinomorphic*
**Ericales**	actinomorphic	actinomorphic**	actinomorphic**	actinomorphic**	actinomorphic**
**Escalloniales**	actinomorphic	actinomorphic**	actinomorphic**	actinomorphic*	actinomorphic*
**Fabales**	actinomorphic	actinomorphic*	actinomorphic*	actinomorphic*	actinomorphic
**Fagales**	actinomorphic	actinomorphic**	actinomorphic**	actinomorphic**	actinomorphic**
**Garryales**	actinomorphic	actinomorphic**	actinomorphic**	actinomorphic**	actinomorphic**
**Gentianales**	actinomorphic	actinomorphic**	actinomorphic**	actinomorphic*	actinomorphic*

**Geraniales**	actinomorphic	actinomorphic**	actinomorphic**	actinomorphic*	actinomorphic*
**Huerteales**	actinomorphic	actinomorphic**	actinomorphic**	actinomorphic**	actinomorphic**
**Icacinales**	actinomorphic	actinomorphic**	actinomorphic**	actinomorphic*	actinomorphic*
**Lamiales**	actinomorphic, zygomorphic	**actinomorphic***	**actinomorphic***	**zygomorphic***	**zygomorphic***
**Laurales**	actinomorphic	actinomorphic**	actinomorphic**	actinomorphic**	actinomorphic (high rate)
**Liliales**	actinomorphic	actinomorphic**	equivocal (high rate)	actinomorphic	actinomorphic (high rate)
**Magnoliales**	actinomorphic	actinomorphic**	actinomorphic**	actinomorphic**	actinomorphic (high rate)
**Malpighiales**	actinomorphic	actinomorphic**	actinomorphic**	actinomorphic**	actinomorphic**
**Malvales**	actinomorphic	actinomorphic**	actinomorphic**	actinomorphic*	actinomorphic**
**Myrtales**	actinomorphic	actinomorphic**	actinomorphic**	actinomorphic*	actinomorphic**
**Nymphaeales**	actinomorphic	actinomorphic**	actinomorphic**	actinomorphic*	actinomorphic (high rate)
**Oxalidales**	actinomorphic	actinomorphic**	actinomorphic**	actinomorphic**	actinomorphic**
**Pandanales**	actinomorphic	actinomorphic**	equivocal (high rate)	actinomorphic*	actinomorphic (high rate)
**Paracryphiales**	actinomorphic	actinomorphic**	actinomorphic**	actinomorphic*	actinomorphic*
**Petrosaviales**	actinomorphic	actinomorphic**	equivocal (high rate)	actinomorphic*	actinomorphic (high rate)
**Piperales**	actinomorphic	actinomorphic**	actinomorphic**	actinomorphic*	actinomorphic (high rate)
**Poales**	actinomorphic	**actinomorphic****	equivocal (high rate)	**zygomorphic***	actinomorphic (high rate)
**Proteales**	actinomorphic	actinomorphic**	actinomorphic	actinomorphic*	actinomorphic*
**Ranunculales**	actinomorphic	actinomorphic**	actinomorphic	actinomorphic*	actinomorphic*
**Rosales**	actinomorphic	actinomorphic**	actinomorphic**	actinomorphic**	actinomorphic**
**Santalales**	actinomorphic	actinomorphic**	actinomorphic**	actinomorphic*	actinomorphic*
**Sapindales**	actinomorphic	actinomorphic**	actinomorphic**	actinomorphic*	actinomorphic**
**Saxifragales**	actinomorphic	actinomorphic**	actinomorphic**	actinomorphic**	actinomorphic**
**Solanales**	actinomorphic	**actinomorphic****	actinomorphic**	**zygomorphic**	actinomorphic
**Vitales**	actinomorphic	actinomorphic**	actinomorphic**	actinomorphic*	actinomorphic**
**Zingiberales**	zygomorphic	zygomorphic**	equivocal (high rate)	zygomorphic**	actinomorphic (high rate)
**Zygophyllales**	actinomorphic	actinomorphic*	actinomorphic*	actinomorphic*	actinomorphic*

**RESCORED DATA (PrunedMB)**	**Parsimony**	**Maximum Likelihood (Mk1 model)**		**Maximum Likelihood (Mk2 model)**	

	Both "full" and "part"	Mk1 Likelihood (full)	Mk1 Likelihood (part)	Mk2 Likelihood (full)	Mk2 Likelihood (part)
**Alismatales**	actinomorphic	actinomorphic**	equivocal (high rate)	actinomorphic*	actinomorphic (high rate)
**Apiales**	actinomorphic	actinomorphic**	actinomorphic**	actinomorphic*	actinomorphic*
**Aquifoliales**	actinomorphic	actinomorphic**	actinomorphic**	actinomorphic**	actinomorphic**
**Arecales**	actinomorphic	**actinomorphic****	equivocal (high rate)	**equivocal**	actinomorphic (high rate)
**Asparagales**	actinomorphic	actinomorphic**	equivocal (high rate)	actinomorphic*	actinomorphic (high rate)
**Asterales**	actinomorphic	actinomorphic**	actinomorphic**	actinomorphic*	actinomorphic*
**Austrobaileyales**	actinomorphic	actinomorphic**	actinomorphic**	actinomorphic*	actinomorphic (high rate)
**Berberidopsidales**	actinomorphic	actinomorphic**	actinomorphic**	actinomorphic**	actinomorphic**
**Boraginales**	actinomorphic	actinomorphic**	actinomorphic**	actinomorphic*	actinomorphic*
**Brassicales**	actinomorphic	actinomorphic**	actinomorphic**	actinomorphic*	actinomorphic*
**Bruniales**	actinomorphic	actinomorphic**	actinomorphic**	actinomorphic*	actinomorphic*
**Buxales**	actinomorphic	**actinomorphic****	**equivocal**	actinomorphic*	**actinomorphic***
**Canellales**	actinomorphic	actinomorphic**	actinomorphic**	actinomorphic*	actinomorphic (high rate)
**Caryophyllales**	actinomorphic	actinomorphic**	actinomorphic**	actinomorphic**	actinomorphic**
**Celastrales**	actinomorphic	actinomorphic**	actinomorphic**	actinomorphic*	actinomorphic**
**Commelinales**	actinomorphic, zygomorphic	zygomorphic	equivocal (high rate)	zygomorphic*	actinomorphic (high rate)
**Cornales**	actinomorphic	actinomorphic**	actinomorphic**	actinomorphic**	actinomorphic**
**Crossosomatales**	actinomorphic	actinomorphic**	actinomorphic**	actinomorphic*	actinomorphic**
**Cucurbitales**	actinomorphic	actinomorphic**	actinomorphic**	actinomorphic*	actinomorphic**
**Dioscoreales**	actinomorphic	actinomorphic**	equivocal (high rate)	actinomorphic*	actinomorphic (high rate)
**Dipsacales**	actinomorphic	actinomorphic*	actinomorphic*	actinomorphic*	actinomorphic*
**Ericales**	actinomorphic	actinomorphic**	actinomorphic**	actinomorphic**	actinomorphic**
**Escalloniales**	actinomorphic	actinomorphic**	actinomorphic**	actinomorphic*	actinomorphic*
**Fabales**	actinomorphic	actinomorphic*	actinomorphic*	actinomorphic*	actinomorphic*
**Fagales**	actinomorphic	actinomorphic**	actinomorphic**	actinomorphic**	actinomorphic**
**Garryales**	actinomorphic	actinomorphic**	actinomorphic**	actinomorphic**	actinomorphic**
**Gentianales**	actinomorphic	actinomorphic**	actinomorphic**	actinomorphic*	actinomorphic*
**Geraniales**	actinomorphic	actinomorphic**	actinomorphic**	actinomorphic*	actinomorphic*
**Huerteales**	actinomorphic	actinomorphic**	actinomorphic**	actinomorphic*	actinomorphic**
**Icacinales**	actinomorphic	actinomorphic**	actinomorphic**	actinomorphic**	actinomorphic**

**Lamiales**	actinomorphic	**actinomorphic***	actinomorphic*	**zygomorphic**	**actinomorphic**
**Laurales**	actinomorphic	actinomorphic**	actinomorphic**	actinomorphic**	actinomorphic (high rate)
**Liliales**	actinomorphic	actinomorphic**	equivocal (high rate)	actinomorphic*	actinomorphic (high rate)
**Magnoliales**	actinomorphic	actinomorphic**	actinomorphic**	actinomorphic**	actinomorphic (high rate)
**Malpighiales**	actinomorphic	actinomorphic**	actinomorphic**	actinomorphic**	actinomorphic**
**Malvales**	actinomorphic	actinomorphic**	actinomorphic**	actinomorphic*	actinomorphic**
**Myrtales**	actinomorphic	actinomorphic**	actinomorphic**	actinomorphic**	actinomorphic**
**Nymphaeales**	actinomorphic	actinomorphic**	actinomorphic**	actinomorphic*	actinomorphic (high rate)
**Oxalidales**	actinomorphic	actinomorphic**	actinomorphic**	actinomorphic**	actinomorphic**
**Pandanales**	actinomorphic	actinomorphic**	equivocal (high rate)	actinomorphic*	actinomorphic (high rate)
**Paracryphiales**	actinomorphic	actinomorphic**	actinomorphic**	actinomorphic*	actinomorphic*
**Petrosaviales**	actinomorphic	actinomorphic**	equivocal (high rate)	actinomorphic*	actinomorphic (high rate)
**Piperales**	actinomorphic	actinomorphic**	actinomorphic**	actinomorphic*	actinomorphic (high rate)
**Poales**	actinomorphic	actinomorphic**	equivocal (high rate)	actinomorphic	actinomorphic (high rate)
**Proteales**	actinomorphic	**actinomorphic****	**equivocal**	actinomorphic**	**actinomorphic***
**Ranunculales**	actinomorphic	**actinomorphic****	**equivocal**	actinomorphic**	**actinomorphic***
**Rosales**	actinomorphic	actinomorphic**	actinomorphic**	actinomorphic**	actinomorphic**
**Santalales**	actinomorphic	actinomorphic**	actinomorphic**	actinomorphic**	actinomorphic**
**Sapindales**	actinomorphic	actinomorphic**	actinomorphic**	actinomorphic*	actinomorphic**
**Saxifragales**	actinomorphic	actinomorphic**	actinomorphic**	actinomorphic**	actinomorphic**
**Solanales**	actinomorphic	actinomorphic**	actinomorphic**	actinomorphic*	actinomorphic*
**Vitales**	actinomorphic	actinomorphic**	actinomorphic**	actinomorphic*	actinomorphic*
**Zingiberales**	zygomorphic	zygomorphic**	equivocal (high rate)	zygomorphic*	actinomorphic (high rate)
**Zygophyllales**	actinomorphic	actinomorphic**	actinomorphic**	actinomorphic*	actinomorphic*

Fusion

high rate= high rate equilibrium; full = full tree; part = partitioned tree; **state differences between comparable models**

**RESCORED DATA (Five-char)**	**Parsimony**	**Maximum Likelihood (Mk1 model)**		**Maximum Likelihood (Mk2 model)**	
	**Both "full" and "part"**	**Mk1 Likelihood (full)**	**Mk1 Likelihood (part)**	**Mk2 Likelihood (full)**	**Mk2 Likelihood (part)**

**Alismatales**	free	free**	free*	free**	free*
**Apiales**	free, fused	fused**	fused**	fused**	fused*
**Aquifoliales**	fused	fused**	fused**	fused*	fused**
**Arecales**	free	free*	free*	free*	free*
**Asparagales**	free	free**	free*	free**	free*
**Asterales**	fused	fused**	fused**	fused**	fused**
**Austrobaileyales**	free	free**	equivocal (high rate)	free**	free (high rate)
**Berberidopsidales**	free	free**	free**	free**	free**
**Boraginales**	fused	fused**	fused**	fused**	fused**
**Brassicales**	free, fused	fused*	equivocal (high rate)	fused	equivocal (high rate)
**Bruniales**	free, fused	fused**	fused**	fused**	fused**
**Buxales**	free	free*	free*	free*	free*
**Canellales**	free, fused	free**	equivocal (high rate)	free**	free (high rate)
**Caryophyllales**	fused	**free***	**fused***	**free***	**fused***
**Celastrales**	free	free**	equivocal (high rate)	free**	equivocal (high rate)
**Commelinales**	free, fused	free*	free*	free*	free*
**Cornales**	fused	fused*	fused**	fused	fused*
**Crossosomatales**	fused	free*	equivocal (high rate)	free*	equivocal (high rate)
**Cucurbitales**	free	free*	equivocal (high rate)	free*	equivocal (high rate)
**Dioscoreales**	free	free*	free	free*	free
**Dipsacales**	fused	fused**	fused**	fused**	fused**
**Ericales**	fused	fused**	fused**	fused**	fused**
**Escalloniales**	fused	fused**	fused**	fused**	fused**
**Fabales**	free	free*	equivocal (high rate)	free*	equivocal (high rate)
**Fagales**	free, fused	free*	equivocal (high rate)	free**	equivocal (high rate)
**Garryales**	free, fused	free*	free*	free*	free*
**Gentianales**	fused	fused**	fused**	fused**	fused**
**Geraniales**	fused	free**	equivocal (high rate)	free**	equivocal (high rate)
**Huerteales**	free, fused	free*	equivocal (high rate)	free*	equivocal (high rate)
**Icacinales**	fused	fused**	fused**	fused*	fused**

**Lamiales**	fused	fused**	fused**	fused**	fused**
**Laurales**	free	free**	equivocal (high rate)	free**	free (high rate)
**Liliales**	free	free**	free*	free**	free*
**Magnoliales**	free	free**	equivocal (high rate)	free**	free (high rate)
**Malpighiales**	free	free**	equivocal (high rate)	free**	equivocal (high rate)
**Malvales**	free, fused	fused*	equivocal (high rate)	fused*	equivocal (high rate)
**Myrtales**	fused	free*	equivocal (high rate)	free*	equivocal (high rate)
**Nymphaeales**	free	free*	equivocal (high rate)	free*	free (high rate)
**Oxalidales**	free	free*	equivocal (high rate)	free**	equivocal (high rate)
**Pandanales**	free	free*	free*	free*	free*
**Paracryphiales**	free	free	free*	free	free*
**Petrosaviales**	free	free*	free*	free*	free*
**Piperales**	free, fused	free*	equivocal (high rate)	free*	free (high rate)
**Poales**	free, fused	free**	free*	free**	free
**Proteales**	free	free**	free**	free**	free*
**Ranunculales**	free	free**	free**	free**	free*
**Rosales**	free	free*	equivocal (high rate)	free**	equivocal (high rate)
**Santalales**	fused	fused*	fused**	fused*	fused**
**Sapindales**	free, fused	free*	equivocal (high rate)	free*	equivocal (high rate)
**Saxifragales**	fused	free*	equivocal (high rate)	free*	equivocal (high rate)
**Solanales**	fused	fused**	fused**	fused**	fused**
**Vitales**	fused	fused*	equivocal (high rate)	fused*	equivocal (high rate)
**Zingiberales**	fused	fused**	fused*	fused**	fused*
**Zygophyllales**	free	free**	equivocal (high rate)	free**	free (high rate)

**ORIGINAL DATA (Five-char)**	**Parsimony**	**Maximum Likelihood (Mk1 model)**		**Maximum Likelihood (Mk2 model)**	
	**Both "full" and "part"**	**Mk1 Likelihood (full)**	**Mk1 Likelihood (part)**	**Mk2 Likelihood (full)**	**Mk2 Likelihood (part)**
**Alismatales**	free	free**	free**	free**	free*

**Apiales**	free	**free**	free	**fused**	**free****
**Aquifoliales**	fused	fused*	**fused****	**fused****	**free****
**Arecales**	free	free**	free*	free**	free*
**Asparagales**	free	free**	free**	free**	free*
**Asterales**	fused	fused**	fused**	fused**	fused
**Austrobaileyales**	free	free**	free**	free**	free<br>(high rate)
**Berberidopsidales**	free	free**	free**	free**	free**
**Boraginales**	fused	fused**	fused**	fused**	fused**
**Brassicales**	free	free**	free*	free**	free*
**Bruniales**	free, fused	fused*	**fused***	**fused***	**free****
**Buxales**	free	free**	free**	free**	free**
**Canellales**	free	free**	free**	free**	free<br>(high rate)
**Caryophyllales**	free, fused	**free****	**fused**	free*	**free****
**Celastrales**	free	free**	free**	free**	free*
**Commelinales**	free	free**	free**	free**	free*
**Cornales**	free	free*	free	free*	free**
**Crossosomatales**	free, fused	free*	free*	free*	free*
**Cucurbitales**	free	free*	free*	free*	free
**Dioscoreales**	free	free*	free*	free*	free
**Dipsacales**	fused	fused*	fused**	fused**	fused
**Ericales**	free, fused	fused*	**fused****	**fused***	**free****
**Escalloniales**	fused	fused*	fused**	fused**	free*
**Fabales**	free	free**	free*	free**	free*
**Fagales**	free	free**	free**	free**	free*
**Garryales**	free, fused	free*	free**	free*	free**
**Gentianales**	fused	fused**	fused**	fused**	fused**
**Geraniales**	free, fused	free**	free**	free**	free*
**Huerteales**	free	free**	free*	free**	free*
**Icacinales**	fused	fused*	fused**	fused**	free
**Lamiales**	fused	fused**	fused**	fused*	fused**
**Laurales**	free	free**	free**	free**	free<br>(high rate)
**Liliales**	free	free**	free**	free**	free*
**Magnoliales**	free	free**	free**	free**	free<br>(high rate)

**Malpighiales**	free	free**	free**	free**	free*
**Malvales**	free, fused	fused*	**fused**	**fused***	**free**
**Myrtales**	free, fused	free**	free*	free*	free*
**Nymphaeales**	free	free**	free**	free**	free<br>(high rate)
**Oxalidales**	free	free**	free**	free**	free*
**Pandanales**	free	free*	free*	free*	free*
**Paracryphiales**	free	free*	free*	free*	free**
**Petrosaviales**	free	free*	free*	free*	free*
**Piperales**	free, fused	free**	free**	free**	free<br>(high rate)
**Poales**	free	free**	free**	free**	free*
**Proteales**	free	free**	free**	free**	free**
**Ranunculales**	free	free**	free**	free**	free**
**Rosales**	free	free**	free**	free**	free*
**Santalales**	fused	fused*	**fused****	**fused***	**free***
**Sapindales**	free	free**	free**	free**	free
**Saxifragales**	free, fused	free**	free*	free*	free*
**Solanales**	fused	fused**	fused**	fused**	fused**
**Vitales**	free, fused	**fused**	**fused**	**equivocal**	**free**
**Zingiberales**	free, fused	fused	fused	fused	fused
**Zygophyllales**	free	free**	free**	free**	free**

**RESCORED DATA (PrunedMB)**	**Parsimony**	**Maximum Likelihood (Mk1 model)**		**Maximum Likelihood (Mk2 model)**	
	**Both "full" and "part"**	**Mk1 Likelihood (full)**	**Mk1 Likelihood (part)**	**Mk2 Likelihood (full)**	**Mk2 Likelihood (part)**
**Alismatales**	free	free**	equivocal<br>(high rate)	free**	free (high rate)
**Apiales**	free, fused	fused**	fused**	fused**	fused*
**Aquifoliales**	fused	fused*	fused**	fused*	fused*
**Arecales**	free	free*	equivocal<br>(high rate)	free**	free (high rate)
**Asparagales**	free	free**	equivocal	free**	free (high rate)
**Asterales**	fused	fused**	fused**	fused**	fused**
**Austrobaileyales**	free	free*	equivocal	free*	free
**Berberidopsidales**	free	free**	free**	free**	free**
**Boraginales**	fused	fused**	fused**	fused**	fused**

**Brassicales**	fused	**fused***	fused	**free**	**fused**
**Bruniales**	free, fused	fused**	fused**	fused*	fused**
**Buxales**	free	free*	free**	free**	free*
**Canellales**	fused	**free***	**equivocal**	free**	**free***
**Caryophyllales**	fused	**free***	**fused***	free*	**free**
**Celastrales**	free	free*	free	free*	free
**Commelinales**	free	free**	equivocal (high rate)	free**	free (high rate)
**Cornales**	fused	fused*	fused**	fused*	fused*
**Crossosomatales**	free, fused	free*	free	free*	free
**Cucurbitales**	free	**free***	**fused**	**free***	**fused**
**Dioscoreales**	free	free*	equivocal (high rate)	free*	free (high rate)
**Dipsacales**	fused	fused**	fused**	fused**	fused**
**Ericales**	fused	fused**	fused**	fused**	fused**
**Escalloniales**	fused	fused**	fused**	fused**	fused**
**Fabales**	free	free*	free	free*	free
**Fagales**	free, fused	free**	free	free**	free
**Garryales**	free, fused	free*	free**	free*	free*
**Gentianales**	fused	fused**	fused**	fused**	fused**
**Geraniales**	free, fused	free**	free*	free**	free*
**Huerteales**	free, fused	free*	free**	free*	free*
**Icacinales**	fused	fused**	fused**	fused**	fused**
**Lamiales**	fused	fused**	fused**	fused**	fused**
**Laurales**	free	**free****	**equivocal**	free**	**free***
**Liliales**	free	free**	equivocal (high rate)	free**	free (high rate)
**Magnoliales**	free	free**	free	free**	free*
**Malpighiales**	fused	free**	free*	free**	free*
**Malvales**	fused	fused*	fused*	fused*	fused*
**Myrtales**	free, fused	free*	free	free**	free
**Nymphaeales**	free	**free***	**equivocal**	free*	**free***
**Oxalidales**	free	free*	free*	free**	free*
**Pandanales**	free	free*	equivocal (high rate)	free*	free (high rate)
**Paracryphiales**	free	free	free*	free	free*

**Petrosaviales**	free	free*	equivocal (high rate)	free*	free (high rate)
**Piperales**	free, fused	**free***	**equivocal**	free*	**free***
**Poales**	free, fused	free**	equivocal (high rate)	free**	free (high rate)
**Proteales**	free	free**	free**	free**	free*
**Ranunculales**	free	free**	free**	free**	free*
**Rosales**	free	free**	free	free**	free
**Santalales**	fused	fused*	fused**	fused*	fused**
**Sapindales**	free, fused	**free**	**equivocal**	**free***	**fused**
**Saxifragales**	free, fused	free**	free*	free**	free*
**Solanales**	fused	fused**	fused**	fused**	fused**
**Vitales**	fused	fused*	fused	fused*	fused
**Zingiberales**	fused	fused*	equivocal (high rate)	fused*	free (high rate)
**Zygophyllales**	free	free**	free*	free**	free*

Phyllotaxis

high rate= high rate equilibrium; full = full tree; part = partitioned tree; **state differences between comparable models**

**RESCORED DATA (Five-char)**	**Parsimony**	**Maximum Likelihood (Mk1 model)**		**Maximum Likelihood (Mk2 model)**	
	**Both "full" and "part"**	**Mk1 Likelihood (full)**	**Mk1 Likelihood (part)**	**Mk2 Likelihood (full)**	**Mk2 Likelihood (part)**
**Alismatales**	whorled	whorled**	whorled**	whorled*	whorled**
**Apiales**	whorled	whorled**	whorled**	whorled**	whorled**
**Aquifoliales**	whorled	whorled**	whorled**	whorled**	whorled**
**Arecales**	whorled	whorled**	whorled**	whorled**	whorled**
**Asparagales**	whorled	whorled**	whorled**	whorled**	whorled**
**Asterales**	whorled	whorled**	whorled**	whorled**	whorled**
**Austrobaileyales**	spiral	**spiral****	**whorled**	**spiral****	**whorled**
**Berberidopsidales**	whorled	whorled**	whorled**	whorled*	whorled*
**Boraginales**	whorled	whorled**	whorled**	whorled**	whorled**
**Brassicales**	whorled	whorled**	whorled**	whorled**	whorled**
**Bruniales**	whorled	whorled**	whorled**	whorled**	whorled**
**Buxales**	whorled	**whorled****	whorled**	**spiral***	**whorled***
**Canellales**	whorled	**whorled****	whorled**	**spiral****	**whorled****
**Caryophyllales**	whorled	whorled**	whorled**	whorled**	whorled**

**Celastrales**	whorled	whorled**	whorled**	whorled**	whorled**
**Commelinales**	whorled	whorled**	whorled**	whorled**	whorled**
**Cornales**	whorled	whorled**	whorled**	whorled**	whorled**
**Crossosomatales**	whorled	whorled**	whorled**	whorled**	whorled**
**Cucurbitales**	whorled	whorled**	whorled**	whorled**	whorled**
**Dioscoreales**	whorled	whorled**	whorled**	whorled**	whorled**
**Dipsacales**	whorled	whorled**	whorled**	whorled**	whorled**
**Ericales**	whorled	whorled**	whorled**	whorled**	whorled**
**Escalloniales**	whorled	whorled**	whorled**	whorled**	whorled**
**Fabales**	whorled	whorled**	whorled**	whorled**	whorled**
**Fagales**	whorled	whorled**	whorled**	whorled**	whorled**
**Garryales**	whorled	whorled**	whorled**	whorled**	whorled**
**Gentianales**	whorled	whorled**	whorled**	whorled**	whorled**
**Geraniales**	whorled	whorled**	whorled**	whorled**	whorled**
**Huerteales**	whorled	whorled**	whorled**	whorled**	whorled**
**Icacinales**	whorled	whorled**	whorled**	whorled**	whorled**
**Lamiales**	whorled	whorled**	whorled**	whorled**	whorled**
**Laurales**	whorled	**whorled****	whorled**	**spiral****	**whorled****
**Liliales**	whorled	whorled**	whorled**	whorled**	whorled**
**Magnoliales**	whorled	**whorled****	whorled**	**spiral****	**whorled****
**Malpighiales**	whorled	whorled**	whorled**	whorled**	whorled**
**Malvales**	whorled	whorled**	whorled**	whorled**	whorled**
**Myrtales**	whorled	whorled**	whorled**	whorled**	whorled**
**Nymphaeales**	whorled, spiral	**whorled****	whorled**	**spiral***	**whorled****
**Oxalidales**	whorled	whorled**	whorled**	whorled**	whorled**
**Pandanales**	whorled	whorled**	whorled**	whorled**	whorled**
**Paracryphiales**	whorled	whorled**	whorled**	whorled**	whorled**
**Petrosaviales**	whorled	whorled**	whorled**	whorled**	whorled**
**Piperales**	whorled	whorled**	whorled**	whorled	whorled**
**Poales**	whorled	whorled**	whorled**	whorled**	whorled**
**Proteales**	whorled	**whorled****	whorled**	**spiral****	**whorled***
**Ranunculales**	whorled	**whorled****	whorled**	**spiral****	**whorled***
**Rosales**	whorled	whorled**	whorled**	whorled**	whorled**
**Santalales**	whorled	whorled**	whorled**	whorled**	whorled**
**Sapindales**	whorled	whorled**	whorled**	whorled**	whorled**

**Saxifragales**	whorled	whorled**	whorled**	whorled**	whorled**
**Solanales**	whorled	whorled**	whorled**	whorled**	whorled**
**Vitales**	whorled	whorled**	whorled**	whorled**	whorled**
**Zingiberales**	whorled	whorled**	whorled**	whorled**	whorled**
**Zygophyllales**	whorled	whorled**	whorled**	whorled**	whorled**

**ORIGINAL DATA (Five-char)**	**Parsimony**	**Maximum Likelihood (Mk1 model)**		**Maximum Likelihood (Mk2 model)**	
	**Both "full" and "part"**	**Mk1 Likelihood (full)**	**Mk1 Likelihood (part)**	**Mk2 Likelihood (full)**	**Mk2 Likelihood (part)**
**Alismatales**	whorled	whorled**	whorled**	whorled*	whorled**
**Apiales**	whorled	whorled**	whorled**	whorled**	whorled**
**Aquifoliales**	whorled	whorled**	whorled**	whorled**	whorled**
**Arecales**	whorled	whorled**	whorled**	whorled**	whorled**
**Asparagales**	whorled	whorled**	whorled**	whorled**	whorled**
**Asterales**	whorled	whorled**	whorled**	whorled**	whorled**
**Austrobaileyales**	spiral	**spiral****	**whorled**	**spiral****	**whorled**
**Berberidopsidales**	whorled	whorled**	whorled**	whorled*	whorled*
**Boraginales**	whorled	whorled**	whorled**	whorled**	whorled**
**Brassicales**	whorled	whorled**	whorled**	whorled**	whorled**
**Bruniales**	whorled	whorled**	whorled**	whorled**	whorled**
**Buxales**	whorled	whorled**	whorled**	whorled*	whorled*
**Canellales**	whorled	**whorled****	whorled**	**spiral****	**whorled****
**Caryophyllales**	whorled	whorled**	whorled**	whorled**	whorled**
**Celastrales**	whorled	whorled**	whorled**	whorled**	whorled**
**Commelinales**	whorled	whorled**	whorled**	whorled**	whorled**
**Cornales**	whorled	whorled**	whorled**	whorled**	whorled**
**Crossosomatales**	whorled	whorled**	whorled**	whorled**	whorled**
**Cucurbitales**	whorled	whorled**	whorled**	whorled**	whorled**
**Dioscoreales**	whorled	whorled**	whorled**	whorled**	whorled**
**Dipsacales**	whorled	whorled**	whorled**	whorled**	whorled**
**Ericales**	whorled	whorled**	whorled**	whorled**	whorled**
**Escalloniales**	whorled	whorled**	whorled**	whorled**	whorled**
**Fabales**	whorled	whorled**	whorled**	whorled**	whorled**
**Fagales**	whorled	whorled**	whorled**	whorled**	whorled**
**Garryales**	whorled	whorled**	whorled**	whorled**	whorled**

Gentianales	whorled	whorled**	whorled**	whorled**	whorled**
Geraniales	whorled	whorled**	whorled**	whorled**	whorled**
Huerteales	whorled	whorled**	whorled**	whorled**	whorled**
Icacinales	whorled	whorled**	whorled**	whorled**	whorled**
Lamiales	whorled	whorled**	whorled**	whorled**	whorled**
Laurales	whorled	**whorled****	whorled**	**spiral****	**whorled****
Liliales	whorled	whorled**	whorled**	whorled**	whorled**
Magnoliales	whorled	**whorled****	whorled**	**spiral****	**whorled****
Malpighiales	whorled	whorled**	whorled**	whorled**	whorled**
Malvales	whorled	whorled**	whorled**	whorled**	whorled**
Myrtales	whorled	whorled**	whorled**	whorled**	whorled**
Nymphaeales	whorled, spiral	**whorled****	whorled**	**spiral***	**whorled****
Oxalidales	whorled	whorled**	whorled**	whorled**	whorled**
Pandanales	whorled	whorled**	whorled**	whorled**	whorled**
Paracryphiales	whorled	whorled**	whorled**	whorled**	whorled**
Petrosaviales	whorled	whorled**	whorled**	whorled**	whorled**
Piperales	whorled	whorled**	whorled**	whorled	whorled**
Poales	whorled	whorled**	whorled**	whorled**	whorled**
Proteales	whorled	**whorled****	whorled**	**spiral****	**whorled***
Ranunculales	whorled	**whorled****	whorled**	**spiral****	**whorled***
Rosales	whorled	whorled**	whorled**	whorled**	whorled**
Santalales	whorled	whorled**	whorled**	whorled**	whorled**
Sapindales	whorled	whorled**	whorled**	whorled**	whorled**
Saxifragales	whorled	whorled**	whorled**	whorled**	whorled**
Solanales	whorled	whorled**	whorled**	whorled**	whorled**
Vitales	whorled	whorled**	whorled**	whorled**	whorled**
Zingiberales	whorled	whorled**	whorled**	whorled**	whorled**
Zygophyllales	whorled	whorled**	whorled**	whorled**	whorled**

RESCORED DATA (PrunedMB)	Parsimony	Maximum Likelihood (Mk1 model)		Maximum Likelihood (Mk2 model)	
	Both "full" and "part"	Mk1 Likelihood (full)	Mk1 Likelihood (part)	Mk2 Likelihood (full)	Mk2 Likelihood (part)
Alismatales	whorled	whorled**	whorled**	whorled*	whorled**
Apiales	whorled	whorled**	whorled**	whorled**	whorled**
Aquifoliales	whorled	whorled**	whorled**	whorled**	whorled**

**Arecales**	whorled	whorled**	whorled**	whorled**	whorled**
**Asparagales**	whorled	whorled**	whorled**	whorled**	whorled**
**Asterales**	whorled	whorled**	whorled**	whorled**	whorled**
**Austrobaileyales**	spiral	**spiral****	**whorled**	**spiral****	**whorled**
**Berberidopsidales**	whorled	whorled**	whorled**	whorled*	whorled*
**Boraginales**	whorled	whorled**	whorled**	whorled**	whorled**
**Brassicales**	whorled	whorled**	whorled**	whorled**	whorled**
**Bruniales**	whorled	whorled**	whorled**	whorled**	whorled**
**Buxales**	whorled	**whorled****	whorled*	**spiral***	**whorled***
**Canellales**	whorled	**whorled****	whorled**	**spiral****	**whorled**
**Caryophyllales**	whorled	whorled**	whorled**	whorled**	whorled**
**Celastrales**	whorled	whorled**	whorled**	whorled**	whorled**
**Commelinales**	whorled	whorled**	whorled**	whorled**	whorled**
**Cornales**	whorled	whorled**	whorled**	whorled**	whorled**
**Crossosomatales**	whorled	whorled**	whorled**	whorled**	whorled**
**Cucurbitales**	whorled	whorled**	whorled**	whorled**	whorled**
**Dioscoreales**	whorled	whorled**	whorled**	whorled**	whorled**
**Dipsacales**	whorled	whorled**	whorled**	whorled**	whorled**
**Ericales**	whorled	whorled**	whorled**	whorled**	whorled**
**Escalloniales**	whorled	whorled**	whorled**	whorled**	whorled**
**Fabales**	whorled	whorled**	whorled**	whorled**	whorled**
**Fagales**	whorled	whorled**	whorled**	whorled**	whorled**
**Garryales**	whorled	whorled**	whorled**	whorled**	whorled**
**Gentianales**	whorled	whorled**	whorled**	whorled**	whorled**
**Geraniales**	whorled	whorled**	whorled**	whorled**	whorled**
**Huerteales**	whorled	whorled**	whorled**	whorled**	whorled**
**Icacinales**	whorled	whorled**	whorled**	whorled**	whorled**
**Lamiales**	whorled	whorled**	whorled**	whorled**	whorled**
**Laurales**	whorled	**whorled****	whorled**	**spiral****	**whorled**
**Liliales**	whorled	whorled**	whorled**	whorled**	whorled**
**Magnoliales**	whorled	**whorled****	**whorled****	**spiral****	**spiral**
**Malpighiales**	whorled	whorled**	whorled**	whorled**	whorled**
**Malvales**	whorled	whorled**	whorled**	whorled**	whorled**
**Myrtales**	whorled	whorled**	whorled**	whorled**	whorled**
**Nymphaeales**	whorled, spiral	**whorled****	whorled**	**spiral***	**whorled***

**Oxalidales**	whorled	whorled**	whorled**	whorled**	whorled**
**Pandanales**	whorled	whorled**	whorled**	whorled**	whorled**
**Paracryphiales**	whorled	whorled**	whorled**	whorled**	whorled**
**Petrosaviales**	whorled	whorled**	whorled**	whorled**	whorled**
**Piperales**	whorled	whorled**	whorled**	whorled	whorled*
**Poales**	whorled	whorled**	whorled**	whorled**	whorled**
**Proteales**	whorled	**whorled****	whorled**	**spiral****	**whorled***
**Ranunculales**	whorled	**whorled****	whorled**	**spiral****	**whorled***
**Rosales**	whorled	whorled**	whorled**	whorled**	whorled**
**Santalales**	whorled	whorled**	whorled**	whorled**	whorled**
**Sapindales**	whorled	whorled**	whorled**	whorled**	whorled**
**Saxifragales**	whorled	whorled**	whorled**	whorled**	whorled**
**Solanales**	whorled	whorled**	whorled**	whorled**	whorled**
**Vitales**	whorled	whorled**	whorled**	whorled**	whorled**
**Zingiberales**	whorled	whorled**	whorled**	whorled**	whorled**
**Zygophyllales**	whorled	whorled**	whorled**	whorled**	whorled**

Merism

high rate= high rate equilibrium; full = full tree; part = partitioned tree; **state differences between comparable models**

**RESCORED DATA (Five-char)**	**Parsimony**	**Maximum Likelihood (Mk1 model)**		**Maximum Likelihood (Mk2 model)**	
	**Both "full" and "part"**	**Mk1 Likelihood (full)**	**Mk1 Likelihood (part)**	**Mk2 Likelihood (full)**	**Mk2 Likelihood (part)**
**Alismatales**	non-penta	non-penta**	non-penta**	non-penta**	non-penta**
**Apiales**	pentamerous	pentamerous**	pentamerous**	pentamerous**	pentamerous*
**Aquifoliales**	pentamerous	pentamerous**	pentamerous**	pentamerous**	pentamerous*
**Arecales**	non-penta	non-penta**	non-penta**	non-penta**	non-penta**
**Asparagales**	non-penta	non-penta**	non-penta**	non-penta**	non-penta**
**Asterales**	pentamerous	pentamerous**	pentamerous**	pentamerous**	pentamerous*
**Austrobaileyales**	?	?	?	?	?
**Berberidopsidales**	pentamerous	?	?	?	?
**Boraginales**	pentamerous	pentamerous**	pentamerous**	pentamerous**	pentamerous*
**Brassicales**	pentamerous	pentamerous**	pentamerous**	pentamerous**	pentamerous
**Bruniales**	pentamerous	pentamerous**	pentamerous**	pentamerous**	pentamerous*
**Buxales**	non-penta	non-penta**	non-penta*	non-penta*	non-penta*

**Canellales**	non-penta	non-penta**	non-penta**	non-penta**	non-penta*
**Caryophyllales**	pentamerous	pentamerous**	pentamerous**	pentamerous**	pentamerous*
**Celastrales**	pentamerous	pentamerous**	pentamerous*	pentamerous**	pentamerous*
**Commelinales**	non-penta	non-penta**	non-penta**	non-penta**	non-penta**
**Cornales**	pentamerous	pentamerous**	pentamerous**	pentamerous**	pentamerous*
**Crossosomatales**	pentamerous	pentamerous**	pentamerous**	pentamerous**	pentamerous*
**Cucurbitales**	pentamerous	pentamerous**	pentamerous*	pentamerous**	pentamerous*
**Dioscoreales**	non-penta	non-penta**	non-penta**	non-penta**	non-penta**
**Dipsacales**	pentamerous	pentamerous**	pentamerous**	pentamerous**	pentamerous*
**Ericales**	pentamerous	pentamerous**	pentamerous**	pentamerous**	pentamerous*
**Escalloniales**	pentamerous	pentamerous**	pentamerous**	pentamerous**	pentamerous*
**Fabales**	pentamerous	pentamerous**	pentamerous**	pentamerous**	pentamerous*
**Fagales**	non-penta	pentamerous	**pentamerous***	**pentamerous**	**equivocal**
**Garryales**	non-penta, pentamerous	non-penta*	non-penta*	non-penta*	non-penta*
**Gentianales**	pentamerous	pentamerous**	pentamerous**	pentamerous**	pentamerous*
**Geraniales**	pentamerous	pentamerous**	pentamerous**	pentamerous**	pentamerous*
**Huerteales**	pentamerous	pentamerous**	pentamerous**	pentamerous**	pentamerous*
**Icacinales**	pentamerous	pentamerous**	pentamerous*	pentamerous**	pentamerous*
**Lamiales**	pentamerous	pentamerous**	pentamerous**	pentamerous**	pentamerous*
**Laurales**	non-penta	non-penta**	non-penta**	non-penta**	non-penta*
**Liliales**	non-penta	non-penta**	non-penta**	non-penta**	non-penta**
**Magnoliales**	non-penta	non-penta**	non-penta**	non-penta**	non-penta*
**Malpighiales**	pentamerous	pentamerous**	pentamerous**	pentamerous**	pentamerous*
**Malvales**	pentamerous	pentamerous**	pentamerous**	pentamerous**	pentamerous*
**Myrtales**	pentamerous	pentamerous**	pentamerous*	pentamerous**	pentamerous
**Nymphaeales**	non-penta	non-penta**	non-penta**	non-penta**	non-penta*
**Oxalidales**	pentamerous	pentamerous**	pentamerous**	pentamerous**	pentamerous*
**Pandanales**	non-penta	non-penta**	non-penta**	non-penta**	non-penta**
**Paracryphiales**	pentamerous	pentamerous*	pentamerous*	pentamerous*	pentamerous*
**Petrosaviales**	non-penta	non-penta**	non-penta**	non-penta**	non-penta**
**Piperales**	non-penta	non-penta**	non-penta**	non-penta**	non-penta**
**Poales**	non-penta	non-penta**	non-penta**	non-penta**	non-penta**
**Proteales**	non-penta, pentamerous	non-penta*	non-penta**	non-penta*	non-penta*
**Ranunculales**	non-penta	non-penta**	non-penta**	non-penta**	non-penta*

Rosales	pentamerous	pentamerous**	pentamerous*	pentamerous**	pentamerous
Santalales	pentamerous	pentamerous*	pentamerous*	pentamerous**	pentamerous*
Sapindales	pentamerous	pentamerous**	pentamerous**	pentamerous**	pentamerous
Saxifragales	pentamerous	pentamerous**	pentamerous**	pentamerous**	pentamerous
Solanales	pentamerous	pentamerous**	pentamerous**	pentamerous**	pentamerous*
Vitales	pentamerous	pentamerous*	pentamerous*	pentamerous*	pentamerous*
Zingiberales	non-penta	non-penta**	non-penta**	non-penta**	non-penta**
Zygophyllales	pentamerous	pentamerous**	pentamerous*	pentamerous**	pentamerous*

ORIGINAL DATA (Five-char)	Parsimony	Maximum Likelihood (Mk1 model)		Maximum Likelihood (Mk2 model)	
	Both "full" and "part"	Mk1 Likelihood (full)	Mk1 Likelihood (part)	Mk2 Likelihood (full)	Mk2 Likelihood (part)
Alismatales	non-penta	non-penta**	non-penta**	non-penta**	non-penta**
Apiales	pentamerous	pentamerous**	pentamerous**	pentamerous**	pentamerous*
Aquifoliales	pentamerous	pentamerous**	pentamerous**	pentamerous**	pentamerous*
Arecales	non-penta	non-penta**	non-penta**	non-penta**	non-penta**
Asparagales	non-penta	non-penta**	non-penta**	non-penta**	non-penta**
Asterales	pentamerous	pentamerous**	pentamerous**	pentamerous**	pentamerous*
Austrobaileyales	?	?	?	?	?
Berberidopsidales	non-penta, pentamerous	?	?	?	?
Boraginales	pentamerous	pentamerous**	pentamerous**	pentamerous**	pentamerous*
Brassicales	pentamerous	pentamerous**	**pentamerous***	**pentamerous****	**non-penta**
Bruniales	pentamerous	pentamerous**	pentamerous**	pentamerous**	pentamerous*
Buxales	non-penta	non-penta*	non-penta**	non-penta*	non-penta**
Canellales	non-penta	non-penta**	non-penta**	non-penta**	non-penta**
Caryophyllales	pentamerous	pentamerous**	pentamerous**	pentamerous**	pentamerous*
Celastrales	pentamerous	pentamerous**	pentamerous*	pentamerous**	pentamerous
Commelinales	non-penta	non-penta**	non-penta**	non-penta**	non-penta**
Cornales	pentamerous	pentamerous**	pentamerous*	pentamerous**	pentamerous
Crossosomatales	pentamerous	pentamerous**	pentamerous*	pentamerous**	pentamerous
Cucurbitales	non-penta	pentamerous	**pentamerous**	**pentamerous****	**non-penta***
Dioscoreales	non-penta	non-penta**	non-penta**	non-penta**	non-penta**
Dipsacales	pentamerous	pentamerous**	pentamerous**	pentamerous**	pentamerous*
Ericales	pentamerous	pentamerous**	pentamerous**	pentamerous**	pentamerous*

**Escalloniales**	pentamerous	pentamerous**	pentamerous**	pentamerous**	pentamerous*
**Fabales**	pentamerous	pentamerous**	pentamerous**	pentamerous**	pentamerous*
**Fagales**	non-penta	**non-penta***	**pentamerous**	**pentamerous**	**non-penta***
**Garryales**	non-penta, pentamerous	non-penta*	non-penta*	non-penta*	non-penta*
**Gentianales**	pentamerous	pentamerous**	pentamerous**	pentamerous**	pentamerous
**Geraniales**	pentamerous	pentamerous**	**pentamerous****	**pentamerous****	**non-penta**
**Huerteales**	pentamerous	pentamerous**	pentamerous*	pentamerous**	pentamerous*
**Icacinales**	pentamerous	pentamerous**	pentamerous*	pentamerous**	pentamerous*
**Lamiales**	pentamerous	pentamerous**	pentamerous**	pentamerous**	pentamerous*
**Laurales**	non-penta	non-penta**	non-penta**	non-penta**	non-penta**
**Liliales**	non-penta	non-penta**	non-penta**	non-penta**	non-penta**
**Magnoliales**	non-penta	non-penta**	non-penta**	non-penta**	non-penta**
**Malpighiales**	pentamerous	pentamerous**	pentamerous**	pentamerous**	pentamerous*
**Malvales**	pentamerous	pentamerous**	pentamerous*	pentamerous**	pentamerous
**Myrtales**	pentamerous	pentamerous**	**pentamerous***	**pentamerous****	**non-penta**
**Nymphaeales**	non-penta	non-penta**	non-penta**	non-penta**	non-penta**
**Oxalidales**	pentamerous	pentamerous**	**pentamerous****	**pentamerous****	**equivocal**
**Pandanales**	non-penta	non-penta**	non-penta**	non-penta**	non-penta**
**Paracryphiales**	pentamerous	pentamerous*	pentamerous*	pentamerous**	pentamerous*
**Petrosaviales**	non-penta	non-penta**	non-penta**	non-penta*	non-penta**
**Piperales**	non-penta	non-penta**	non-penta**	non-penta**	non-penta**
**Poales**	non-penta	non-penta**	non-penta**	non-penta**	non-penta**
**Proteales**	non-penta	non-penta**	non-penta**	non-penta*	non-penta*
**Ranunculales**	non-penta	non-penta**	non-penta**	non-penta**	non-penta**
**Rosales**	non-penta	pentamerous*	**pentamerous***	**pentamerous****	**non-penta***
**Santalales**	non-penta, pentamerous	**non-penta***	**pentamerous**	**pentamerous****	pentamerous
**Sapindales**	pentamerous	pentamerous**	**pentamerous***	**pentamerous****	**non-penta***
**Saxifragales**	pentamerous	pentamerous*	**pentamerous***	**pentamerous****	**non-penta**
**Solanales**	pentamerous	pentamerous**	pentamerous**	pentamerous**	pentamerous*
**Vitales**	pentamerous	pentamerous*	pentamerous*	pentamerous*	pentamerous
**Zingiberales**	non-penta	non-penta**	non-penta**	non-penta**	non-penta**
**Zygophyllales**	pentamerous	pentamerous**	pentamerous*	pentamerous**	pentamerous*

RESCORED DATA (PrunedMB)	Parsimony	Maximum Likelihood (Mk1 model)		Maximum Likelihood (Mk2 model)	
	Both "full" and "part"	Mk1 Likelihood (full)	Mk1 Likelihood (part)	Mk2 Likelihood (full)	Mk2 Likelihood (part)
**Alismatales**	non-penta	non-penta**	non-penta**	non-penta**	non-penta**
**Apiales**	pentamerous	pentamerous**	equivocal (high rate)	pentamerous**	pentamerous (high rate)
**Aquifoliales**	pentamerous	pentamerous**	equivocal (high rate)	pentamerous**	pentamerous (high rate)
**Arecales**	non-penta	non-penta**	non-penta**	non-penta**	non-penta**
**Asparagales**	non-penta	non-penta**	non-penta**	non-penta**	non-penta**
**Asterales**	pentamerous	pentamerous**	equivocal (high rate)	pentamerous**	pentamerous (high rate)
**Austrobaileyales**	?	?	?	?	?
**Berberidopsidales**	pentamerous	?	?	?	?
**Boraginales**	pentamerous	pentamerous**	equivocal (high rate)	pentamerous**	pentamerous (high rate)
**Brassicales**	pentamerous	pentamerous**	**pentamerous***	**pentamerous****	**equivocal**
**Bruniales**	pentamerous	pentamerous**	equivocal (high rate)	pentamerous**	pentamerous (high rate)
**Buxales**	non-penta	non-penta**	non-penta**	non-penta*	non-penta**
**Canellales**	non-penta	non-penta**	non-penta**	non-penta**	non-penta*
**Caryophyllales**	pentamerous	pentamerous**	equivocal (high rate)	pentamerous**	pentamerous (high rate)
**Celastrales**	pentamerous	pentamerous**	pentamerous*	pentamerous**	pentamerous*
**Commelinales**	non-penta	non-penta**	non-penta**	non-penta**	non-penta**
**Cornales**	pentamerous	pentamerous**	equivocal (high rate)	pentamerous**	pentamerous (high rate)
**Crossosomatales**	pentamerous	pentamerous**	pentamerous*	pentamerous**	pentamerous*
**Cucurbitales**	pentamerous	pentamerous**	pentamerous*	pentamerous**	pentamerous*
**Dioscoreales**	non-penta	non-penta**	non-penta**	non-penta**	non-penta**
**Dipsacales**	pentamerous	pentamerous**	equivocal (high rate)	pentamerous**	pentamerous (high rate)
**Ericales**	pentamerous	pentamerous**	equivocal (high rate)	pentamerous**	pentamerous (high rate)
**Escalloniales**	pentamerous	pentamerous**	equivocal (high rate)	pentamerous**	pentamerous (high rate)
**Fabales**	pentamerous	pentamerous**	pentamerous**	pentamerous**	pentamerous*
**Fagales**	non-penta	pentamerous	pentamerous*	pentamerous	pentamerous
**Garryales**	non-penta, pentamerous	non-penta**	equivocal (high rate)	non-penta**	pentamerous (high rate)

**Gentianales**	pentamerous	pentamerous**	equivocal (high rate)	pentamerous**	pentamerous (high rate)
**Geraniales**	pentamerous	pentamerous**	pentamerous**	pentamerous**	pentamerous*
**Huerteales**	pentamerous	pentamerous**	pentamerous*	pentamerous**	pentamerous*
**Icacinales**	non-penta, pentamerous	non-penta	equivocal (high rate)	pentamerous	pentamerous (high rate)
**Lamiales**	pentamerous	pentamerous**	equivocal (high rate)	pentamerous**	pentamerous (high rate)
**Laurales**	non-penta	non-penta**	non-penta**	non-penta**	non-penta*
**Liliales**	non-penta	non-penta**	non-penta**	non-penta**	non-penta**
**Magnoliales**	non-penta	non-penta**	non-penta**	non-penta**	non-penta*
**Malpighiales**	pentamerous	pentamerous**	pentamerous**	pentamerous**	pentamerous*
**Malvales**	pentamerous	pentamerous**	pentamerous*	pentamerous**	pentamerous*
**Myrtales**	pentamerous	pentamerous**	pentamerous*	pentamerous**	pentamerous
**Nymphaeales**	non-penta	non-penta**	non-penta**	non-penta**	non-penta*
**Oxalidales**	pentamerous	pentamerous**	pentamerous**	pentamerous**	pentamerous*
**Pandanales**	non-penta	non-penta**	non-penta**	non-penta**	non-penta**
**Paracryphiales**	pentamerous	pentamerous*	equivocal (high rate)	pentamerous*	pentamerous (high rate)
**Petrosaviales**	non-penta	non-penta**	non-penta**	non-penta**	non-penta**
**Piperales**	non-penta	non-penta**	non-penta**	non-penta**	non-penta**
**Poales**	non-penta	non-penta**	non-penta**	non-penta**	non-penta**
**Proteales**	non-penta, pentamerous	non-penta*	non-penta*	non-penta*	non-penta
**Ranunculales**	non-penta	non-penta**	non-penta**	non-penta**	non-penta*
**Rosales**	non-penta, pentamerous	pentamerous**	pentamerous*	pentamerous**	pentamerous*
**Santalales**	pentamerous	pentamerous*	equivocal (high rate)	pentamerous**	pentamerous (high rate)
**Sapindales**	pentamerous	pentamerous**	pentamerous**	pentamerous**	pentamerous*
**Saxifragales**	pentamerous	pentamerous**	pentamerous**	pentamerous**	pentamerous*
**Solanales**	pentamerous	pentamerous**	equivocal (high rate)	pentamerous**	pentamerous (high rate)
**Vitales**	pentamerous	pentamerous*	pentamerous*	pentamerous*	pentamerous*
**Zingiberales**	non-penta	non-penta**	non-penta**	non-penta**	non-penta**
**Zygophyllales**	pentamerous	pentamerous**	pentamerous*	pentamerous**	pentamerous*

Differentiation

high rate= high rate equilibrium; full = full tree; part = partitioned tree; **state differences between comparable models**

RESCORED DATA (Five-char)	Parsimony	Maximum Likelihood (Mk1 model)		Maximum Likelihood (Mk2 model)	
	Both "full" and "part"	Mk1 Likelihood (full)	Mk1 Likelihood (part)	Mk2 Likelihood (full)	Mk2 Likelihood (part)
**Alismatales**	undifferentiated	**differentiated***	**undifferentiated**	**undifferentiated ** **	undifferentiated*
**Apiales**	differentiated	differentiated**	differentiated**	differentiated**	differentiated**
**Aquifoliales**	differentiated	differentiated**	differentiated**	differentiated**	differentiated**
**Arecales**	undifferentiated, differentiated	**differentiated***	differentiated	**undifferentiated ***	**differentiated**
**Asparagales**	undifferentiated, differentiated	**differentiated***	**undifferentiated**	**undifferentiated ** **	undifferentiated
**Asterales**	differentiated	differentiated**	differentiated**	differentiated**	differentiated**
**Austrobaileyales**	undifferentiated	**differentiated***	**equivocal**	**undifferentiated ** **	**undifferentiated**
**Berberidopsidales**	differentiated	differentiated*	differentiated*	differentiated*	differentiated*
**Boraginales**	differentiated	differentiated**	differentiated**	differentiated**	differentiated**
**Brassicales**	differentiated	differentiated**	differentiated**	differentiated**	differentiated**
**Bruniales**	differentiated	differentiated**	differentiated**	differentiated**	differentiated**
**Buxales**	differentiated	differentiated**	differentiated*	differentiated	differentiated**
**Canellales**	differentiated	**differentiated****	differentiated	**undifferentiated ** **	**differentiated**
**Caryophyllales**	differentiated	differentiated**	differentiated**	differentiated*	differentiated*
**Celastrales**	differentiated	differentiated**	differentiated**	differentiated**	differentiated**
**Commelinales**	differentiated	differentiated**	differentiated**	differentiated*	differentiated**
**Cornales**	differentiated	differentiated**	differentiated**	differentiated**	differentiated**
**Crossosomatales**	differentiated	differentiated**	differentiated**	differentiated**	differentiated**
**Cucurbitales**	differentiated	**differentiated****	differentiated**	**undifferentiated**	**differentiated**
**Dioscoreales**	undifferentiated	undifferentiated	undifferentiated	undifferentiated* *	undifferentiated
**Dipsacales**	differentiated	differentiated**	differentiated**	differentiated**	differentiated**
**Ericales**	differentiated	differentiated**	differentiated**	differentiated**	differentiated**
**Escalloniales**	differentiated	differentiated**	differentiated**	differentiated**	differentiated**
**Fabales**	differentiated	differentiated**	differentiated**	differentiated**	differentiated**
**Fagales**	undifferentiated	undifferentiated*	undifferentiated*	undifferentiated* *	undifferentiated*
**Garryales**	differentiated	differentiated**	differentiated**	differentiated*	differentiated*
**Gentianales**	differentiated	differentiated**	differentiated**	differentiated**	differentiated**
**Geraniales**	differentiated	differentiated**	differentiated**	differentiated**	differentiated**

Huerteales	differentiated	__differentiated*__	differentiated*	__equivocal__	__differentiated__
Icacinales	differentiated	differentiated**	differentiated**	differentiated**	differentiated**
Lamiales	differentiated	differentiated**	differentiated**	differentiated**	differentiated**
Laurales	undifferentiated	__differentiated*__	__differentiated__	__undifferentiated**__	__undifferentiated__
Liliales	undifferentiated, differentiated	__differentiated*__	differentiated	__undifferentiated**__	__differentiated__
Magnoliales	undifferentiated	__differentiated**__	differentiated*	__undifferentiated*__	__differentiated__
Malpighiales	differentiated	differentiated**	differentiated**	differentiated**	differentiated**
Malvales	differentiated	differentiated**	differentiated**	differentiated**	differentiated**
Myrtales	differentiated	differentiated**	differentiated**	differentiated**	differentiated**
Nymphaeales	undifferentiated	__differentiated*__	__differentiated__	__undifferentiated**__	__equivocal__
Oxalidales	differentiated	differentiated**	differentiated**	differentiated**	differentiated**
Pandanales	undifferentiated	undifferentiated**	undifferentiated*	undifferentiated**	undifferentiated*
Paracryphiales	differentiated	differentiated*	differentiated**	differentiated*	differentiated*
Petrosaviales	differentiated	__differentiated*__	differentiated	__undifferentiated*__	__differentiated__
Piperales	undifferentiated	__differentiated__	undifferentiated	__undifferentiated**__	undifferentiated
Poales	differentiated	__differentiated**__	differentiated*	__undifferentiated**__	__differentiated*__
Proteales	differentiated	__differentiated**__	differentiated**	__undifferentiated*__	__differentiated**__
Ranunculales	differentiated	__differentiated**__	differentiated**	__undifferentiated*__	__differentiated**__
Rosales	differentiated	__differentiated*__	__differentiated*__	__undifferentiated*__	__undifferentiated*__
Santalales	differentiated	differentiated**	differentiated**	differentiated**	differentiated**
Sapindales	differentiated	differentiated**	differentiated**	differentiated**	differentiated**
Saxifragales	differentiated	differentiated**	differentiated**	differentiated**	differentiated**
Solanales	differentiated	differentiated**	differentiated**	differentiated**	differentiated**
Vitales	differentiated	differentiated**	differentiated**	differentiated**	differentiated**
Zingiberales	differentiated	differentiated**	differentiated*	differentiated*	differentiated*
Zygophyllales	differentiated	differentiated**	differentiated**	differentiated**	differentiated**

ORIGINAL DATA (Five-char)	Parsimony	Maximum Likelihood (Mk1 model)		Maximum Likelihood (Mk2 model)	

	Both "full" and "part"	Mk1 Likelihood (full)	Mk1 Likelihood (part)	Mk2 Likelihood (full)	Mk2 Likelihood (part)
**Alismatales**	undifferentiated	**differentiated***	**undifferentiated**	**undifferentiated ****	undifferentiated
**Apiales**	differentiated	differentiated**	differentiated**	differentiated**	differentiated**
**Aquifoliales**	differentiated	differentiated**	differentiated**	differentiated**	differentiated**
**Arecales**	undifferentiated, differentiated	**differentiated***	differentiated	**undifferentiated ***	**differentiated**
**Asparagales**	undifferentiated, differentiated	**differentiated**	**undifferentiated**	**undifferentiated ****	undifferentiated
**Asterales**	differentiated	differentiated**	differentiated**	differentiated**	differentiated**
**Austrobaileyales**	undifferentiated	**differentiated***	**undifferentiated**	**undifferentiated ****	undifferentiated
**Berberidopsidales**	differentiated	differentiated*	differentiated*	differentiated*	differentiated*
**Boraginales**	differentiated	differentiated**	differentiated**	differentiated**	differentiated**
**Brassicales**	differentiated	differentiated**	differentiated**	differentiated**	differentiated**
**Bruniales**	differentiated	differentiated**	differentiated**	differentiated**	differentiated**
**Buxales**	differentiated	differentiated**	differentiated**	differentiated	differentiated**
**Canellales**	differentiated	**differentiated***	**undifferentiated**	**undifferentiated ****	undifferentiated
**Caryophyllales**	differentiated	differentiated**	differentiated**	differentiated*	differentiated*
**Celastrales**	differentiated	differentiated**	differentiated**	differentiated**	differentiated**
**Commelinales**	differentiated	differentiated**	differentiated**	differentiated*	differentiated**
**Cornales**	differentiated	differentiated**	differentiated**	differentiated**	differentiated**
**Crossosomatales**	differentiated	differentiated**	differentiated**	differentiated**	differentiated**
**Cucurbitales**	differentiated	**differentiated****	differentiated**	**undifferentiated**	**differentiated**
**Dioscoreales**	undifferentiated	undifferentiated	undifferentiated	undifferentiated* *	undifferentiated
**Dipsacales**	differentiated	differentiated**	differentiated**	differentiated**	differentiated**
**Ericales**	differentiated	differentiated**	differentiated**	differentiated**	differentiated**
**Escalloniales**	differentiated	differentiated**	differentiated**	differentiated**	differentiated**
**Fabales**	differentiated	differentiated**	differentiated**	differentiated**	differentiated**
**Fagales**	undifferentiated	undifferentiated*	undifferentiated*	undifferentiated* *	undifferentiated*
**Garryales**	differentiated	differentiated**	differentiated**	differentiated*	differentiated*
**Gentianales**	differentiated	differentiated**	differentiated**	differentiated**	differentiated**
**Geraniales**	differentiated	differentiated**	differentiated**	differentiated**	differentiated**
**Huerteales**	differentiated	differentiated*	differentiated*	differentiated	differentiated
**Icacinales**	differentiated	differentiated**	differentiated**	differentiated**	differentiated**

Lamiales	differentiated	differentiated**	differentiated**	differentiated**	differentiated**
Laurales	undifferentiated	**differentiated***	**undifferentiated**	**undifferentiated****	undifferentiated
Liliales	undifferentiated, differentiated	**differentiated***	differentiated	**undifferentiated****	**differentiated**
Magnoliales	undifferentiated	**differentiated***	differentiated	**undifferentiated***	**differentiated**
Malpighiales	differentiated	differentiated**	differentiated**	differentiated**	differentiated**
Malvales	differentiated	differentiated**	differentiated**	differentiated**	differentiated**
Myrtales	differentiated	differentiated**	differentiated**	differentiated**	differentiated**
Nymphaeales	undifferentiated	**differentiated***	**undifferentiated**	**undifferentiated****	undifferentiated
Oxalidales	differentiated	differentiated**	differentiated**	differentiated**	differentiated**
Pandanales	undifferentiated	undifferentiated**	undifferentiated*	undifferentiated**	undifferentiated*
Paracryphiales	differentiated	differentiated*	differentiated**	differentiated*	differentiated*
Petrosaviales	differentiated	**differentiated***	differentiated	**undifferentiated***	**differentiated**
Piperales	undifferentiated	undifferentiated	undifferentiated*	undifferentiated**	undifferentiated*
Poales	differentiated	**differentiated***	differentiated*	**undifferentiated****	**differentiated**
Proteales	differentiated	**differentiated******	differentiated**	**undifferentiated***	**differentiated******
Ranunculales	differentiated	**differentiated******	differentiated**	**undifferentiated***	**differentiated******
Rosales	differentiated	**differentiated***	**differentiated***	**undifferentiated***	**undifferentiated***
Santalales	differentiated	differentiated**	differentiated**	differentiated**	differentiated**
Sapindales	differentiated	differentiated**	differentiated**	differentiated**	differentiated**
Saxifragales	differentiated	differentiated**	differentiated**	differentiated**	differentiated**
Solanales	differentiated	differentiated**	differentiated**	differentiated**	differentiated**
Vitales	differentiated	differentiated**	differentiated**	differentiated**	differentiated**
Zingiberales	differentiated	differentiated**	differentiated*	differentiated*	differentiated*
Zygophyllales	differentiated	differentiated**	differentiated**	differentiated**	differentiated**

RESCORED DATA (PrunedMB)	Parsimony	Maximum Likelihood (Mk1 model)		Maximum Likelihood (Mk2 model)	
	Both "full" and "part"	Mk1 Likelihood (full)	Mk1 Likelihood (part)	Mk2 Likelihood (full)	Mk2 Likelihood (part)
Alismatales	undifferentiated	**differentiated***	**undifferentiated**	**undifferentiated****	undifferentiated

**Apiales**	differentiated	differentiated**	differentiated**	differentiated**	differentiated**
**Aquifoliales**	differentiated	differentiated**	differentiated**	differentiated**	differentiated**
**Arecales**	undifferentiated. differentiated	**differentiated****	differentiated*	**undifferentiated***	**differentiated***
**Asparagales**	undifferentiated, differentiated	**differentiated**	**differentiated**	**undifferentiated****	undifferentiated
**Asterales**	differentiated	differentiated**	differentiated**	differentiated**	differentiated**
**Austrobaileyales**	undifferentiated	**differentiated***	equivocal (high rate)	**undifferentiated****	differentiated (high rate)
**Berberidopsidales**	differentiated	differentiated*	differentiated*	differentiated*	differentiated
**Boraginales**	differentiated	differentiated**	differentiated**	differentiated**	differentiated**
**Brassicales**	differentiated	differentiated**	differentiated**	differentiated**	differentiated**
**Bruniales**	differentiated	differentiated**	differentiated**	differentiated**	differentiated**
**Buxales**	differentiated	differentiated**	differentiated**	differentiated*	differentiated**
**Canellales**	differentiated	**differentiated****	equivocal (high rate)	**undifferentiated****	differentiated (high rate)
**Caryophyllales**	differentiated	differentiated**	**differentiated****	**differentiated***	**undifferentiated***
**Celastrales**	differentiated	differentiated**	differentiated**	differentiated**	differentiated**
**Commelinales**	differentiated	differentiated**	differentiated**	differentiated*	differentiated**
**Cornales**	differentiated	differentiated**	differentiated**	differentiated**	differentiated**
**Crossosomatales**	differentiated	differentiated**	differentiated**	differentiated**	differentiated**
**Cucurbitales**	differentiated	differentiated**	differentiated**	differentiated	differentiated*
**Dioscoreales**	undifferentiated	undifferentiated*	undifferentiated	undifferentiated**	undifferentiated
**Dipsacales**	differentiated	differentiated**	differentiated**	differentiated**	differentiated**
**Ericales**	differentiated	differentiated**	differentiated**	differentiated**	differentiated**
**Escalloniales**	differentiated	differentiated**	differentiated**	differentiated**	differentiated**
**Fabales**	differentiated	differentiated**	differentiated**	differentiated**	differentiated**
**Fagales**	undifferentiated	undifferentiated*	undifferentiated*	undifferentiated*	undifferentiated*
**Garryales**	differentiated	differentiated*	differentiated**	differentiated*	differentiated*
**Gentianales**	differentiated	differentiated**	differentiated**	differentiated**	differentiated**
**Geraniales**	differentiated	differentiated**	differentiated**	differentiated**	differentiated**
**Huerteales**	differentiated	**differentiated***	**differentiated***	**undifferentiated**	**undifferentiated**
**Icacinales**	differentiated	differentiated**	differentiated**	differentiated*	differentiated*
**Lamiales**	differentiated	differentiated**	differentiated**	differentiated**	differentiated**
**Laurales**	undifferentiated	**differentiated****	equivocal (high rate)	**undifferentiated****	differentiated (high rate)

**Liliales**	undifferentiated, differentiated	**differentiated***	undifferentiated	**undifferentiated ** **	undifferentiated
**Magnoliales**	undifferentiated	**differentiated****	equivocal (high rate)	**undifferentiated ***	differentiated (high rate)
**Malpighiales**	differentiated	differentiated**	differentiated**	differentiated**	differentiated**
**Malvales**	differentiated	differentiated**	differentiated**	differentiated**	differentiated**
**Myrtales**	differentiated	differentiated**	differentiated**	differentiated**	differentiated**
**Nymphaeales**	undifferentiated	**differentiated***	equivocal (high rate)	**undifferentiated ** **	differentiated (high rate)
**Oxalidales**	differentiated	differentiated**	differentiated**	differentiated**	differentiated**
**Pandanales**	undifferentiated	undifferentiated* *	undifferentiated*	undifferentiated* *	undifferentiated*
**Paracryphiales**	differentiated	differentiated*	differentiated**	differentiated*	differentiated*
**Petrosaviales**	differentiated	**differentiated***	differentiated	**undifferentiated ***	**differentiated**
**Piperales**	undifferentiated	**differentiated***	equivocal (high rate)	**undifferentiated ** **	differentiated (high rate)
**Poales**	differentiated	**differentiated****	differentiated*	**undifferentiated ** **	**differentiated***
**Proteales**	differentiated	**differentiated****	differentiated**	**undifferentiated ***	**differentiated****
**Ranunculales**	differentiated	**differentiated****	differentiated**	**undifferentiated ***	**differentiated****
**Rosales**	differentiated	**differentiated****	**differentiated****	**undifferentiated ***	**undifferentiated ***
**Santalales**	differentiated	differentiated**	differentiated**	differentiated**	differentiated*
**Sapindales**	differentiated	differentiated**	differentiated**	differentiated**	differentiated**
**Saxifragales**	differentiated	differentiated**	differentiated**	differentiated**	differentiated**
**Solanales**	differentiated	differentiated**	differentiated**	differentiated**	differentiated**
**Vitales**	differentiated	differentiated**	differentiated**	differentiated**	differentiated**
**Zingiberales**	differentiated	differentiated**	differentiated**	differentiated	differentiated**
**Zygophyllales**	differentiated	differentiated**	differentiated**	differentiated**	differentiated**

Testing the impact of morphological rate heterogeneity on ancestral state reconstruction of five floral traits in angiosperms

Supplementary Tables 4: Parsimony and Mk1 ancestral states with imposed rates for 15 key nodes

Elisabeth Reyes, Sophie Nadot, Maria von Balthazar, Jürg Schönenberger, Hervé Sauquet

Supplementary Tables 4: Parsimony and Mk1 ancestral states with imposed rates for 15 key nodes
Five-char: Results for main tree
PrunedMB: Results for the pruned Smith & Brown ALLMB tree

Symmetry
q= transition rate (changes per Ma); Parsi= parsimony; State proba= state probability; equi= equivocal; (hr)= high transition rate equilibrium; actino= actinomorphic

RESCORED DATA (Five-char)	Parsi	State for q=0.0001	State proba	State for q=0.001	State proba	State for q=0.01	State proba	State for q=0.1	State proba
**Angiospermae**	actino	actino	1	actino	1	actino	0.98	equi (hr)	0.5
**Mesangiospermae**	actino	actino	1	actino	1	actino	1	equi (hr)	0.5
**Magnoliidae**	actino	actino	1	actino	1	actino	1	equi (hr)	0.5
**Monocotyledoneae**	actino	actino	1	actino	1	actino	1	equi (hr)	0.5
**Commelinidae**	actino	actino	1	actino	1	actino	0.98	equi (hr)	0.5
**Eudicotyledoneae**	actino	actino	1	actino	1	actino	1	equi (hr)	0.5
**Pentapetalae**	actino	actino	1	actino	1	actino	1	equi (hr)	0.5
**Superrosidae**	actino	actino	1	actino	1	actino	1	equi (hr)	0.5
**Rosidae**	actino	actino	1	actino	1	actino	1	equi (hr)	0.5
**Malvidae**	actino	actino	1	actino	1	actino	1	equi (hr)	0.5
**Fabidae**	actino	actino	1	actino	1	actino	1	equi (hr)	0.5
**Superasteridae**	actino	actino	1	actino	1	actino	1	equi (hr)	0.5
**Asteridae**	actino	actino	1	actino	1	actino	1	equi (hr)	0.5
**Campanulidae**	actino	actino	1	actino	1	actino	1	equi (hr)	0.5
**Lamiidae**	actino	actino	1	actino	1	actino	0.99	equi (hr)	0.5

ORIGINAL DATA (Five-char)	Parsi	State for q=0.0001	State proba	State for q=0.001	State proba	State for q=0.01	State proba	State for q=0.1	State proba
**Angiospermae**	actino	actino	1	actino	1	actino	0.98	equi (hr)	0.5
**Mesangiospermae**	actino	actino	1	actino	1	actino	1	equi (hr)	0.5
**Magnoliidae**	actino	actino	1	actino	1	actino	1	equi (hr)	0.5
**Monocotyledoneae**	actino	actino	1	actino	1	actino	1	equi (hr)	0.5
**Commelinidae**	actino	actino	1	actino	1	actino	0.98	equi (hr)	0.5
**Eudicotyledoneae**	actino	actino	1	actino	1	actino	1	equi (hr)	0.5
**Pentapetalae**	actino	actino	1	actino	1	actino	1	equi (hr)	0.5
**Superrosidae**	actino	actino	1	actino	1	actino	1	equi (hr)	0.5
**Rosidae**	actino	actino	1	actino	1	actino	1	equi (hr)	0.5
**Malvidae**	actino	actino	1	actino	1	actino	1	equi (hr)	0.5
**Fabidae**	actino	actino	1	actino	1	actino	1	equi (hr)	0.5

**Superasteridae**	actino	actino	1	actino	1	actino	1	equi (hr)	0.5
**Asteridae**	actino	actino	1	actino	1	actino	1	equi (hr)	0.5
**Campanulidae**	actino	actino	1	actino	1	actino	1	equi (hr)	0.5
**Lamiidae**	actino	actino	1	actino	1	actino	0.99	equi (hr)	0.5

**RESCORED DATA (PrunedMB)**	**Parsi**	**State for q=0.0001**	**State proba**	**State for q=0.001**	**State proba**	**State for q=0.01**	**State proba**	**State for q=0.1**	**State proba**
**Angiospermae**	actino	actino	1	actino	1	actino	0.98	equi (hr)	0.5
**Mesangiospermae**	actino	actino	1	actino	1	actino	1	equi (hr)	0.5
**Magnoliidae**	actino	actino	1	actino	1	actino	1	equi (hr)	0.5
**Monocotyledoneae**	actino	actino	1	actino	1	actino	1	equi (hr)	0.5
**Commelinidae**	actino	actino	1	actino	1	actino	0.98	equi (hr)	0.5
**Eudicotyledoneae**	actino	actino	1	actino	1	actino	1	equi (hr)	0.5
**Pentapetalae**	actino	actino	1	actino	1	actino	1	equi (hr)	0.5
**Superrosidae**	actino	actino	1	actino	1	actino	1	equi (hr)	0.5
**Rosidae**	actino	actino	1	actino	1	actino	1	equi (hr)	0.5
**Malvidae**	actino	actino	1	actino	1	actino	1	equi (hr)	0.5
**Fabidae**	actino	actino	1	actino	1	actino	1	equi (hr)	0.5
**Superasteridae**	actino	actino	1	actino	1	actino	1	equi (hr)	0.5
**Asteridae**	actino	actino	1	actino	1	actino	1	equi (hr)	0.5
**Campanulidae**	actino	actino	1	actino	1	actino	1	equi (hr)	0.5
**Lamiidae**	actino	actino	1	actino	1	actino	0.99	equi (hr)	0.5

<u>Fusion</u>

q= transition rate (changes per Ma); Parsi= parsimony; State proba= state probability; equi= equivocal; (hr)= high transition rate equilibrium; **state change pattern**

**RESCORED DATA (Five-char)**	**Parsi**	**State for q=0.0001**	**State proba**	**State for q=0.001**	**State proba**	**State for q=0.01**	**State proba**	**State for q=0.1**	**State proba**
**Angiospermae**	free	free	1	free	1	free	0.98	equi (hr)	0.5
**Mesangiospermae**	free	free	1	free	1	free	0.99	equi (hr)	0.5
**Magnoliidae**	free	free	1	free	1	free	0.99	equi (hr)	0.5
**Monocotyledoneae**	free	free	1	free	1	free	0.99	equi (hr)	0.5
**Commelinidae**	free	free	1	free	1	free	0.95	equi (hr)	0.5
**Eudicotyledoneae**	free	free	1	free	1	free	0.99	equi (hr)	0.5
**Pentapetalae**	free, fused	**fused**	**0.63**	**free**	**0.71**	**free**	**0.89**	equi (hr)	0.5
**Superrosidae**	fused	**fused**	**0.64**	**free**	**0.72**	**free**	**0.9**	equi (hr)	0.5

**Rosidae**	fused	**fused**	**0.64**	**free**	**0.73**	**free**	**0.9**	equi (hr)	0.5
**Malvidae**	fused	**fused**	**0.53**	**free**	**0.84**	**free**	**0.68**	equi (hr)	0.5
**Fabidae**	free	free	1	free	1	free	0.95	equi (hr)	0.5
**Superasteridae**	free, fused	**fused**	**0.63**	**free**	**0.67**	**free**	**0.8**	equi (hr)	0.5
**Asteridae**	fused	fused	1	fused	0.97	fused	0.69	equi (hr)	0.5
**Campanulidae**	fused	fused	1	fused	1	fused	0.97	equi (hr)	0.5
**Lamiidae**	fused	fused	1	fused	1	fused	0.99	equi (hr)	0.5

**ORIGINAL DATA (Five-char)**	**Parsi**	**State for q=0.0001**	**State proba**	**State for q=0.001**	**State proba**	**State for q=0.01**	**State proba**	**State for q=0.1**	**State proba**
**Angiospermae**	free	free	1	free	1	free	0.98	equi (hr)	0.5
**Mesangiospermae**	free	free	1	free	1	free	1	equi (hr)	0.5
**Magnoliidae**	free	free	1	free	1	free	1	equi (hr)	0.5
**Monocotyledoneae**	free	free	1	free	1	free	1	equi (hr)	0.5
**Commelinidae**	free	free	1	free	1	free	0.98	equi (hr)	0.5
**Eudicotyledoneae**	free	free	1	free	1	free	1	equi (hr)	0.5
**Pentapetalae**	free	free	1	free	1	free	0.99	equi (hr)	0.5
**Superrosidae**	free	free	1	free	1	free	1	equi (hr)	0.5
**Rosidae**	free	free	1	free	1	free	1	equi (hr)	0.5
**Malvidae**	free	free	1	free	1	free	0.97	equi (hr)	0.5
**Fabidae**	free	free	1	free	1	free	1	equi (hr)	0.5
**Superasteridae**	free, fused	free	1	free	1	free	0.97	equi (hr)	0.5
**Asteridae**	free, fused	free	0.98	free	0.97	free	0.75	equi (hr)	0.5
**Campanulidae**	fused	fused	1	fused	1	fused	0.73	equi (hr)	0.5
**Lamiidae**	fused	fused	1	fused	1	fused	0.95	equi (hr)	0.5

**RESCORED DATA (PrunedMB)**	**Parsi**	**State for q=0.0001**	**State proba**	**State for q=0.001**	**State proba**	**State for q=0.01**	**State proba**	**State for q=0.1**	**State proba**
**Angiospermae**	free	free	1	free	1	free	0.98	equi (hr)	0.5
**Mesangiospermae**	free	free	1	free	1	free	1	equi (hr)	0.5
**Magnoliidae**	free	free	1	free	1	free	0.99	equi (hr)	0.5
**Monocotyledoneae**	free	free	1	free	1	free	0.99	equi (hr)	0.5
**Commelinidae**	free	free	1	free	1	free	0.96	equi (hr)	0.5
**Eudicotyledoneae**	free	free	1	free	1	free	0.99	equi (hr)	0.5

**Pentapetalae**	free, fused	free	0.98	free	0.95	free	0.95	equi (hr)	0.5
**Superrosidae**	free, fused	free	0.98	free	0.95	free	0.95	equi (hr)	0.5
**Rosidae**	free, fused	free	0.98	free	0.95	free	0.96	equi (hr)	0.5
**Malvidae**	free, fused	free	0.98	free	0.92	free	0.71	equi (hr)	0.5
**Fabidae**	free	free	1	free	1	free	0.99	equi (hr)	0.5
**Superasteridae**	free, fused	free	0.97	free	0.87	free	0.84	equi (hr)	0.5
**Asteridae**	fused	fused	1	fused	0.99	fused	0.78	equi (hr)	0.5
**Campanulidae**	fused	fused	1	fused	1	fused	0.99	equi (hr)	0.5
**Lamiidae**	fused	fused	1	fused	1	fused	1	equi (hr)	0.5

Phyllotaxis

q= transition rate (changes per Ma); Parsi= parsimony; State proba= state probability; equi= equivocal; (hr)= high transition rate equilibrium; whorl= whorled; spir= spiral

RESCORED DATA (Five-char)	Parsi	State for q=0.0001	State proba	State for q=0.001	State proba	State for q=0.01	State proba	State for q=0.1	State proba
**Angiospermae**	whorl, spir	whorl	0.98	whorl	0.98	whorl	0.96	equi (hr)	0.5
**Mesangiospermae**	whorl	whorl	1	whorl	1	whorl	1	equi (hr)	0.5
**Magnoliidae**	whorl	whorl	1	whorl	1	whorl	1	equi (hr)	0.5
**Monocotyledoneae**	whorl	whorl	1	whorl	1	whorl	1	equi (hr)	0.5
**Commelinidae**	whorl	whorl	1	whorl	1	whorl	1	equi (hr)	0.5
**Eudicotyledoneae**	whorl	whorl	1	whorl	1	whorl	1	equi (hr)	0.5
**Pentapetalae**	whorl	whorl	1	whorl	1	whorl	1	equi (hr)	0.5
**Superrosidae**	whorl	whorl	1	whorl	1	whorl	1	equi (hr)	0.5
**Rosidae**	whorl	whorl	1	whorl	1	whorl	1	equi (hr)	0.5
**Malvidae**	whorl	whorl	1	whorl	1	whorl	1	equi (hr)	0.5
**Fabidae**	whorl	whorl	1	whorl	1	whorl	1	equi (hr)	0.5
**Superasteridae**	whorl	whorl	1	whorl	1	whorl	1	equi (hr)	0.5
**Asteridae**	whorl	whorl	1	whorl	1	whorl	1	equi (hr)	0.5
**Campanulidae**	whorl	whorl	1	whorl	1	whorl	1	equi (hr)	0.5
**Lamiidae**	whorl	whorl	1	whorl	1	whorl	1	equi (hr)	0.5

ORIGINAL DATA (Five-char)	Parsi	State for q=0.0001	State proba	State for q=0.001	State proba	State for q=0.01	State proba	State for q=0.1	State proba

**Angiospermae**	whorl, spir	whorl	0.98	whorl	0.98	whorl	0.96	equi (hr)	0.5
**Mesangiospermae**	whorl	whorl	1	whorl	1	whorl	1	equi (hr)	0.5
**Magnoliidae**	whorl	whorl	1	whorl	1	whorl	1	equi (hr)	0.5
**Monocotyledoneae**	whorl	whorl	1	whorl	1	whorl	1	equi (hr)	0.5
**Commelinidae**	whorl	whorl	1	whorl	1	whorl	1	equi (hr)	0.5
**Eudicotyledoneae**	whorl	whorl	1	whorl	1	whorl	1	equi (hr)	0.5
**Pentapetalae**	whorl	whorl	1	whorl	1	whorl	1	equi (hr)	0.5
**Superrosidae**	whorl	whorl	1	whorl	1	whorl	1	equi (hr)	0.5
**Rosidae**	whorl	whorl	1	whorl	1	whorl	1	equi (hr)	0.5
**Malvidae**	whorl	whorl	1	whorl	1	whorl	1	equi (hr)	0.5
**Fabidae**	whorl	whorl	1	whorl	1	whorl	1	equi (hr)	0.5
**Superasteridae**	whorl	whorl	1	whorl	1	whorl	1	equi (hr)	0.5
**Asteridae**	whorl	whorl	1	whorl	1	whorl	1	equi (hr)	0.5
**Campanulidae**	whorl	whorl	1	whorl	1	whorl	1	equi (hr)	0.5
**Lamiidae**	whorl	whorl	1	whorl	1	whorl	1	equi (hr)	0.5

**RESCORED DATA (PrunedMB)**	**Parsi**	**State for q=0.0001**	**State proba**	**State for q=0.001**	**State proba**	**State for q=0.01**	**State proba**	**State for q=0.1**	**State proba**
**Angiospermae**	whorl. spir	whorl	0.98	whorl	0.98	whorl	0.96	equi (hr)	0.5
**Mesangiospermae**	whorl	whorl	1	whorl	1	whorl	1	equi (hr)	0.5
**Magnoliidae**	whorl	whorl	1	whorl	1	whorl	0.99	equi (hr)	0.5
**Monocotyledoneae**	whorl	whorl	1	whorl	1	whorl	1	equi (hr)	0.5
**Commelinidae**	whorl	whorl	1	whorl	1	whorl	1	equi (hr)	0.5
**Eudicotyledoneae**	whorl	whorl	1	whorl	1	whorl	1	equi (hr)	0.5
**Pentapetalae**	whorl	whorl	1	whorl	1	whorl	1	equi (hr)	0.5
**Superrosidae**	whorl	whorl	1	whorl	1	whorl	1	equi (hr)	0.5
**Rosidae**	whorl	whorl	1	whorl	1	whorl	1	equi (hr)	0.5
**Malvidae**	whorl	whorl	1	whorl	1	whorl	1	equi (hr)	0.5
**Fabidae**	whorl	whorl	1	whorl	1	whorl	1	equi (hr)	0.5
**Superasteridae**	whorl	whorl	1	whorl	1	whorl	1	equi (hr)	0.5
**Asteridae**	whorl	whorl	1	whorl	1	whorl	1	equi (hr)	0.5
**Campanulidae**	whorl	whorl	1	whorl	1	whorl	1	equi (hr)	0.5
**Lamiidae**	whorl	whorl	1	whorl	1	whorl	1	equi (hr)	0.5

RESCORED DATA (PrunedMB)	Parsi	State for q=0.0001	State proba	State for q=0.001	State proba	State for q=0.01	State proba	State for q=0.1	State proba
**Angiospermae**	whorl. spir	whorl	0.98	whorl	0.98	whorl	0.96	equi (hr)	0.5
**Mesangiospermae**	whorl	whorl	1	whorl	1	whorl	1	equi (hr)	0.5
**Magnoliidae**	whorl	whorl	1	whorl	1	whorl	0.99	equi (hr)	0.5
**Monocotyledoneae**	whorl	whorl	1	whorl	1	whorl	1	equi (hr)	0.5
**Commelinidae**	whorl	whorl	1	whorl	1	whorl	1	equi (hr)	0.5
**Eudicotyledoneae**	whorl	whorl	1	whorl	1	whorl	1	equi (hr)	0.5
**Pentapetalae**	whorl	whorl	1	whorl	1	whorl	1	equi (hr)	0.5
**Superrosidae**	whorl	whorl	1	whorl	1	whorl	1	equi (hr)	0.5
**Rosidae**	whorl	whorl	1	whorl	1	whorl	1	equi (hr)	0.5
**Malvidae**	whorl	whorl	1	whorl	1	whorl	1	equi (hr)	0.5
**Fabidae**	whorl	whorl	1	whorl	1	whorl	1	equi (hr)	0.5
**Superasteridae**	whorl	whorl	1	whorl	1	whorl	1	equi (hr)	0.5
**Asteridae**	whorl	whorl	1	whorl	1	whorl	1	equi (hr)	0.5
**Campanulidae**	whorl	whorl	1	whorl	1	whorl	1	equi (hr)	0.5
**Lamiidae**	whorl	whorl	1	whorl	1	whorl	1	equi (hr)	0.5

Merism
q= transition rate (changes per Ma); Parsi= parsimony; State proba= state probability; equi= equivocal; (hr)= high transition rate equilibrium; whorl= whorled; spir= spiral; penta= pentamerous; no-p= non-pentamerous

RESCORED DATA (Five-char)	Parsi	State for q=0.0001	State proba	State for q=0.001	State proba	State for q=0.01	State proba	State for q=0.1	State proba
**Angiospermae**	no-p	no-p	1	no-p	1	no-p	0.98	equi (hr)	0.5
**Mesangiospermae**	no-p	no-p	1	no-p	1	no-p	0.99	equi (hr)	0.5
**Magnoliidae**	no-p	no-p	1	no-p	1	no-p	0.99	equi (hr)	0.5
**Monocotyledoneae**	no-p	no-p	1	no-p	1	no-p	0.99	equi (hr)	0.5
**Commelinidae**	no-p	no-p	1	no-p	1	no-p	1	equi (hr)	0.5
**Eudicotyledoneae**	no-p	no-p	1	no-p	1	no-p	0.94	equi (hr)	0.5
**Pentapetalae**	penta	penta	1	penta	1	penta	0.98	equi (hr)	0.5
**Superrosidae**	penta	penta	1	penta	1	penta	0.99	equi (hr)	0.5
**Rosidae**	penta	penta	1	penta	1	penta	0.99	equi (hr)	0.5
**Malvidae**	penta	penta	1	penta	1	penta	0.99	equi (hr)	0.5
**Fabidae**	penta	penta	1	penta	1	penta	1	equi (hr)	0.5
**Superasteridae**	penta	penta	1	penta	1	penta	0.98	equi (hr)	0.5

**Asteridae**	penta	penta	1	penta	1	penta	1	equi (hr)	0.5
**Campanulidae**	penta	penta	1	penta	1	penta	1	equi (hr)	0.5
**Lamiidae**	penta	penta	1	penta	1	penta	1	equi (hr)	0.5

**ORIGINAL DATA (Five-char)**	**Parsi**	**State for q=0.0001**	**State proba**	**State for q=0.001**	**State proba**	**State for q=0.01**	**State proba**	**State for q=0.1**	**State proba**
**Angiospermae**	no-p	no-p	1	no-p	1	no-p	0.98	equi (hr)	0.5
**Mesangiospermae**	no-p	no-p	1	no-p	1	no-p	1	equi (hr)	0.5
**Magnoliidae**	no-p	no-p	1	no-p	1	no-p	1	equi (hr)	0.5
**Monocotyledoneae**	no-p	no-p	1	no-p	1	no-p	1	equi (hr)	0.5
**Commelinidae**	no-p	no-p	1	no-p	1	no-p	1	equi (hr)	0.5
**Eudicotyledoneae**	no-p	no-p	1	no-p	1	no-p	0.96	equi (hr)	0.5
**Pentapetalae**	no-p, penta	penta	1	penta	0.98	penta	0.87	equi (hr)	0.5
**Superrosidae**	penta	penta	1	penta	0.99	penta	0.9	equi (hr)	0.5
**Rosidae**	penta	penta	1	penta	1	penta	0.92	equi (hr)	0.5
**Malvidae**	penta	penta	1	penta	1	penta	0.96	equi (hr)	0.5
**Fabidae**	penta	penta	1	penta	1	penta	0.97	equi (hr)	0.5
**Superasteridae**	no-p, penta	penta	1	penta	0.98	penta	0.88	equi (hr)	0.5
**Asteridae**	penta	penta	1	penta	1	penta	0.98	equi (hr)	0.5
**Campanulidae**	penta	penta	1	penta	1	penta	0.99	equi (hr)	0.5
**Lamiidae**	penta	penta	1	penta	1	penta	0.99	equi (hr)	0.5

**RESCORED DATA (PrunedMB)**	**Parsi**	**State for q=0.0001**	**State proba**	**State for q=0.001**	**State proba**	**State for q=0.01**	**State proba**	**State for q=0.1**	**State proba**
**Angiospermae**	no-p	no-p	1	no-p	1	no-p	0.98	equi (hr)	0.5
**Mesangiospermae**	no-p	no-p	1	no-p	1	no-p	0.99	equi (hr)	0.5
**Magnoliidae**	no-p	no-p	1	no-p	1	no-p	1	equi (hr)	0.5
**Monocotyledoneae**	no-p	no-p	1	no-p	1	no-p	0.99	equi (hr)	0.5
**Commelinidae**	no-p	no-p	1	no-p	1	no-p	1	equi (hr)	0.5
**Eudicotyledoneae**	no-p	no-p	1	no-p	1	no-p	0.94	equi (hr)	0.5
**Pentapetalae**	penta	penta	1	penta	1	penta	0.98	equi (hr)	0.5
**Superrosidae**	penta	penta	1	penta	1	penta	0.99	equi (hr)	0.5
**Rosidae**	penta	penta	1	penta	1	penta	0.99	equi (hr)	0.5
**Malvidae**	penta	penta	1	penta	1	penta	0.99	equi (hr)	0.5
**Fabidae**	penta	penta	1	penta	1	penta	1	equi (hr)	0.5

**Superasteridae**	penta	penta	1	penta	1	penta	0.99	equi (hr)	0.5
**Asteridae**	penta	penta	1	penta	1	penta	1	equi (hr)	0.5
**Campanulidae**	penta	penta	1	penta	1	penta	1	equi (hr)	0.5
**Lamiidae**	penta	penta	1	penta	1	penta	0.99	equi (hr)	0.5

**RESCORED DATA (PrunedMB)**	**Parsi**	**State for q=0.0001**	**State proba**	**State for q=0.001**	**State proba**	**State for q=0.01**	**State proba**	**State for q=0.1**	**State proba**
**Angiospermae**	no-p	no-p	1	no-p	1	no-p	0.98	equi (hr)	0.5
**Mesangiospermae**	no-p	no-p	1	no-p	1	no-p	0.99	equi (hr)	0.5
**Magnoliidae**	no-p	no-p	1	no-p	1	no-p	1	equi (hr)	0.5
**Monocotyledoneae**	no-p	no-p	1	no-p	1	no-p	0.99	equi (hr)	0.5
**Commelinidae**	no-p	no-p	1	no-p	1	no-p	1	equi (hr)	0.5
**Eudicotyledoneae**	no-p	no-p	1	no-p	1	no-p	0.94	equi (hr)	0.5
**Pentapetalae**	penta	penta	1	penta	1	penta	0.98	equi (hr)	0.5
**Superrosidae**	penta	penta	1	penta	1	penta	0.99	equi (hr)	0.5
**Rosidae**	penta	penta	1	penta	1	penta	0.99	equi (hr)	0.5
**Malvidae**	penta	penta	1	penta	1	penta	0.99	equi (hr)	0.5
**Fabidae**	penta	penta	1	penta	1	penta	1	equi (hr)	0.5
**Superasteridae**	penta	penta	1	penta	1	penta	0.99	equi (hr)	0.5
**Asteridae**	penta	penta	1	penta	1	penta	1	equi (hr)	0.5
**Campanulidae**	penta	penta	1	penta	1	penta	1	equi (hr)	0.5
**Lamiidae**	penta	penta	1	penta	1	penta	0.99	equi (hr)	0.5

Differentiation

q= transition rate (changes per Ma); Parsi= parsimony; State proba= state probability; equi= equivocal; (hr)= high transition rate equilibrium; undif= undifferentiated; diff= differentiated; **state change pattern**; *non-recognized pattern*

**RESCORED DATA (Five-char)**	**Parsi**	**State for q=0.0001**	**State proba**	**State for q=0.001**	**State proba**	**State for q=0.01**	**State proba**	**State for q=0.1**	**State proba**
**Angiospermae**	undif	**undif**	**0.96**	**undif**	**0.57**	**diff**	**0.91**	equi (hr)	0.5
**Mesangiospermae**	undif	**undif**	**0.95**	**undif**	**0.53**	**diff**	**0.94**	equi (hr)	0.5
**Magnoliidae**	undif	**undif**	**0.95**	**undif**	**0.54**	**diff**	**0.92**	equi (hr)	0.5
**Monocotyledoneae**	undif	**undif**	**0.98**	**undif**	**0.66**	**diff**	**0.90**	equi (hr)	0.5
**Commelinidae**	undif, diff	*diff*	*0.74*	*diff*	*0.81*	*diff*	*0.85*	equi (hr)	0.5
**Eudicotyledoneae**	diff	diff	1	diff	1	diff	0.98	equi (hr)	0.5
**Pentapetalae**	diff	diff	1	diff	1	diff	1	equi (hr)	0.5

**Superrosidae**	diff	diff	1	diff	1	diff	1	equi (hr)	0.5
**Rosidae**	diff	diff	1	diff	1	diff	1	equi (hr)	0.5
**Malvidae**	diff	diff	1	diff	1	diff	1	equi (hr)	0.5
**Fabidae**	diff	diff	1	diff	1	diff	1	equi (hr)	0.5
**Superasteridae**	diff	diff	1	diff	1	diff	1	equi (hr)	0.5
**Asteridae**	diff	diff	1	diff	1	diff	1	equi (hr)	0.5
**Campanulidae**	diff	diff	1	diff	1	diff	1	equi (hr)	0.5
**Lamiidae**	diff	diff	1	diff	1	diff	1	equi (hr)	0.5

**ORIGINAL DATA (Five-char)**	**Parsi**	**State for q=0.0001**	**State proba**	**State for q=0.001**	**State proba**	**State for q=0.01**	**State proba**	**State for q=0.1**	**State proba**
**Angiospermae**	undif	**undif**	**0.98**	**undif**	**0.78**	**diff**	**0.87**	equi (hr)	0.5
**Mesangiospermae**	undif	**undif**	**0.98**	**undif**	**0.76**	**diff**	**0.91**	equi (hr)	0.5
**Magnoliidae**	undif	**undif**	**0.98**	**undif**	**0.76**	**diff**	**0.89**	equi (hr)	0.5
**Monocotyledoneae**	undif	**undif**	**0.99**	**undif**	**0.83**	**diff**	**0.87**	equi (hr)	0.5
**Commelinidae**	undif, diff	*diff*	*0.73*	*diff*	*0.73*	*diff*	*0.8*	equi (hr)	0.5
**Eudicotyledoneae**	diff	diff	1	diff	1	diff	0.97	equi (hr)	0.5
**Pentapetalae**	diff	diff	1	diff	1	diff	1	equi (hr)	0.5
**Superrosidae**	diff	diff	1	diff	1	diff	1	equi (hr)	0.5
**Rosidae**	diff	diff	1	diff	1	diff	1	equi (hr)	0.5
**Malvidae**	diff	diff	1	diff	1	diff	1	equi (hr)	0.5
**Fabidae**	diff	diff	1	diff	1	diff	1	equi (hr)	0.5
**Superasteridae**	diff	diff	1	diff	1	diff	1	equi (hr)	0.5
**Asteridae**	diff	diff	1	diff	1	diff	1	equi (hr)	0.5
**Campanulidae**	diff	diff	1	diff	1	diff	1	equi (hr)	0.5
**Lamiidae**	diff	diff	1	diff	1	diff	1	equi (hr)	0.5

**RESCORED DATA (PrunedMB)**	**Parsi**	**State for q=0.0001**	**State proba**	**State for q=0.001**	**State proba**	**State for q=0.01**	**State proba**	**State for q=0.1**	**State proba**
**Angiospermae**	undif	**diff**	**0.53**	**diff**	**0.72**	**diff**	**0.91**	equi (hr)	0.5
**Mesangiospermae**	undif	**diff**	**0.59**	**diff**	**0.78**	**diff**	**0.94**	equi (hr)	0.5
**Magnoliidae**	undif	**diff**	**0.66**	**diff**	**0.81**	**diff**	**0.93**	equi (hr)	0.5
**Monocotyledoneae**	undif	**undif**	**0.93**	**undif**	**0.63**	**diff**	**0.90**	equi (hr)	0.5
**Commelinidae**	diff	diff	1	diff	0.97	diff	0.87	equi (hr)	0.5
**Eudicotyledoneae**	diff	diff	1	diff	1	diff	0.98	equi (hr)	0.5

**Pentapetalae**	diff	diff	1	diff	1	diff	1	equi (hr)	0.5
**Superrosidae**	diff	diff	1	diff	1	diff	1	equi (hr)	0.5
**Rosidae**	diff	diff	1	diff	1	diff	1	equi (hr)	0.5
**Malvidae**	diff	diff	1	diff	1	diff	1	equi (hr)	0.5
**Fabidae**	diff	diff	1	diff	1	diff	1	equi (hr)	0.5
**Superasteridae**	diff	diff	1	diff	1	diff	1	equi (hr)	0.5
**Asteridae**	diff	diff	1	diff	1	diff	1	equi (hr)	0.5
**Campanulidae**	diff	diff	1	diff	1	diff	1	equi (hr)	0.5
**Lamiidae**	diff	diff	1	diff	1	diff	1	equi (hr)	0.5

Testing the impact of morphological rate heterogeneity on ancestral state reconstruction of five floral traits in angiosperms

Supplementary Tables 5: Parsimony and Mk1 imposed-rate ancestral states for the crown-group nodes of 54 botanical orders

Elisabeth Reyes, Sophie Nadot, Maria von Balthazar, Jürg Schönenberger, Hervé Sauquet

Supplementary Tables 5: Parsimony and Mk1 imposed-rate ancestral states for the crown-group nodes of 54 botanical orders

Five-char: Results for main tree

PrunedMB: Results for the pruned Smith & Brown ALLMB tree

Symmetry

q= transition rate (changes per Ma); Parsi= parsimony; State proba= state probability; equi= equivocal; (hr)= high transition rate equilibrium; actino= actinomorphic

RESCORED DATA (Five-char)	Parsi	State for q=0.0001	State proba	State for q=0.001	State proba	State for q=0.01	State proba	State for q=0.1	State proba
**Alismatales**	actino	actino	1	actino	1	actino	0.99	equi (hr)	0.5
**Apiales**	actino	actino	1	actino	1	actino	0.99	equi (hr)	0.5
**Aquifoliales**	actino	actino	1	actino	1	actino	0.99	equi (hr)	0.5
**Arecales**	actino	actino	1	actino	1	actino	0.94	equi (hr)	0.5
**Asparagales**	actino	actino	1	actino	1	actino	0.98	equi (hr)	0.5
**Asterales**	actino	actino	1	actino	1	actino	0.99	equi (hr)	0.5
**Austrobaileyales**	actino	actino	1	actino	1	actino	0.91	equi (hr)	0.5
**Berberidopsidales**	actino	actino	1	actino	1	actino	0.95	equi (hr)	0.5
**Boraginales**	actino	actino	1	actino	1	actino	0.96	equi (hr)	0.5
**Brassicales**	actino	actino	1	actino	1	actino	0.97	equi (hr)	0.5
**Bruniales**	actino	actino	1	actino	1	actino	0.94	equi (hr)	0.5
**Buxales**	actino	actino	1	actino	1	actino	0.84	equi (hr)	0.5
**Canellales**	actino	actino	1	actino	1	actino	0.97	equi (hr)	0.5
**Caryophyllales**	actino	actino	1	actino	1	actino	1	equi (hr)	0.5
**Celastrales**	actino	actino	1	actino	1	actino	0.95	equi (hr)	0.5
**Commelinales**	actino, zygo	zygo	0.89	zygo	0.85	zygo	0.61	equi (hr)	0.5
**Cornales**	actino	actino	1	actino	1	actino	0.99	equi (hr)	0.5
**Crossosomatales**	actino	actino	1	actino	1	actino	0.96	equi (hr)	0.5
**Cucurbitales**	actino	actino	1	actino	1	actino	0.98	equi (hr)	0.5
**Dioscoreales**	actino	actino	1	actino	1	actino	0.93	equi (hr)	0.5
**Dipsacales**	actino	actino	1	actino	0.97	actino	0.8	equi (hr)	0.5
**Ericales**	actino	actino	1	actino	1	actino	1	equi (hr)	0.5
**Escalloniales**	actino	actino	1	actino	1	actino	0.97	equi (hr)	0.5
**Fabales**	actino	actino	0.99	actino	0.93	actino	0.81	equi (hr)	0.5
**Fagales**	actino	actino	1	actino	1	actino	0.99	equi (hr)	0.5
**Garryales**	actino	actino	1	actino	1	actino	0.99	actino (hr)	0.51
**Gentianales**	actino	actino	1	actino	1	actino	0.98	equi (hr)	0.5

**Geraniales**	actino	actino	1	actino	0.99	actino	0.92	equi (hr)	0.5
**Huerteales**	actino	actino	1	actino	1	actino	0.97	equi (hr)	0.5
**Icacinales**	actino	actino	1	actino	1	actino	0.93	equi (hr)	0.5
**Lamiales**	actino, zygo	actino	0.73	actino	0.73	actino	0.72	equi (hr)	0.5
**Laurales**	actino	actino	1	actino	1	actino	0.99	equi (hr)	0.5
**Liliales**	actino	actino	1	actino	1	actino	0.95	equi (hr)	0.5
**Magnoliales**	actino	actino	1	actino	1	actino	0.98	equi (hr)	0.5
**Malpighiales**	actino	actino	1	actino	1	actino	1	equi (hr)	0.5
**Malvales**	actino	actino	1	actino	1	actino	0.99	equi (hr)	0.5
**Myrtales**	actino	actino	1	actino	1	actino	0.99	equi (hr)	0.5
**Nymphaeales**	actino	actino	1	actino	1	actino	0.9	equi (hr)	0.5
**Oxalidales**	actino	actino	1	actino	1	actino	0.99	equi (hr)	0.5
**Pandanales**	actino	actino	1	actino	1	actino	0.98	equi (hr)	0.5
**Paracryphiales**	actino	actino	1	actino	1	actino	0.92	equi (hr)	0.5
**Petrosaviales**	actino	actino	1	actino	1	actino	0.87	equi (hr)	0.5
**Piperales**	actino	actino	1	actino	1	actino	0.95	equi (hr)	0.5
**Poales**	actino	actino	1	actino	1	actino	0.96	equi (hr)	0.5
**Proteales**	actino	actino	1	actino	1	actino	0.99	equi (hr)	0.5
**Ranunculales**	actino	actino	1	actino	1	actino	1	equi (hr)	0.5
**Rosales**	actino	actino	1	actino	1	actino	0.99	equi (hr)	0.5
**Santalales**	actino	actino	1	actino	1	actino	0.99	equi (hr)	0.5
**Sapindales**	actino	actino	1	actino	1	actino	0.99	equi (hr)	0.5
**Saxifragales**	actino	actino	1	actino	1	actino	1	equi (hr)	0.5
**Solanales**	actino	actino	1	actino	1	actino	0.95	equi (hr)	0.5
**Vitales**	actino	actino	1	actino	1	actino	0.87	equi (hr)	0.5
**Zingiberales**	zygo	zygo	1	zygo	1	zygo	0.96	equi (hr)	0.5
**Zygophyllales**	actino	actino	1	actino	0.96	actino	0.73	equi (hr)	0.5

**ORIGINAL DATA (Five-char)**	**Parsi**	**State for q=0.0001**	**State proba**	**State for q=0.001**	**State proba**	**State for q=0.01**	**State proba**	**State for q=0.1**	**State proba**
**Alismatales**	actino	actino	1	actino	1	actino	0.99	equi (hr)	0.5
**Apiales**	actino	actino	1	actino	1	actino	0.99	equi (hr)	0.5
**Aquifoliales**	actino	actino	1	actino	1	actino	0.99	equi (hr)	0.5
**Arecales**	actino	actino	1	actino	1	actino	0.94	equi (hr)	0.5
**Asparagales**	actino	actino	1	actino	1	actino	0.99	equi (hr)	0.5

**Asterales**	actino	actino	1	actino	1	actino	0.99	equi (hr)	0.5
**Austrobaileyales**	actino	actino	1	actino	1	actino	0.91	equi (hr)	0.5
**Berberidopsidales**	actino	actino	1	actino	1	actino	0.95	equi (hr)	0.5
**Boraginales**	actino	actino	1	actino	1	actino	0.96	equi (hr)	0.5
**Brassicales**	actino	actino	1	actino	1	actino	0.97	equi (hr)	0.5
**Bruniales**	actino	actino	1	actino	1	actino	0.94	equi (hr)	0.5
**Buxales**	actino	actino	1	actino	1	actino	0.84	equi (hr)	0.5
**Canellales**	actino	actino	1	actino	1	actino	0.97	equi (hr)	0.5
**Caryophyllales**	actino	actino	1	actino	1	actino	1	equi (hr)	0.5
**Celastrales**	actino	actino	1	actino	1	actino	0.95	equi (hr)	0.5
**Commelinales**	actino, zygo	zygo	0.52	equi	0.5	actino	0.51	equi (hr)	0.5
**Cornales**	actino	actino	1	actino	1	actino	0.99	equi (hr)	0.5
**Crossosomatales**	actino	actino	1	actino	1	actino	0.96	equi (hr)	0.5
**Cucurbitales**	actino	actino	1	actino	1	actino	0.98	equi (hr)	0.5
**Dioscoreales**	actino	actino	1	actino	1	actino	0.93	equi (hr)	0.5
**Dipsacales**	actino	actino	1	actino	0.97	actino	0.8	equi (hr)	0.5
**Ericales**	actino	actino	1	actino	1	actino	1	equi (hr)	0.5
**Escalloniales**	actino	actino	1	actino	1	actino	0.97	equi (hr)	0.5
**Fabales**	actino	actino	0.99	actino	0.93	actino	0.81	equi (hr)	0.5
**Fagales**	actino	actino	1	actino	1	actino	0.99	equi (hr)	0.5
**Garryales**	actino	actino	1	actino	1	actino	0.99	actino (hr)	0.51
**Gentianales**	actino	actino	1	actino	1	actino	0.98	equi (hr)	0.5
**Geraniales**	actino	actino	1	actino	1	actino	0.94	equi (hr)	0.5
**Huerteales**	actino	actino	1	actino	1	actino	0.97	equi (hr)	0.5
**Icacinales**	actino	actino	1	actino	1	actino	0.93	equi (hr)	0.5
**Lamiales**	actino, zygo	actino	0.73	actino	0.73	actino	0.71	equi (hr)	0.5
**Laurales**	actino	actino	1	actino	1	actino	0.99	equi (hr)	0.5
**Liliales**	actino	actino	1	actino	1	actino	0.95	equi (hr)	0.5
**Magnoliales**	actino	actino	1	actino	1	actino	0.98	equi (hr)	0.5
**Malpighiales**	actino	actino	1	actino	1	actino	1	equi (hr)	0.5
**Malvales**	actino	actino	1	actino	1	actino	0.99	equi (hr)	0.5
**Myrtales**	actino	actino	1	actino	1	actino	0.99	equi (hr)	0.5
**Nymphaeales**	actino	actino	1	actino	1	actino	0.9	equi (hr)	0.5

**Oxalidales**	actino	actino	1	actino	1	actino	0.99	equi (hr)	0.5
**Pandanales**	actino	actino	1	actino	1	actino	0.98	equi (hr)	0.5
**Paracryphiales**	actino	actino	1	actino	1	actino	0.92	equi (hr)	0.5
**Petrosaviales**	actino	actino	1	actino	1	actino	0.87	equi (hr)	0.5
**Piperales**	actino	actino	1	actino	1	actino	0.95	equi (hr)	0.5
**Poales**	actino	actino	1	actino	1	actino	0.96	equi (hr)	0.5
**Proteales**	actino	actino	1	actino	1	actino	0.99	equi (hr)	0.5
**Ranunculales**	actino	actino	1	actino	1	actino	1	equi (hr)	0.5
**Rosales**	actino	actino	1	actino	1	actino	0.99	equi (hr)	0.5
**Santalales**	actino	actino	1	actino	1	actino	0.99	equi (hr)	0.5
**Sapindales**	actino	actino	1	actino	1	actino	0.99	equi (hr)	0.5
**Saxifragales**	actino	actino	1	actino	1	actino	1	equi (hr)	0.5
**Solanales**	actino	actino	1	actino	1	actino	0.96	equi (hr)	0.5
**Vitales**	actino	actino	1	actino	1	actino	0.87	equi (hr)	0.5
**Zingiberales**	zygo	zygo	1	zygo	1	zygo	0.95	equi (hr)	0.5
**Zygophyllales**	actino	actino	1	actino	0.96	actino	0.73	equi (hr)	0.5

**RESCORED DATA (PrunedMB)**	**Parsi**	**State for q=0.0001**	**State proba**	**State for q=0.001**	**State proba**	**State for q=0.01**	**State proba**	**State for q=0.1**	**State proba**
**Alismatales**	actino	actino	1	actino	1	actino	0.99	equi (hr)	0.5
**Apiales**	actino	actino	1	actino	1	actino	0.99	equi (hr)	0.5
**Aquifoliales**	actino	actino	1	actino	1	actino	0.99	equi (hr)	0.5
**Arecales**	actino	actino	1	actino	1	actino	0.91	equi (hr)	0.5
**Asparagales**	actino	actino	1	actino	1	actino	0.98	equi (hr)	0.5
**Asterales**	actino	actino	1	actino	1	actino	0.99	equi (hr)	0.5
**Austrobaileyales**	actino	actino	1	actino	1	actino	0.91	equi (hr)	0.5
**Berberidopsidales**	actino	actino	1	actino	1	actino	0.95	equi (hr)	0.5
**Boraginales**	actino	actino	1	actino	1	actino	0.96	equi (hr)	0.5
**Brassicales**	actino	actino	1	actino	1	actino	0.97	equi (hr)	0.5
**Bruniales**	actino	actino	1	actino	1	actino	0.94	equi (hr)	0.5
**Buxales**	actino	actino	1	actino	1	actino	0.92	equi (hr)	0.5
**Canellales**	actino	actino	1	actino	1	actino	0.97	equi (hr)	0.5
**Caryophyllales**	actino	actino	1	actino	1	actino	1	equi (hr)	0.5
**Celastrales**	actino	actino	1	actino	1	actino	0.92	equi (hr)	0.5
**Commelinales**	actino, zygo	zygo	0.81	zygo	0.74	zygo	0.54	equi (hr)	0.5

**Cornales**	actino	actino	1	actino	1	actino	0.99	equi (hr)	0.5
**Crossosomatales**	actino	actino	1	actino	1	actino	0.96	equi (hr)	0.5
**Cucurbitales**	actino	actino	1	actino	1	actino	0.98	equi (hr)	0.5
**Dioscoreales**	actino	actino	1	actino	1	actino	0.92	equi (hr)	0.5
**Dipsacales**	actino	actino	1	actino	0.97	actino	0.78	equi (hr)	0.5
**Ericales**	actino	actino	1	actino	1	actino	1	equi (hr)	0.5
**Escalloniales**	actino	actino	1	actino	1	actino	0.99	equi (hr)	0.5
**Fabales**	actino	actino	1	actino	0.97	actino	0.84	equi (hr)	0.5
**Fagales**	actino	actino	1	actino	1	actino	0.98	equi (hr)	0.5
**Garryales**	actino	actino	1	actino	1	actino	0.99	actino (hr)	0.51
**Gentianales**	actino	actino	1	actino	1	actino	0.99	equi (hr)	0.5
**Geraniales**	actino	actino	1	actino	1	actino	0.87	equi (hr)	0.5
**Huerteales**	actino	actino	1	actino	1	actino	0.95	equi (hr)	0.5
**Icacinales**	actino	actino	1	actino	1	actino	0.98	equi (hr)	0.5
**Lamiales**	actino	actino	1	actino	0.99	actino	0.81	equi (hr)	0.5
**Laurales**	actino	actino	1	actino	1	actino	0.99	equi (hr)	0.5
**Liliales**	actino	actino	1	actino	1	actino	0.96	equi (hr)	0.5
**Magnoliales**	actino	actino	1	actino	1	actino	0.99	equi (hr)	0.5
**Malpighiales**	actino	actino	1	actino	1	actino	1	equi (hr)	0.5
**Malvales**	actino	actino	1	actino	1	actino	0.99	equi (hr)	0.5
**Myrtales**	actino	actino	1	actino	1	actino	0.99	equi (hr)	0.5
**Nymphaeales**	actino	actino	1	actino	1	actino	0.9	equi (hr)	0.5
**Oxalidales**	actino	actino	1	actino	1	actino	0.99	equi (hr)	0.5
**Pandanales**	actino	actino	1	actino	1	actino	0.95	equi (hr)	0.5
**Paracryphiales**	actino	actino	1	actino	1	actino	0.92	equi (hr)	0.5
**Petrosaviales**	actino	actino	1	actino	1	actino	0.87	equi (hr)	0.5
**Piperales**	actino	actino	1	actino	1	actino	0.93	equi (hr)	0.5
**Poales**	actino	actino	1	actino	1	actino	0.97	equi (hr)	0.5
**Proteales**	actino	actino	1	actino	1	actino	0.99	equi (hr)	0.5
**Ranunculales**	actino	actino	1	actino	1	actino	0.99	equi (hr)	0.5
**Rosales**	actino	actino	1	actino	1	actino	0.99	equi (hr)	0.5
**Santalales**	actino	actino	1	actino	1	actino	0.99	equi (hr)	0.5
**Sapindales**	actino	actino	1	actino	1	actino	1	equi (hr)	0.5
**Saxifragales**	actino	actino	1	actino	1	actino	1	equi (hr)	0.5

**Solanales**	actino	actino	1	actino	1	actino	0.98	equi (hr)	0.5
**Vitales**	actino	actino	1	actino	1	actino	0.87	equi (hr)	0.5
**Zingiberales**	zygo	zygo	1	zygo	1	zygo	0.06	equi (hr)	0.5
**Zygophyllales**	actino	actino	1	actino	0.96	actino	0.89	equi (hr)	0.5

Fusion

q= transition rate (changes per Ma); Parsi= parsimony; State proba= state probability; equi= equivocal; (hr)= high transition rate equilibrium; **state change pattern**; *non-recognized pattern*

RESCORED DATA (Five-char)	Parsi	State for q=0.0001	State proba	State for q=0.001	State proba	State for q=0.01	State proba	State for q=0.1	State proba
**Alismatales**	free	free	1	free	1	free	0.99	equi (hr)	0.5
**Apiales**	free, fused	fused	0.97	fused	0.97	fused	0.93	equi (hr)	0.5
**Aquifoliales**	fused	fused	1	fused	1	fused	0.91	equi (hr)	0.5
**Arecales**	free	free	1	free	1	free	0.81	equi (hr)	0.5
**Asparagales**	free	free	1	free	1	free	0.96	equi (hr)	0.5
**Asterales**	fused	fused	1	fused	1	fused	1	equi (hr)	0.5
**Austrobaileyales**	free	free	1	free	1	free	0.91	equi (hr)	0.5
**Berberidopsidales**	free	free	1	free	0.99	free	0.94	equi (hr)	0.5
**Boraginales**	fused	fused	1	fused	1	fused	0.99	equi (hr)	0.5
**Brassicales**	free, fused	fused	1	fused	0.95	fused	0.69	equi (hr)	0.5
**Bruniales**	free, fused	fused	1	fused	0.99	fused	0.92	equi (hr)	0.5
**Buxales**	free	free	1	free	1	free	0.81	equi (hr)	0.5
**Canellales**	free, fused	free	0.92	free	0.92	free	0.91	equi (hr)	0.5
**Caryophyllales**	fused	**fused**	**1**	**fused**	**0.93**	**free**	**0.63**	equi (hr)	0.5
**Celastrales**	free	free	1	free	1	free	0.92	equi (hr)	0.5
**Commelinales**	free, fused	free	1	free	0.99	free	0.87	equi (hr)	0.5
**Cornales**	fused	fused	1	fused	0.96	fused	0.65	equi (hr)	0.5
**Crossosomatales**	fused	**fused**	**0.6**	**free**	**0.73**	**free**	**0.7**	equi (hr)	0.5
**Cucurbitales**	free	free	1	free	1	free	0.78	equi (hr)	0.5
**Dioscoreales**	free	free	0.99	free	0.9	free	0.69	equi (hr)	0.5
**Dipsacales**	fused	fused	1	fused	1	fused	0.96	equi (hr)	0.5
**Ericales**	fused	fused	1	fused	1	fused	0.95	equi (hr)	0.5
**Escalloniales**	fused	fused	1	fused	1	fused	0.96	equi (hr)	0.5

**Fabales**	free	free	1	free	0.99	free	0.80	equi (hr)	0.5
**Fagales**	free, fused	free	0.97	free	0.97	free	0.89	equi (hr)	0.5
**Garryales**	free, fused	free	1	free	0.98	free	0.83	free (hr)	0.51
**Gentianales**	fused	fused	1	fused	1	fused	0.98	equi (hr)	0.5
**Geraniales**	fused	**fused**	**0.53**	**free**	**0.89**	**free**	**0.92**	equi (hr)	0.5
**Huerteales**	free, fused	**fused**	**0.59**	**free**	**0.73**	**free**	**0.70**	equi (hr)	0.5
**Icacinales**	fused	fused	1	fused	1	fused	0.91	equi (hr)	0.5
**Lamiales**	fused	fused	1	fused	1	fused	0.99	equi (hr)	0.5
**Laurales**	free	free	1	free	1	free	0.95	equi (hr)	0.5
**Liliales**	free	free	1	free	1	free	0.96	equi (hr)	0.5
**Magnoliales**	free	free	1	free	1	free	0.95	equi (hr)	0.5
**Malpighiales**	free	free	1	free	1	free	0.94	equi (hr)	0.5
**Malvales**	free, fused	fused	1	fused	1	fused	0.85	equi (hr)	0.5
**Myrtales**	fused	**fused**	**0.54**	**free**	**0.86**	**free**	**0.8**	equi (hr)	0.5
**Nymphaeales**	free	free	1	free	1	free	0.89	equi (hr)	0.5
**Oxalidales**	free	free	1	free	0.97	free	0.88	equi (hr)	0.5
**Pandanales**	free	free	0.99	free	0.95	free	0.74	equi (hr)	0.5
**Paracryphiales**	free	free	0.99	free	0.91	free	0.56	equi (hr)	0.5
**Petrosaviales**	free	free	1	free	0.97	free	0.79	equi (hr)	0.5
**Piperales**	free, fused	free	0.96	free	0.94	free	0.8	equi (hr)	0.5
**Poales**	free, fused	free	0.98	free	0.98	free	0.91	equi (hr)	0.5
**Proteales**	free	free	1	free	1	free	0.96	equi (hr)	0.5
**Ranunculales**	free	free	1	free	1	free	0.98	equi (hr)	0.5
**Rosales**	free	free	1	free	1	free	0.88	equi (hr)	0.5
**Santalales**	fused	fused	1	fused	0.99	fused	0.72	equi (hr)	0.5
**Sapindales**	free, fused	**free**	**0.52**	**free**	**0.85**	**free**	**0.63**	equi (hr)	0.5
**Saxifragales**	fused	**fused**	**0.89**	**fused**	**0.76**	**free**	**0.66**	equi (hr)	0.5
**Solanales**	fused	fused	1	fused	1	fused	0.99	equi (hr)	0.5
**Vitales**	fused	fused	1	fused	0.96	fused	0.69	equi (hr)	0.5
**Zingiberales**	fused	fused	1	fused	1	fused	0.93	equi (hr)	0.5
**Zygophyllales**	free	free	1	free	1	free	1	free (hr)	0.6

ORIGINAL DATA (Five-char)	Parsi	State for q=0.0001	State proba	State for q=0.001	State proba	State for q=0.01	State proba	State for q=0.1	State proba
**Alismatales**	free	free	1	free	1	free	0.99	equi (hr)	0.5
**Apiales**	free	free	0.99	free	0.89	free	0.52	equi (hr)	0.5
**Aquifoliales**	fused	fused	1	fused	0.99	fused	0.71	equi (hr)	0.5
**Arecales**	free	free	1	free	1	free	0.87	equi (hr)	0.5
**Asparagales**	free	free	1	free	1	free	0.97	equi (hr)	0.5
**Asterales**	fused	fused	1	fused	1	fused	0.92	equi (hr)	0.5
**Austrobaileyales**	free	free	1	free	1	free	0.92	equi (hr)	0.5
**Berberidopsidales**	free	free	1	free	1	free	0.95	equi (hr)	0.5
**Boraginales**	fused	fused	1	fused	1	fused	0.98	equi (hr)	0.5
**Brassicales**	free	free	1	free	1	free	0.92	equi (hr)	0.5
**Bruniales**	free, fused	fused	0.89	fused	0.89	fused	0.70	equi (hr)	0.5
**Buxales**	free	free	1	free	1	free	0.84	equi (hr)	0.5
**Canellales**	free	free	0.98	free	1	free	0.95	equi (hr)	0.5
**Caryophyllales**	free, fused	free	0.97	free	0.97	free	0.89	equi (hr)	0.5
**Celastrales**	free	free	1	free	1	free	0.95	equi (hr)	0.5
**Commelinales**	free	free	1	free	0.99	free	0.93	equi (hr)	0.5
**Cornales**	free	free	1	free	1	free	0.78	equi (hr)	0.5
**Crossosomatales**	free, fused	free	0.85	free	0.84	free	0.78	equi (hr)	0.5
**Cucurbitales**	free	free	1	free	1	free	0.8	equi (hr)	0.5
**Dioscoreales**	free	free	0.99	free	0.9	free	0.7	equi (hr)	0.5
**Dipsacales**	fused	fused	1	fused	0.98	fused	0.83	equi (hr)	0.5
**Ericales**	free, fused	fused	0.99	fused	0.9	fused	0.63	equi (hr)	0.5
**Escalloniales**	fused	fused	1	fused	1	fused	0.78	equi (hr)	0.5
**Fabales**	free	free	1	free	1	free	0.89	equi (hr)	0.5
**Fagales**	free	free	1	free	1	free	0.95	equi (hr)	0.5
**Garryales**	free, fused	free	1	free	0.98	free	0.87	free (hr)	0.51
**Gentianales**	fused	fused	1	fused	1	fused	0.96	equi (hr)	0.5
**Geraniales**	free, fused	free	1	free	1	free	0.97	equi (hr)	0.5
**Huerteales**	free	free	1	free	0.99	free	0.89	equi (hr)	0.5
**Icacinales**	fused	fused	1	fused	0.99	fused	0.7	equi (hr)	0.5

**Lamiales**	fused	fused	1	fused	1	fused	0.99	equi (hr)	0.5
**Laurales**	free	free	1	free	1	free	0.96	equi (hr)	0.5
**Liliales**	free	free	1	free	1	free	0.97	equi (hr)	0.5
**Magnoliales**	free	free	1	free	1	free	0.96	equi (hr)	0.5
**Malpighiales**	free	free	1	free	1	free	1	equi (hr)	0.5
**Malvales**	free, fused	fused	0.99	fused	0.91	fused	0.57	equi (hr)	0.5
**Myrtales**	free, fused	free	0.98	free	0.98	free	0.88	equi (hr)	0.5
**Nymphaeales**	free	free	1	free	1	free	0.9	equi (hr)	0.5
**Oxalidales**	free	free	1	free	0.97	free	0.93	equi (hr)	0.5
**Pandanales**	free	free	0.99	free	0.95	free	0.75	equi (hr)	0.5
**Paracryphiales**	free	free	0.99	free	0.93	free	0.66	equi (hr)	0.5
**Petrosaviales**	free	free	1	free	0.97	free	0.79	equi (hr)	0.5
**Piperales**	free, fused	free	1	free	1	free	0.86	equi (hr)	0.5
**Poales**	free	free	1	free	1	free	0.97	equi (hr)	0.5
**Proteales**	free	free	1	free	1	free	0.99	equi (hr)	0.5
**Ranunculales**	free	free	1	free	1	free	1	equi (hr)	0.5
**Rosales**	free	free	1	free	1	free	0.98	equi (hr)	0.5
**Santalales**	fused	**fused**	**1**	**fused**	**0.97**	**free**	**0.52**	equi (hr)	0.5
**Sapindales**	free	free	1	free	1	free	0.96	equi (hr)	0.5
**Saxifragales**	free, fused	free	1	free	0.98	free	0.9	equi (hr)	0.5
**Solanales**	fused	fused	1	fused	1	fused	0.97	equi (hr)	0.5
**Vitales**	free, fused	fused	0.52	fused	0.52	fused	0.52	equi (hr)	0.5
**Zingiberales**	free, fused	fused	0.67	fused	0.65	fused	0.54	equi (hr)	0.5
**Zygophyllales**	free	free	1	free	1	free	1	free (hr)	0.6

**RESCORED DATA (PrunedMB)**	**Parsi**	**State for q=0.0001**	**State proba**	**State for q=0.001**	**State proba**	**State for q=0.01**	**State proba**	**State for q=0.1**	**State proba**
**Alismatales**	free	free	1	free	1	free	0.99	equi (hr)	0.5
**Apiales**	free, fused	fused	0.97	fused	0.97	fused	0.94	equi (hr)	0.5
**Aquifoliales**	fused	fused	1	fused	1	fused	0.88	equi (hr)	0.5
**Arecales**	free	free	1	free	1	free	0.92	equi (hr)	0.5
**Asparagales**	free	free	1	free	1	free	0.96	equi (hr)	0.5

**Asterales**	fused	fused	1	fused	1	fused	1	equi (hr)	0.5
**Austrobaileyales**	free	free	1	free	1	free	0.91	equi (hr)	0.5
**Berberidopsidales**	free	free	1	free	0.99	free	0.94	equi (hr)	0.5
**Boraginales**	fused	fused	1	fused	1	fused	0.97	equi (hr)	0.5
**Brassicales**	fused	fused	0.98	fused	0.88	fused	0.64	equi (hr)	0.5
**Bruniales**	free, fused	fused	0.99	fused	0.99	fused	0.92	equi (hr)	0.5
**Buxales**	free	free	1	free	1	free	0.92	equi (hr)	0.5
**Canellales**	fused	**fused**	**0.86**	**free**	**0.63**	**free**	**0.9**	equi (hr)	0.5
**Caryophyllales**	fused	**fused**	**0.98**	**fused**	**0.83**	**free**	**0.74**	equi (hr)	0.5
**Celastrales**	free	free	1	free	1	free	0.91	equi (hr)	0.5
**Commelinales**	free	free	1	free	1	free	0.98	equi (hr)	0.5
**Cornales**	fused	fused	1	fused	0.99	fused	0.77	equi (hr)	0.5
**Crossosomatales**	free, fused	free	0.83	free	0.81	free	0.72	equi (hr)	0.5
**Cucurbitales**	free	free	1	free	0.99	free	0.69	equi (hr)	0.5
**Dioscoreales**	free	free	0.99	free	0.88	free	0.66	equi (hr)	0.5
**Dipsacales**	fused	fused	1	fused	1	fused	0.97	equi (hr)	0.5
**Ericales**	fused	fused	1	fused	1	fused	0.96	equi (hr)	0.5
**Escalloniales**	fused	fused	1	fused	1	fused	0.99	equi (hr)	0.5
**Fabales**	free	free	1	free	0.99	free	0.85	equi (hr)	0.5
**Fagales**	free, fused	free	0.97	free	0.97	free	0.91	equi (hr)	0.5
**Garryales**	free, fused	free	1	free	0.98	free	0.84	free (hr)	0.51
**Gentianales**	fused	fused	1	fused	1	fused	0.99	equi (hr)	0.5
**Geraniales**	free, fused	free	0.98	free	0.98	free	0.96	equi (hr)	0.5
**Huerteales**	free, fused	free	0.84	free	0.81	free	0.78	equi (hr)	0.5
**Icacinales**	fused	fused	1	fused	1	fused	0.98	equi (hr)	0.5
**Lamiales**	fused	fused	1	fused	1	fused	0.99	equi (hr)	0.5
**Laurales**	free	free	1	free	1	free	0.93	equi (hr)	0.5
**Liliales**	free	free	1	free	1	free	0.97	equi (hr)	0.5
**Magnoliales**	free	free	1	free	1	free	0.98	equi (hr)	0.5
**Malpighiales**	fused	**fused**	**0.51**	**free**	**0.92**	**free**	**0.97**	equi (hr)	0.5
**Malvales**	fused	fused	1	fused	1	fused	0.9	equi (hr)	0.5

**Myrtales**	free, fused	free	0.98	free	0.97	free	0.87	equi (hr)	0.5
**Nymphaeales**	free	free	1	free	1	free	0.89	equi (hr)	0.5
**Oxalidales**	free, fused	*free*	*0.82*	*free*	*0.94*	*free*	*0.89*	equi (hr)	0.5
**Pandanales**	free	free	0.99	free	0.94	free	0.78	equi (hr)	0.5
**Paracryphiales**	free	free	0.99	free	0.91	free	0.56	equi (hr)	0.5
**Petrosaviales**	free	free	1	free	0.97	free	0.79	equi (hr)	0.5
**Piperales**	free, fused	*free*	*0.67*	*free*	*0.84*	*free*	*0.81*	equi (hr)	0.5
**Poales**	free, fused	free	0.99	free	0.98	free	0.93	equi (hr)	0.5
**Proteales**	free	free	1	free	1	free	0.97	equi (hr)	0.5
**Ranunculales**	free	free	1	free	1	free	0.98	equi (hr)	0.5
**Rosales**	free	free	1	free	1	free	0.85	equi (hr)	0.5
**Santalales**	fused	fused	1	fused	0.99	fused	0.77	equi (hr)	0.5
**Sapindales**	free, fused	free	0.98	free	0.92	free	0.56	equi (hr)	0.5
**Saxifragales**	free, fused	free	0.98	free	0.95	free	0.93	equi (hr)	0.5
**Solanales**	fused	fused	1	fused	1	fused	1	equi (hr)	0.5
**Vitales**	fused	fused	0.99	fused	0.95	fused	0.68	equi (hr)	0.5
**Zingiberales**	fused	fused	1	fused	0.99	fused	0.88	equi (hr)	0.5
**Zygophyllales**	free	free	1	free	1	free	0.99	equi (hr)	0.5

Phyllotaxis

q= transition rate (changes per Ma); Parsi= parsimony; State proba= state probability; equi= equivocal; (hr)= high transition rate equilibrium; whorl= whorled; spir= spiral

RESCORED DATA (Five-char)	Parsi	State for q=0.0001	State proba	State for q=0.001	State proba	State for q=0.01	State proba	State for q=0.1	State proba
**Alismatales**	whorl	whorl	1	whorl	1	whorl	0.99	equi (hr)	0.5
**Apiales**	whorl	whorl	1	whorl	1	whorl	1	equi (hr)	0.5
**Aquifoliales**	whorl	whorl	1	whorl	1	whorl	0.98	equi (hr)	0.5
**Arecales**	whorl	whorl	1	whorl	1	whorl	0.94	equi (hr)	0.5
**Asparagales**	whorl	whorl	1	whorl	1	whorl	1	equi (hr)	0.5
**Asterales**	whorl	whorl	1	whorl	1	whorl	1	equi (hr)	0.5
**Austrobaileyales**	spir	**spir**	**0.99**	**spir**	**0.84**	**whorl**	**0.78**	equi (hr)	0.5
**Berberidopsidales**	whorl	whorl	0.99	whorl	0.92	whorl	0.58	equi (hr)	0.5
**Boraginales**	whorl	whorl	1	whorl	1	whorl	0.99	equi (hr)	0.5

**Brassicales**	whorl	whorl	1	whorl	1	whorl	1	equi (hr)	0.5
**Bruniales**	whorl	whorl	1	whorl	1	whorl	0.97	equi (hr)	0.5
**Buxales**	whorl	whorl	1	whorl	1	whorl	0.78	equi (hr)	0.5
**Canellales**	whorl	whorl	1	whorl	1	whorl	0.96	equi (hr)	0.5
**Caryophyllales**	whorl	whorl	1	whorl	1	whorl	1	equi (hr)	0.5
**Celastrales**	whorl	whorl	1	whorl	1	whorl	0.95	equi (hr)	0.5
**Commelinales**	whorl	whorl	1	whorl	1	whorl	1	equi (hr)	0.5
**Cornales**	whorl	whorl	1	whorl	1	whorl	0.99	equi (hr)	0.5
**Crossosomatales**	whorl	whorl	1	whorl	1	whorl	0.94	equi (hr)	0.5
**Cucurbitales**	whorl	whorl	1	whorl	1	whorl	0.99	equi (hr)	0.5
**Dioscoreales**	whorl	whorl	1	whorl	1	whorl	0.96	equi (hr)	0.5
**Dipsacales**	whorl	whorl	1	whorl	1	whorl	0.99	equi (hr)	0.5
**Ericales**	whorl	whorl	1	whorl	1	whorl	1	equi (hr)	0.5
**Escalloniales**	whorl	whorl	1	whorl	1	whorl	0.97	equi (hr)	0.5
**Fabales**	whorl	whorl	1	whorl	1	whorl	0.99	equi (hr)	0.5
**Fagales**	whorl	whorl	1	whorl	1	whorl	0.99	equi (hr)	0.5
**Garryales**	whorl	whorl	1	whorl	1	whorl	0.99	whorl (hr)	0.51
**Gentianales**	whorl	whorl	1	whorl	1	whorl	0.99	equi (hr)	0.5
**Geraniales**	whorl	whorl	1	whorl	1	whorl	0.98	equi (hr)	0.5
**Huerteales**	whorl	whorl	1	whorl	1	whorl	0.96	equi (hr)	0.5
**Icacinales**	whorl	whorl	1	whorl	1	whorl	0.94	equi (hr)	0.5
**Lamiales**	whorl	whorl	1	whorl	1	whorl	0.99	equi (hr)	0.5
**Laurales**	whorl	whorl	1	whorl	1	whorl	0.97	equi (hr)	0.5
**Liliales**	whorl	whorl	1	whorl	1	whorl	0.99	equi (hr)	0.5
**Magnoliales**	whorl	whorl	1	whorl	1	whorl	0.89	equi (hr)	0.5
**Malpighiales**	whorl	whorl	1	whorl	1	whorl	1	equi (hr)	0.5
**Malvales**	whorl	whorl	1	whorl	1	whorl	0.99	equi (hr)	0.5
**Myrtales**	whorl	whorl	1	whorl	1	whorl	1	equi (hr)	0.5
**Nymphaeales**	whorl, spir	whorl	1	whorl	0.99	whorl	0.89	equi (hr)	0.5
**Oxalidales**	whorl	whorl	1	whorl	1	whorl	0.99	equi (hr)	0.5
**Pandanales**	whorl	whorl	1	whorl	1	whorl	0.98	equi (hr)	0.5
**Paracryphiales**	whorl	whorl	1	whorl	1	whorl	0.94	equi (hr)	0.5
**Petrosaviales**	whorl	whorl	1	whorl	1	whorl	0.87	equi (hr)	0.5
**Piperales**	whorl	whorl	1	whorl	1	whorl	0.97	equi (hr)	0.5

**Poales**	whorl	whorl	1	whorl	1	whorl	1	equi (hr)	0.5
**Proteales**	whorl	whorl	1	whorl	1	whorl	0.98	equi (hr)	0.5
**Ranunculales**	whorl	whorl	1	whorl	1	whorl	1	equi (hr)	0.5
**Rosales**	whorl	whorl	1	whorl	1	whorl	0.99	equi (hr)	0.5
**Santalales**	whorl	whorl	1	whorl	1	whorl	0.99	equi (hr)	0.5
**Sapindales**	whorl	whorl	1	whorl	1	whorl	0.99	equi (hr)	0.5
**Saxifragales**	whorl	whorl	1	whorl	1	whorl	1	equi (hr)	0.5
**Solanales**	whorl	whorl	1	whorl	1	whorl	0.99	equi (hr)	0.5
**Vitales**	whorl	whorl	1	whorl	1	whorl	0.87	equi (hr)	0.5
**Zingiberales**	whorl	whorl	1	whorl	1	whorl	1	equi (hr)	0.5
**Zygophyllales**	whorl	whorl	1	whorl	1	whorl	0.9	equi (hr)	0.5

**ORIGINAL DATA (Five-char)**	**Parsi**	**State for q=0.0001**	**State proba**	**State for q=0.001**	**State proba**	**State for q=0.01**	**State proba**	**State for q=0.1**	**State proba**
**Alismatales**	whorl	whorl	1	whorl	1	whorl	0.99	equi (hr)	0.5
**Apiales**	whorl	whorl	1	whorl	1	whorl	1	equi (hr)	0.5
**Aquifoliales**	whorl	whorl	1	whorl	1	whorl	0.98	equi (hr)	0.5
**Arecales**	whorl	whorl	1	whorl	1	whorl	0.94	equi (hr)	0.5
**Asparagales**	whorl	whorl	1	whorl	1	whorl	1	equi (hr)	0.5
**Asterales**	whorl	whorl	1	whorl	1	whorl	1	equi (hr)	0.5
**Austrobaileyales**	spir	**spir**	**0.99**	**spir**	**0.84**	**whorl**	**0.78**	equi (hr)	0.5
**Berberidopsidales**	whorl	whorl	0.99	whorl	0.92	whorl	0.58	equi (hr)	0.5
**Boraginales**	whorl	whorl	1	whorl	1	whorl	0.99	equi (hr)	0.5
**Brassicales**	whorl	whorl	1	whorl	1	whorl	1	equi (hr)	0.5
**Bruniales**	whorl	whorl	1	whorl	1	whorl	0.97	equi (hr)	0.5
**Buxales**	whorl	whorl	1	whorl	1	whorl	0.78	equi (hr)	0.5
**Canellales**	whorl	whorl	1	whorl	1	whorl	0.97	equi (hr)	0.5
**Caryophyllales**	whorl	whorl	1	whorl	1	whorl	1	equi (hr)	0.5
**Celastrales**	whorl	whorl	1	whorl	1	whorl	0.95	equi (hr)	0.5
**Commelinales**	whorl	whorl	1	whorl	1	whorl	1	equi (hr)	0.5
**Cornales**	whorl	whorl	1	whorl	1	whorl	0.99	equi (hr)	0.5
**Crossosomatales**	whorl	whorl	1	whorl	1	whorl	0.95	equi (hr)	0.5
**Cucurbitales**	whorl	whorl	1	whorl	1	whorl	0.99	equi (hr)	0.5
**Dioscoreales**	whorl	whorl	1	whorl	1	whorl	0.96	equi (hr)	0.5
**Dipsacales**	whorl	whorl	1	whorl	1	whorl	0.99	equi (hr)	0.5

**Ericales**	whorl	whorl	1	whorl	1	whorl	1	equi (hr)	0.5
**Escalloniales**	whorl	whorl	1	whorl	1	whorl	0.97	equi (hr)	0.5
**Fabales**	whorl	whorl	1	whorl	1	whorl	0.99	equi (hr)	0.5
**Fagales**	whorl	whorl	1	whorl	1	whorl	0.99	equi (hr)	0.5
**Garryales**	whorl	whorl	1	whorl	1	whorl	0.99	whorl (hr)	0.51
**Gentianales**	whorl	whorl	1	whorl	1	whorl	0.99	equi (hr)	0.5
**Geraniales**	whorl	whorl	1	whorl	1	whorl	0.99	equi (hr)	0.5
**Huerteales**	whorl	whorl	1	whorl	1	whorl	0.96	equi (hr)	0.5
**Icacinales**	whorl	whorl	1	whorl	1	whorl	0.94	equi (hr)	0.5
**Lamiales**	whorl	whorl	1	whorl	1	whorl	0.99	equi (hr)	0.5
**Laurales**	whorl	whorl	1	whorl	1	whorl	0.97	equi (hr)	0.5
**Liliales**	whorl	whorl	1	whorl	1	whorl	0.99	equi (hr)	0.5
**Magnoliales**	whorl	whorl	1	whorl	1	whorl	0.89	equi (hr)	0.5
**Malpighiales**	whorl	whorl	1	whorl	1	whorl	1	equi (hr)	0.5
**Malvales**	whorl	whorl	1	whorl	1	whorl	0.99	equi (hr)	0.5
**Myrtales**	whorl	whorl	1	whorl	1	whorl	1	equi (hr)	0.5
**Nymphaeales**	whorl, spir	whorl	1	whorl	0.99	whorl	0.89	equi (hr)	0.5
**Oxalidales**	whorl	whorl	1	whorl	1	whorl	0.99	equi (hr)	0.5
**Pandanales**	whorl	whorl	1	whorl	1	whorl	0.98	equi (hr)	0.5
**Paracryphiales**	whorl	whorl	1	whorl	1	whorl	0.94	equi (hr)	0.5
**Petrosaviales**	whorl	whorl	1	whorl	1	whorl	0.87	equi (hr)	0.5
**Piperales**	whorl	whorl	1	whorl	1	whorl	0.97	equi (hr)	0.5
**Poales**	whorl	whorl	1	whorl	1	whorl	1	equi (hr)	0.5
**Proteales**	whorl	whorl	1	whorl	1	whorl	0.98	equi (hr)	0.5
**Ranunculales**	whorl	whorl	1	whorl	1	whorl	1	equi (hr)	0.5
**Rosales**	whorl	whorl	1	whorl	1	whorl	0.99	equi (hr)	0.5
**Santalales**	whorl	whorl	1	whorl	1	whorl	0.99	equi (hr)	0.5
**Sapindales**	whorl	whorl	1	whorl	1	whorl	0.99	equi (hr)	0.5
**Saxifragales**	whorl	whorl	1	whorl	1	whorl	1	equi (hr)	0.5
**Solanales**	whorl	whorl	1	whorl	1	whorl	0.99	equi (hr)	0.5
**Vitales**	whorl	whorl	1	whorl	1	whorl	0.87	equi (hr)	0.5
**Zingiberales**	whorl	whorl	1	whorl	1	whorl	1	equi (hr)	0.5
**Zygophyllales**	whorl	whorl	1	whorl	1	whorl	0.9	equi (hr)	0.5

RESCORED DATA (PrunedMB)	Parsi	State for q=0.0001	State proba	State for q=0.001	State proba	State for q=0.01	State proba	State for q=0.1	State proba
**Alismatales**	whorl	whorl	1	whorl	1	whorl	0.99	equi (hr)	0.5
**Apiales**	whorl	whorl	1	whorl	1	whorl	1	equi (hr)	0.5
**Aquifoliales**	whorl	whorl	1	whorl	1	whorl	0.98	equi (hr)	0.5
**Arecales**	whorl	whorl	1	whorl	1	whorl	0.99	equi (hr)	0.5
**Asparagales**	whorl	whorl	1	whorl	1	whorl	1	equi (hr)	0.5
**Asterales**	whorl	whorl	1	whorl	1	whorl	1	equi (hr)	0.5
**Austrobaileyales**	spir	**spir**	**0.99**	**spir**	**0.84**	**whorl**	**0.78**	equi (hr)	0.5
**Berberidopsidales**	whorl	whorl	0.99	whorl	0.92	whorl	0.58	equi (hr)	0.5
**Boraginales**	whorl	whorl	1	whorl	1	whorl	0.97	equi (hr)	0.5
**Brassicales**	whorl	whorl	1	whorl	1	whorl	1	equi (hr)	0.5
**Bruniales**	whorl	whorl	1	whorl	1	whorl	0.97	equi (hr)	0.5
**Buxales**	whorl	whorl	1	whorl	0.96	whorl	0.7	equi (hr)	0.5
**Canellales**	whorl	whorl	1	whorl	1	whorl	0.96	equi (hr)	0.5
**Caryophyllales**	whorl	whorl	1	whorl	1	whorl	1	equi (hr)	0.5
**Celastrales**	whorl	whorl	1	whorl	1	whorl	0.92	equi (hr)	0.5
**Commelinales**	whorl	whorl	1	whorl	1	whorl	0.99	equi (hr)	0.5
**Cornales**	whorl	whorl	1	whorl	1	whorl	0.99	equi (hr)	0.5
**Crossosomatales**	whorl	whorl	1	whorl	1	whorl	0.94	equi (hr)	0.5
**Cucurbitales**	whorl	whorl	1	whorl	1	whorl	0.99	equi (hr)	0.5
**Dioscoreales**	whorl	whorl	1	whorl	1	whorl	0.96	equi (hr)	0.5
**Dipsacales**	whorl	whorl	1	whorl	1	whorl	0.99	equi (hr)	0.5
**Ericales**	whorl	whorl	1	whorl	1	whorl	1	equi (hr)	0.5
**Escalloniales**	whorl	whorl	1	whorl	1	whorl	0.99	equi (hr)	0.5
**Fabales**	whorl	whorl	1	whorl	1	whorl	0.99	equi (hr)	0.5
**Fagales**	whorl	whorl	1	whorl	1	whorl	0.99	equi (hr)	0.5
**Garryales**	whorl	whorl	1	whorl	1	whorl	0.99	whorl (hr)	0.51
**Gentianales**	whorl	whorl	1	whorl	1	whorl	1	equi (hr)	0.5
**Geraniales**	whorl	whorl	1	whorl	1	whorl	0.97	equi (hr)	0.5
**Huerteales**	whorl	whorl	1	whorl	1	whorl	0.97	equi (hr)	0.5
**Icacinales**	whorl	whorl	1	whorl	1	whorl	0.98	equi (hr)	0.5
**Lamiales**	whorl	whorl	1	whorl	1	whorl	0.99	equi (hr)	0.5
**Laurales**	whorl	whorl	1	whorl	1	whorl	0.94	equi (hr)	0.5
**Liliales**	whorl	whorl	1	whorl	1	whorl	0.98	equi (hr)	0.5

**Magnoliales**	whorl	whorl	1	whorl	1	whorl	0.71	equi (hr)	0.5
**Malpighiales**	whorl	whorl	1	whorl	1	whorl	1	equi (hr)	0.5
**Malvales**	whorl	whorl	1	whorl	1	whorl	0.99	equi (hr)	0.5
**Myrtales**	whorl	whorl	1	whorl	1	whorl	0.99	equi (hr)	0.5
**Nymphaeales**	whorl, spir	whorl	1	whorl	0.99	whorl	0.89	equi (hr)	0.5
**Oxalidales**	whorl	whorl	1	whorl	1	whorl	0.99	equi (hr)	0.5
**Pandanales**	whorl	whorl	1	whorl	1	whorl	0.95	equi (hr)	0.5
**Paracryphiales**	whorl	whorl	1	whorl	1	whorl	0.94	equi (hr)	0.5
**Petrosaviales**	whorl	whorl	1	whorl	1	whorl	0.87	equi (hr)	0.5
**Piperales**	whorl	whorl	1	whorl	1	whorl	0.96	equi (hr)	0.5
**Poales**	whorl	whorl	1	whorl	1	whorl	1	equi (hr)	0.5
**Proteales**	whorl	whorl	1	whorl	1	whorl	0.99	equi (hr)	0.5
**Ranunculales**	whorl	whorl	1	whorl	1	whorl	1	equi (hr)	0.5
**Rosales**	whorl	whorl	1	whorl	1	whorl	0.99	equi (hr)	0.5
**Santalales**	whorl	whorl	1	whorl	1	whorl	0.99	equi (hr)	0.5
**Sapindales**	whorl	whorl	1	whorl	1	whorl	1	equi (hr)	0.5
**Saxifragales**	whorl	whorl	1	whorl	1	whorl	1	equi (hr)	0.5
**Solanales**	whorl	whorl	1	whorl	1	whorl	0.99	equi (hr)	0.5
**Vitales**	whorl	whorl	1	whorl	1	whorl	0.87	equi (hr)	0.5
**Zingiberales**	whorl	whorl	1	whorl	1	whorl	0.99	equi (hr)	0.5
**Zygophyllales**	whorl	whorl	1	whorl	1	whorl	0.98	equi (hr)	0.5

Merism

q= transition rate (changes per Ma); Parsi= parsimony; State proba= state probability; equi= equivocal; (hr)= high transition rate equilibrium; whorl= whorled; spir= spiral; penta= pentamerous; no-p= non-pentamerous

RESCORED DATA (Five-char)	Parsi	State for q=0.0001	State proba	State for q=0.001	State proba	State for q=0.01	State proba	State for q=0.1	State proba
**Alismatales**	no-p	no-p	1	no-p	1	no-p	0.99	equi (hr)	0.5
**Apiales**	penta	penta	1	penta	1	penta	1	equi (hr)	0.5
**Aquifoliales**	penta	penta	1	penta	1	penta	0.96	equi (hr)	0.5
**Arecales**	no-p	no-p	1	no-p	1	no-p	0.94	equi (hr)	0.5
**Asparagales**	no-p	no-p	1	no-p	1	no-p	1	equi (hr)	0.5
**Asterales**	penta	penta	1	penta	1	penta	1	equi (hr)	0.5
**Austrobaileyales**	?	?	?	?	?	?	?	?	?
**Berberidopsidales**	penta	?	?	?	?	?	?	?	?

**Boraginales**	penta	penta	1	penta	1	penta	0.97	equi (hr)	0.5
**Brassicales**	penta	penta	1	penta	1	penta	0.94	equi (hr)	0.5
**Bruniales**	penta	penta	1	penta	1	penta	0.97	equi (hr)	0.5
**Buxales**	no-p	no-p	1	no-p	1	no-p	0.72	equi (hr)	0.5
**Canellales**	no-p	no-p	1	no-p	1	no-p	0.95	equi (hr)	0.5
**Caryophyllales**	penta	penta	1	penta	1	penta	1	equi (hr)	0.5
**Celastrales**	penta	penta	1	penta	1	penta	0.88	equi (hr)	0.5
**Commelinales**	no-p	no-p	1	no-p	1	no-p	1	equi (hr)	0.5
**Cornales**	penta	penta	1	penta	1	penta	0.94	equi (hr)	0.5
**Crossosomatales**	penta	penta	1	penta	1	penta	0.92	equi (hr)	0.5
**Cucurbitales**	penta	penta	1	penta	1	penta	0.88	equi (hr)	0.5
**Dioscoreales**	no-p	no-p	1	no-p	1	no-p	0.96	equi (hr)	0.5
**Dipsacales**	penta	penta	1	penta	1	penta	0.97	equi (hr)	0.5
**Ericales**	penta	penta	1	penta	1	penta	1	equi (hr)	0.5
**Escalloniales**	penta	penta	1	penta	0.99	penta	0.93	equi (hr)	0.5
**Fabales**	penta	penta	1	penta	1	penta	0.98	equi (hr)	0.5
**Fagales**	no-p	**no-p**	**0.99**	**no-p**	**0.92**	**penta**	**0.65**	equi (hr)	0.5
**Garryales**	no-p, penta	no-p	1	no-p	0.98	no-p	0.83	no-p (hr)	0.51
**Gentianales**	penta	penta	1	penta	1	penta	0.97	equi (hr)	0.5
**Geraniales**	penta	penta	1	penta	1	penta	0.97	equi (hr)	0.5
**Huerteales**	penta	penta	1	penta	1	penta	0.93	equi (hr)	0.5
**Icacinales**	penta	penta	1	penta	1	penta	0.83	equi (hr)	0.5
**Lamiales**	penta	penta	1	penta	1	penta	0.98	equi (hr)	0.5
**Laurales**	no-p	no-p	1	no-p	1	no-p	0.99	equi (hr)	0.5
**Liliales**	no-p	no-p	1	no-p	1	no-p	0.99	equi (hr)	0.5
**Magnoliales**	no-p	no-p	1	no-p	1	no-p	0.94	equi (hr)	0.5
**Malpighiales**	penta	penta	1	penta	1	penta	1	equi (hr)	0.5
**Malvales**	penta	penta	1	penta	1	penta	0.95	equi (hr)	0.5
**Myrtales**	penta	penta	1	penta	1	penta	0.91	equi (hr)	0.5
**Nymphaeales**	no-p	no-p	1	no-p	1	no-p	0.9	equi (hr)	0.5
**Oxalidales**	penta	penta	1	penta	1	penta	0.98	equi (hr)	0.5
**Pandanales**	no-p	no-p	1	no-p	1	no-p	0.96	equi (hr)	0.5
**Paracryphiales**	penta	penta	1	penta	0.97	penta	0.77	equi (hr)	0.5
**Petrosaviales**	no-p	no-p	1	no-p	1	no-p	0.87	equi (hr)	0.5

**Piperales**	no-p	no-p	1	no-p	1	no-p	0.96	equi (hr)	0.5
**Poales**	no-p	no-p	1	no-p	1	no-p	1	equi (hr)	0.5
**Proteales**	no-p, penta	no-p	0.97	no-p	0.97	no-p	0.8	equi (hr)	0.5
**Ranunculales**	no-p	no-p	1	no-p	1	no-p	0.95	equi (hr)	0.5
**Rosales**	penta	penta	1	penta	1	penta	0.92	equi (hr)	0.5
**Santalales**	penta	penta	1	penta	0.99	penta	0.83	equi (hr)	0.5
**Sapindales**	penta	penta	1	penta	1	penta	0.97	equi (hr)	0.5
**Saxifragales**	penta	penta	1	penta	1	penta	0.95	equi (hr)	0.5
**Solanales**	penta	penta	1	penta	1	penta	0.98	equi (hr)	0.5
**Vitales**	penta	penta	0.99	penta	0.94	penta	0.64	equi (hr)	0.5
**Zingiberales**	no-p	no-p	1	no-p	1	no-p	1	equi (hr)	0.5
**Zygophyllales**	penta	penta	1	penta	1	penta	0.9	equi (hr)	0.5

**ORIGINAL DATA (Five-char)**	**Parsi**	**State for q=0.0001**	**State proba**	**State for q=0.001**	**State proba**	**State for q=0.01**	**State proba**	**State for q=0.1**	**State proba**
**Alismatales**	no-p	no-p	1	no-p	1	no-p	0.99	equi (hr)	0.5
**Apiales**	penta	penta	1	penta	1	penta	0.99	equi (hr)	0.5
**Aquifoliales**	penta	penta	1	penta	1	penta	0.91	equi (hr)	0.5
**Arecales**	no-p	no-p	1	no-p	1	no-p	0.94	equi (hr)	0.5
**Asparagales**	no-p	no-p	1	no-p	1	no-p	1	equi (hr)	0.5
**Asterales**	penta	penta	1	penta	1	penta	0.99	equi (hr)	0.5
**Austrobaileyales**	?	?	?	?	?	?	?	?	?
**Berberidopsidales**	no-p, penta	?	?	?	?	?	?	?	?
**Boraginales**	penta	penta	1	penta	1	penta	0.97	equi (hr)	0.5
**Brassicales**	penta	penta	1	penta	1	penta	0.85	equi (hr)	0.5
**Bruniales**	penta	penta	1	penta	1	penta	0.97	equi (hr)	0.5
**Buxales**	no-p	no-p	1	no-p	1	no-p	0.74	equi (hr)	0.5
**Canellales**	no-p	no-p	1	no-p	1	no-p	0.97	equi (hr)	0.5
**Caryophyllales**	penta	penta	1	penta	1	penta	0.99	equi (hr)	0.5
**Celastrales**	penta	penta	1	penta	1	penta	0.86	equi (hr)	0.5
**Commelinales**	no-p	no-p	1	no-p	1	no-p	1	equi (hr)	0.5
**Cornales**	penta	penta	1	penta	0.98	penta	0.87	equi (hr)	0.5
**Crossosomatales**	penta	penta	1	penta	1	penta	0.91	equi (hr)	0.5
**Cucurbitales**	no-p	no-p	0.98	no-p	0.81	penta	0.54	equi (hr)	0.5

**Dioscoreales**	no-p	no-p	1	no-p	1	no-p	0.96	equi (hr)	0.5
**Dipsacales**	penta	penta	1	penta	1	penta	0.97	equi (hr)	0.5
**Ericales**	penta	penta	1	penta	1	penta	0.99	equi (hr)	0.5
**Escalloniales**	penta	penta	1	penta	0.99	penta	0.93	equi (hr)	0.5
**Fabales**	penta	penta	1	penta	1	penta	0.96	equi (hr)	0.5
**Fagales**	no-p	**no-p**	**1**	**no-p**	**0.99**	**penta**	**0.55**	equi (hr)	0.5
**Garryales**	no-p, penta	no-p	1	no-p	0.98	no-p	0.83	no-p (hr)	0.51
**Gentianales**	penta	penta	1	penta	1	penta	0.94	equi (hr)	0.5
**Geraniales**	penta	penta	1	penta	1	penta	0.94	equi (hr)	0.5
**Huerteales**	penta	penta	1	penta	1	penta	0.88	equi (hr)	0.5
**Icacinales**	penta	penta	1	penta	1	penta	0.83	equi (hr)	0.5
**Lamiales**	penta	penta	1	penta	1	penta	0.95	equi (hr)	0.5
**Laurales**	no-p	no-p	1	no-p	1	no-p	0.99	equi (hr)	0.5
**Liliales**	no-p	no-p	1	no-p	1	no-p	0.99	equi (hr)	0.5
**Magnoliales**	no-p	no-p	1	no-p	1	no-p	0.95	equi (hr)	0.5
**Malpighiales**	penta	penta	1	penta	1	penta	1	equi (hr)	0.5
**Malvales**	penta	penta	1	penta	1	penta	0.91	equi (hr)	0.5
**Myrtales**	penta	penta	1	penta	1	penta	0.87	equi (hr)	0.5
**Nymphaeales**	no-p	no-p	1	no-p	1	no-p	0.9	equi (hr)	0.5
**Oxalidales**	penta	penta	1	penta	1	penta	0.94	equi (hr)	0.5
**Pandanales**	no-p	no-p	1	no-p	1	no-p	0.96	equi (hr)	0.5
**Paracryphiales**	penta	penta	1	penta	0.97	penta	0.71	equi (hr)	0.5
**Petrosaviales**	no-p	no-p	1	no-p	1	no-p	0.77	equi (hr)	0.5
**Piperales**	no-p	no-p	1	no-p	1	no-p	0.87	equi (hr)	0.5
**Poales**	no-p	no-p	1	no-p	1	no-p	1	equi (hr)	0.5
**Proteales**	no-p	no-p	1	no-p	1	no-p	0.85	equi (hr)	0.5
**Ranunculales**	no-p	no-p	1	no-p	1	no-p	0.96	equi (hr)	0.5
**Rosales**	no-p	**no-p**	**0.98**	**no-p**	**0.84**	**penta**	**0.74**	equi (hr)	0.5
**Santalales**	no-p, penta	no-p	0.83	no-p	0.81	equi	0.5	equi (hr)	0.5
**Sapindales**	penta	penta	1	penta	1	penta	0.86	equi (hr)	0.5
**Saxifragales**	penta	penta	1	penta	0.99	penta	0.83	equi (hr)	0.5
**Solanales**	penta	penta	1	penta	1	penta	0.98	equi (hr)	0.5
**Vitales**	penta	penta	0.99	penta	0.94	penta	0.62	equi (hr)	0.5
**Zingiberales**	no-p	no-p	1	no-p	1	no-p	1	equi (hr)	0.5

**Zygophyllales**	penta	penta	1	penta	1	penta	0.82	equi (hr)	0.5

**RESCORED DATA (PrunedMB)**	**Parsi**	**State for q=0.0001**	**State proba**	**State for q=0.001**	**State proba**	**State for q=0.01**	**State proba**	**State for q=0.1**	**State proba**
**Alismatales**	no-p	no-p	1	no-p	1	no-p	0.99	equi (hr)	0.5
**Apiales**	penta	penta	1	penta	1	penta	1	equi (hr)	0.5
**Aquifoliales**	penta	penta	1	penta	1	penta	0.96	equi (hr)	0.5
**Arecales**	no-p	no-p	1	no-p	1	no-p	0.99	equi (hr)	0.5
**Asparagales**	no-p	no-p	1	no-p	1	no-p	1	equi (hr)	0.5
**Asterales**	penta	penta	1	penta	1	penta	1	equi (hr)	0.5
**Austrobaileyales**	?	?	?	?	?	?	?	?	?
**Berberidopsidales**	penta	?	?	?	?	?	?	?	?
**Boraginales**	penta	penta	1	penta	1	penta	0.92	equi (hr)	0.5
**Brassicales**	penta	penta	1	penta	1	penta	0.92	equi (hr)	0.5
**Bruniales**	penta	penta	1	penta	1	penta	0.97	equi (hr)	0.5
**Buxales**	no-p	no-p	1	no-p	1	no-p	0.89	equi (hr)	0.5
**Canellales**	no-p	no-p	1	no-p	1	no-p	0.96	equi (hr)	0.5
**Caryophyllales**	penta	penta	1	penta	1	penta	1	equi (hr)	0.5
**Celastrales**	penta	penta	1	penta	1	penta	0.88	equi (hr)	0.5
**Commelinales**	no-p	no-p	1	no-p	1	no-p	0.99	equi (hr)	0.5
**Cornales**	penta	penta	1	penta	1	penta	0.97	equi (hr)	0.5
**Crossosomatales**	penta	penta	1	penta	1	penta	0.92	equi (hr)	0.5
**Cucurbitales**	penta	penta	1	penta	1	penta	0.88	equi (hr)	0.5
**Dioscoreales**	no-p	no-p	1	no-p	1	no-p	0.96	equi (hr)	0.5
**Dipsacales**	penta	penta	1	penta	1	penta	0.97	equi (hr)	0.5
**Ericales**	penta	penta	1	penta	1	penta	1	equi (hr)	0.5
**Escalloniales**	penta	penta	1	penta	0.99	penta	0.99	equi (hr)	0.5
**Fabales**	penta	penta	1	penta	1	penta	0.99	equi (hr)	0.5
**Fagales**	no-p	**no-p**	**0.99**	**no-p**	**0.92**	**penta**	**0.7**	equi (hr)	0.5
**Garryales**	no-p, penta	no-p	1	no-p	0.99	no-p	0.92	no-p (hr)	0.51
**Gentianales**	penta	penta	1	penta	1	penta	0.99	equi (hr)	0.5
**Geraniales**	penta	penta	1	penta	1	penta	0.97	equi (hr)	0.5
**Huerteales**	penta	penta	1	penta	1	penta	0.94	equi (hr)	0.5
**Icacinales**	no-p, penta	no-p	1	no-p	0.59	no-p	0.55	equi (hr)	0.5

**Lamiales**	penta	penta	1	penta	1	penta	0.96	equi (hr)	0.5
**Laurales**	no-p	no-p	1	no-p	1	no-p	0.98	equi (hr)	0.5
**Liliales**	no-p	no-p	1	no-p	1	no-p	0.98	equi (hr)	0.5
**Magnoliales**	no-p	no-p	1	no-p	1	no-p	0.96	equi (hr)	0.5
**Malpighiales**	penta	penta	1	penta	1	penta	1	equi (hr)	0.5
**Malvales**	penta	penta	1	penta	1	penta	0.95	equi (hr)	0.5
**Myrtales**	penta	penta	1	penta	1	penta	0.88	equi (hr)	0.5
**Nymphaeales**	no-p	no-p	1	no-p	1	no-p	0.89	equi (hr)	0.5
**Oxalidales**	penta	penta	1	penta	1	penta	0.97	equi (hr)	0.5
**Pandanales**	no-p	no-p	1	no-p	1	no-p	0.93	equi (hr)	0.5
**Paracryphiales**	penta	penta	1	penta	0.97	penta	0.77	equi (hr)	0.5
**Petrosaviales**	no-p	no-p	1	no-p	1	no-p	0.87	equi (hr)	0.5
**Piperales**	no-p	no-p	1	no-p	1	no-p	0.96	equi (hr)	0.5
**Poales**	no-p	no-p	1	no-p	1	no-p	1	equi (hr)	0.5
**Proteales**	no-p. penta	no-p	0.84	no-p	0.85	no-p	0.74	equi (hr)	0.5
**Ranunculales**	no-p	no-p	1	no-p	1	no-p	0.94	equi (hr)	0.5
**Rosales**	no-p, penta	penta	1	penta	1	penta	0.91	equi (hr)	0.5
**Santalales**	penta	penta	1	penta	1	penta	0.85	equi (hr)	0.5
**Sapindales**	penta	penta	1	penta	1	penta	0.97	equi (hr)	0.5
**Saxifragales**	penta	penta	1	penta	1	penta	0.98	equi (hr)	0.5
**Solanales**	penta	penta	1	penta	1	penta	0.98	equi (hr)	0.5
**Vitales**	penta	penta	0.99	penta	0.94	penta	0.64	equi (hr)	0.5
**Zingiberales**	no-p	no-p	1	no-p	1	no-p	0.99	equi (hr)	0.5
**Zygophyllales**	penta	penta	1	penta	1	penta	0.94	equi (hr)	0.5

Differentiation

q= transition rate (changes per Ma); Parsi= parsimony; State proba= state probability; equi= equivocal; (hr)= high transition rate equilibrium; undif= undifferentiated; diff= differentiated; **state change pattern**; *non-recognized pattern*

**RESCORED DATA (Five-char)**	**Parsi**	**State for q=0.0001**	**State proba**	**State for q=0.001**	**State proba**	**State for q=0.01**	**State proba**	**State for q=0.1**	**State proba**
**Alismatales**	undif	**undif**	**1**	**undif**	**0.75**	**diff**	**0.83**	equi (hr)	0.5
**Apiales**	diff	diff	1	diff	1	diff	1	equi (hr)	0.5
**Aquifoliales**	diff	diff	1	diff	1	diff	0.98	equi (hr)	0.5
**Arecales**	undif, diff	*diff*	*0.74*	*diff*	*0.81*	*diff*	*0.73*	equi (hr)	0.5

**Asparagales**	undif, diff	**undif**	**0.91**	**undif**	**0.63**	**diff**	**0.68**	equi (hr)	0.5
**Asterales**	diff	diff	1	diff	1	diff	1	equi (hr)	0.5
**Austrobaileyales**	undif	**undif**	**0.96**	**undif**	**0.59**	**diff**	**0.79**	equi (hr)	0.5
**Berberidopsidales**	diff	diff	0.99	diff	0.92	diff	0.58	equi (hr)	0.5
**Boraginales**	diff	diff	1	diff	1	diff	0.99	equi (hr)	0.5
**Brassicales**	diff	diff	1	diff	1	diff	1	equi (hr)	0.5
**Bruniales**	diff	diff	1	diff	1	diff	0.97	equi (hr)	0.5
**Buxales**	diff	diff	1	diff	1	diff	0.77	equi (hr)	0.5
**Canellales**	diff	*diff*	*0.85*	*diff*	*0.65*	*diff*	*0.89*	equi (hr)	0.5
**Caryophyllales**	diff	diff	1	diff	1	diff	0.9	equi (hr)	0.5
**Celastrales**	diff	diff	1	diff	1	diff	0.95	equi (hr)	0.5
**Commelinales**	diff	diff	1	diff	1	diff	0.96	equi (hr)	0.5
**Cornales**	diff	diff	1	diff	1	diff	0.98	equi (hr)	0.5
**Crossosomatales**	diff	diff	1	diff	1	diff	0.96	equi (hr)	0.5
**Cucurbitales**	diff	diff	1	diff	0.99	diff	0.87	equi (hr)	0.5
**Dioscoreales**	undif	**undif**	**1**	**undif**	**0.91**	**diff**	**0.57**	equi (hr)	0.5
**Dipsacales**	diff	diff	1	diff	1	diff	0.99	equi (hr)	0.5
**Ericales**	diff	diff	1	diff	1	diff	1	equi (hr)	0.5
**Escalloniales**	diff	diff	1	diff	1	diff	0.97	equi (hr)	0.5
**Fabales**	diff	diff	1	diff	1	diff	0.97	equi (hr)	0.5
**Fagales**	undif	**undif**	**0.99**	**undif**	**0.92**	**diff**	**0.6**	equi (hr)	0.5
**Garryales**	diff	diff	1	diff	0.98	diff	0.82	equi (hr)	0.5
**Gentianales**	diff	diff	1	diff	1	diff	0.99	equi (hr)	0.5
**Geraniales**	diff	diff	1	diff	1	diff	0.98	equi (hr)	0.5
**Huerteales**	diff	diff	0.99	diff	0.88	diff	0.61	equi (hr)	0.5
**Icacinales**	diff	diff	1	diff	1	diff	0.92	equi (hr)	0.5
**Lamiales**	diff	diff	1	diff	1	diff	0.99	equi (hr)	0.5
**Laurales**	undif	**undif**	**0.94**	**undif**	**0.53**	**diff**	**0.8**	equi (hr)	0.5
**Liliales**	undif, diff	**undif**	**0.93**	**undif**	**0.61**	**diff**	**0.8**	equi (hr)	0.5
**Magnoliales**	undif	**undif**	**0.93**	**diff**	**0.53**	**diff**	**0.87**	equi (hr)	0.5
**Malpighiales**	diff	diff	1	diff	1	diff	1	equi (hr)	0.5
**Malvales**	diff	diff	1	diff	1	diff	0.96	equi (hr)	0.5
**Myrtales**	diff	diff	1	diff	1	diff	0.99	equi (hr)	0.5
**Nymphaeales**	undif	**undif**	**0.96**	**undif**	**0.57**	**diff**	**0.79**	equi (hr)	0.5

**Oxalidales**	diff	diff	1	diff	1	diff	0.99	equi (hr)	0.5
**Pandanales**	undif	undif	1	undif	1	undif	0.85	equi (hr)	0.5
**Paracryphiales**	diff	diff	1	diff	0.97	diff	0.78	equi (hr)	0.5
**Petrosaviales**	diff	diff	0.97	diff	0.82	diff	0.78	equi (hr)	0.5
**Piperales**	undif	**undif**	**0.98**	**undif**	**0.77**	**diff**	**0.62**	equi (hr)	0.5
**Poales**	diff	diff	1	diff	0.99	diff	0.86	equi (hr)	0.5
**Proteales**	diff	diff	1	diff	1	diff	0.97	equi (hr)	0.5
**Ranunculales**	diff	diff	1	diff	1	diff	0.98	equi (hr)	0.5
**Rosales**	diff	diff	1	diff	0.97	diff	0.78	equi (hr)	0.5
**Santalales**	diff	diff	1	diff	1	diff	0.98	equi (hr)	0.5
**Sapindales**	diff	diff	1	diff	1	diff	0.99	equi (hr)	0.5
**Saxifragales**	diff	diff	1	diff	1	diff	0.99	equi (hr)	0.5
**Solanales**	diff	diff	1	diff	1	diff	0.99	equi (hr)	0.5
**Vitales**	diff	diff	1	diff	1	diff	0.87	equi (hr)	0.5
**Zingiberales**	diff	diff	1	diff	1	diff	0.94	equi (hr)	0.5
**Zygophyllales**	diff	diff	1	diff	1	diff	0.99	diff (hr)	0.54

**ORIGINAL DATA (Five-char)**	**Parsi**	**State for q=0.0001**	**State proba**	**State for q=0.001**	**State proba**	**State for q=0.01**	**State proba**	**State for q=0.1**	**State proba**
**Alismatales**	undif	**undif**	**1**	**undif**	**0.88**	**diff**	**0.8**	equi (hr)	0.5
**Apiales**	diff	diff	1	diff	1	diff	1	equi (hr)	0.5
**Aquifoliales**	diff	diff	1	diff	1	diff	0.98	equi (hr)	0.5
**Arecales**	undif, diff	*diff*	*0.73*	*diff*	*0.73*	*diff*	*0.71*	equi (hr)	0.5
**Asparagales**	undif, diff	**undif**	**0.92**	**undif**	**0.77**	**diff**	**0.65**	equi (hr)	0.5
**Asterales**	diff	diff	1	diff	1	diff	1	equi (hr)	0.5
**Austrobaileyales**	undif	**undif**	**0.98**	**undif**	**0.79**	**diff**	**0.77**	equi (hr)	0.5
**Berberidopsidales**	diff	diff	0.99	diff	0.92	diff	0.58	equi (hr)	0.5
**Boraginales**	diff	diff	1	diff	1	diff	0.99	equi (hr)	0.5
**Brassicales**	diff	diff	1	diff	1	diff	1	equi (hr)	0.5
**Bruniales**	diff	diff	1	diff	1	diff	0.97	equi (hr)	0.5
**Buxales**	diff	diff	1	diff	1	diff	0.77	equi (hr)	0.5
**Canellales**	diff	*diff*	*0.84*	*diff*	*0.51*	*diff*	*0.86*	equi (hr)	0.5
**Caryophyllales**	diff	diff	1	diff	1	diff	0.9	equi (hr)	0.5
**Celastrales**	diff	diff	1	diff	1	diff	0.95	equi (hr)	0.5

**Commelinales**	diff	diff	1	diff	1	diff	0.96	equi (hr)	0.5
**Cornales**	diff	diff	1	diff	1	diff	0.98	equi (hr)	0.5
**Crossosomatales**	diff	diff	1	diff	1	diff	0.96	equi (hr)	0.5
**Cucurbitales**	diff	diff	1	diff	0.99	diff	0.87	equi (hr)	0.5
**Dioscoreales**	undif	**undif**	**1**	**undif**	**0.95**	**diff**	**0.55**	equi (hr)	0.5
**Dipsacales**	diff	diff	1	diff	1	diff	0.99	equi (hr)	0.5
**Ericales**	diff	diff	1	diff	1	diff	1	equi (hr)	0.5
**Escalloniales**	diff	diff	1	diff	1	diff	0.97	equi (hr)	0.5
**Fabales**	diff	diff	1	diff	1	diff	0.97	equi (hr)	0.5
**Fagales**	undif	**undif**	**0.99**	**undif**	**0.92**	**diff**	**0.6**	equi (hr)	0.5
**Garryales**	diff	diff	1	diff	0.98	diff	0.82	equi (hr)	0.5
**Gentianales**	diff	diff	1	diff	1	diff	0.99	equi (hr)	0.5
**Geraniales**	diff	diff	1	diff	1	diff	0.98	equi (hr)	0.5
**Huerteales**	diff	diff	0.99	diff	0.88	diff	0.61	equi (hr)	0.5
**Icacinales**	diff	diff	1	diff	1	diff	0.92	equi (hr)	0.5
**Lamiales**	diff	diff	1	diff	1	diff	0.99	equi (hr)	0.5
**Laurales**	undif	**undif**	**0.98**	**undif**	**0.77**	**diff**	**0.72**	equi (hr)	0.5
**Liliales**	undif, diff	**undif**	**0.94**	**undif**	**0.75**	**diff**	**0.76**	equi (hr)	0.5
**Magnoliales**	undif	**undif**	**0.97**	**undif**	**0.68**	**diff**	**0.84**	equi (hr)	0.5
**Malpighiales**	diff	diff	1	diff	1	diff	1	equi (hr)	0.5
**Malvales**	diff	diff	1	diff	1	diff	0.96	equi (hr)	0.5
**Myrtales**	diff	diff	1	diff	1	diff	0.99	equi (hr)	0.5
**Nymphaeales**	undif	**undif**	**0.98**	**undif**	**0.77**	**diff**	**0.78**	equi (hr)	0.5
**Oxalidales**	diff	diff	1	diff	1	diff	0.99	equi (hr)	0.5
**Pandanales**	undif	undif	1	undif	1	undif	0.85	equi (hr)	0.5
**Paracryphiales**	diff	diff	1	diff	0.97	diff	0.78	equi (hr)	0.5
**Petrosaviales**	diff	diff	0.97	diff	0.78	diff	0.76	equi (hr)	0.5
**Piperales**	undif	**undif**	**0.99**	**undif**	**0.89**	**diff**	**0.61**	equi (hr)	0.5
**Poales**	diff	diff	1	diff	0.94	diff	0.82	equi (hr)	0.5
**Proteales**	diff	diff	1	diff	1	diff	0.96	equi (hr)	0.5
**Ranunculales**	diff	diff	1	diff	1	diff	0.97	equi (hr)	0.5
**Rosales**	diff	diff	0.99	diff	0.95	diff	0.75	equi (hr)	0.5
**Santalales**	diff	diff	1	diff	1	diff	0.98	equi (hr)	0.5
**Sapindales**	diff	diff	1	diff	1	diff	0.99	equi (hr)	0.5

**Saxifragales**	diff	diff	1	diff	1	diff	0.98	equi (hr)	0.5
**Solanales**	diff	diff	1	diff	1	diff	0.99	equi (hr)	0.5
**Vitales**	diff	diff	1	diff	1	diff	0.87	equi (hr)	0.5
**Zingiberales**	diff	diff	1	diff	1	diff	0.94	equi (hr)	0.5
**Zygophyllales**	diff	diff	1	diff	1	diff	0.99	diff (hr)	0.54

**RESCORED DATA (PrunedMB)**	**Parsi**	**State for q=0.0001**	**State proba**	**State for q=0.001**	**State proba**	**State for q=0.01**	**State proba**	**State for q=0.1**	**State proba**
**Alismatales**	undif	**undif**	**0.98**	**undif**	**0.74**	**diff**	**0.84**	equi (hr)	0.5
**Apiales**	diff	diff	1	diff	1	diff	1	equi (hr)	0.5
**Aquifoliales**	diff	diff	1	diff	1	diff	0.98	equi (hr)	0.5
**Arecales**	diff	diff	1	diff	0.98	diff	0.89	equi (hr)	0.5
**Asparagales**	undif, diff	**undif**	**0.89**	**undif**	**0.69**	**diff**	**0.72**	equi (hr)	0.5
**Asterales**	diff	diff	1	diff	1	diff	1	equi (hr)	0.5
**Austrobaileyales**	undif	**diff**	**0.53**	**diff**	**0.68**	**diff**	**0.79**	equi (hr)	0.5
**Berberidopsidales**	diff	diff	0.99	diff	0.92	diff	0.58	equi (hr)	0.5
**Boraginales**	diff	diff	1	diff	1	diff	0.97	equi (hr)	0.5
**Brassicales**	diff	diff	1	diff	1	diff	1	equi (hr)	0.5
**Bruniales**	diff	diff	1	diff	1	diff	0.97	equi (hr)	0.5
**Buxales**	diff	diff	1	diff	1	diff	0.75	equi (hr)	0.5
**Canellales**	diff	*diff*	*0.95*	*diff*	*0.87*	*diff*	*0.9*	equi (hr)	0.5
**Caryophyllales**	diff	diff	1	diff	1	diff	0.9	equi (hr)	0.5
**Celastrales**	diff	diff	1	diff	1	diff	0.92	equi (hr)	0.5
**Commelinales**	diff	diff	1	diff	1	diff	0.97	equi (hr)	0.5
**Cornales**	diff	diff	1	diff	1	diff	0.99	equi (hr)	0.5
**Crossosomatales**	diff	diff	1	diff	1	diff	0.96	equi (hr)	0.5
**Cucurbitales**	diff	diff	1	diff	1	diff	0.9	equi (hr)	0.5
**Dioscoreales**	undif	**undif**	**1**	**undif**	**0.98**	**diff**	**0.56**	equi (hr)	0.5
**Dipsacales**	diff	diff	1	diff	1	diff	0.99	equi (hr)	0.5
**Ericales**	diff	diff	1	diff	1	diff	1	equi (hr)	0.5
**Escalloniales**	diff	diff	1	diff	1	diff	0.99	equi (hr)	0.5
**Fabales**	diff	diff	1	diff	1	diff	0.98	equi (hr)	0.5
**Fagales**	undif	**undif**	**0.99**	**undif**	**0.92**	**diff**	**0.68**	equi (hr)	0.5
**Garryales**	diff	diff	1	diff	0.98	diff	0.72	equi (hr)	0.5
**Gentianales**	diff	diff	1	diff	1	diff	1	equi (hr)	0.5

**Geraniales**	diff	diff	1	diff	1	diff	0.97	equi (hr)	0.5
**Huerteales**	diff	diff	0.99	diff	0.88	diff	0.57	equi (hr)	0.5
**Icacinales**	diff	diff	1	diff	1	diff	0.84	equi (hr)	0.5
**Lamiales**	diff	diff	1	diff	1	diff	0.99	equi (hr)	0.5
**Laurales**	undif	**diff**	**0.71**	**diff**	**0.86**	**diff**	**0.84**	equi (hr)	0.5
**Liliales**	undif, diff	**undif**	**0.92**	**undif**	**0.72**	**diff**	**0.66**	equi (hr)	0.5
**Magnoliales**	undif	**diff**	**0.74**	**diff**	**0.9**	**diff**	**0.89**	equi (hr)	0.5
**Malpighiales**	diff	diff	1	diff	1	diff	1	equi (hr)	0.5
**Malvales**	diff	diff	1	diff	1	diff	0.97	equi (hr)	0.5
**Myrtales**	diff	diff	1	diff	1	diff	0.98	equi (hr)	0.5
**Nymphaeales**	undif	**diff**	**0.53**	**diff**	**0.72**	**diff**	**0.8**	equi (hr)	0.5
**Oxalidales**	diff	diff	1	diff	1	diff	0.99	equi (hr)	0.5
**Pandanales**	undif	undif	1	undif	1	undif	0.8	equi (hr)	0.5
**Paracryphiales**	diff	diff	1	diff	0.97	diff	0.78	equi (hr)	0.5
**Petrosaviales**	diff	diff	0.97	diff	0.82	diff	0.78	equi (hr)	0.5
**Piperales**	undif	**undif**	**1**	**undif**	**0.95**	**diff**	**0.61**	equi (hr)	0.5
**Poales**	diff	diff	1	diff	0.98	diff	0.86	equi (hr)	0.5
**Proteales**	diff	diff	1	diff	1	diff	0.96	equi (hr)	0.5
**Ranunculales**	diff	diff	1	diff	1	diff	0.98	equi (hr)	0.5
**Rosales**	diff	diff	1	diff	0.98	diff	0.83	equi (hr)	0.5
**Santalales**	diff	diff	1	diff	1	diff	0.95	equi (hr)	0.5
**Sapindales**	diff	diff	1	diff	1	diff	1	equi (hr)	0.5
**Saxifragales**	diff	diff	1	diff	1	diff	1	equi (hr)	0.5
**Solanales**	diff	diff	1	diff	1	diff	0.99	equi (hr)	0.5
**Vitales**	diff	diff	1	diff	1	diff	0.87	equi (hr)	0.5
**Zingiberales**	diff	diff	1	diff	1	diff	0.98	equi (hr)	0.5
**Zygophyllales**	diff	diff	1	diff	1	diff	1	equi (hr)	0.5

Testing the impact of morphological rate heterogeneity on ancestral state reconstruction of five floral traits in angiosperms

Supplementary Tables 6: Number of parsimony steps for each character with break-down between origins and reversals by partition

Elisabeth Reyes, Sophie Nadot, Maria von Balthazar, Jürg Schönenberger, Hervé Sauquet

Supplementary Tables 6: Number of parsimony steps for each character with break-down between origins and reversals by partition

Five-char: Results for main tree

PrunedMB: Results for the pruned Smith & Brown ALLMB tree

Symmetry

Min origins= Minimum number of origins; Max origins= Maximum number of origins; Min reversals= Minimum number of reversals; Max reversals = Maximum number of reversals

RESCORED DATA (Five-char)	Step number	Min origins	Max origins	Min reversals	Max reversals
Angiosperms	**217**	**148**	**182**	**35**	**69**
ANA+Magnoliidae	3	3	3	0	0
Monocotyledoneae	40	25	35	5	15
Basal eudicots	23	17	20	3	6
Superrosidae	63	44	53	10	19
Superasteridae	88	59	71	17	29
Outside partitions	0	0	0	0	0

ORIGINAL DATA (Five-char)	Step number	Min origins	Max origins	Min reversals	Max reversals
Angiosperms	**191**	**134**	**160**	**31**	**57**
ANA+Magnoliidae	3	3	3	0	0
Monocotyledoneae	38	27	33	5	11
Basal eudicots	20	16	17	3	4
Superrosidae	55	35	45	10	20
Superasteridae	75	53	62	13	22
Outside partitions	0	0	0	0	0

RESCORED DATA (PrunedMB)	Step number	Min origins	Max origins	Min reversals	Max reversals
Angiosperms	**207**	**130**	**173**	**34**	**77**
ANA+Magnoliidae	3	3	3	0	0
Monocotyledoneae	42	24	38	4	18
Basal eudicots	22	17	19	3	5
Superrosidae	59	32	51	8	27
Superasteridae	81	54	62	19	27
Outside partitions	0	0	0	0	0

Fusion

Min origins= Minimum number of origins; Max origins= Maximum number of origins; Min reversals= Minimum number of reversals; Max reversals = Maximum number of reversals

RESCORED DATA (Five-char)	Step number	Min origins	Max origins	Min reversals	Max reversals
**Angiosperms**	**212**	**71**	**130**	**82**	**141**
**ANA+Magnoliidae**	19	12	18	1	7
**Monocotyledoneae**	39	19	29	10	20
**Basal eudicots**	16	10	13	3	6
**Superrosidae**	76	21	42	34	55
**Superasteridae**	62	9	28	34	53
**Outside partitions**	0	0	0	0	0

ORIGINAL DATA (Five-char)	Step number	Min origins	Max origins	Min reversals	Max reversals
**Angiosperms**	**154**	**81**	**119**	**35**	**73**
**ANA+Magnoliidae**	13	13	13	0	0
**Monocotyledoneae**	34	21	26	8	13
**Basal eudicots**	6	5	5	1	1
**Superrosidae**	54	32	49	5	22
**Superasteridae**	47	10	26	21	37
**Outside partitions**	0	0	0	0	0

RESCORED DATA (PrunedMB)	Step number	Min origins	Max origins	Min reversals	Max reversals
**Angiosperms**	**212**	**70**	**135**	**77**	**142**
**ANA+Magnoliidae**	17	10	14	3	7
**Monocotyledoneae**	39	22	29	10	17
**Basal eudicots**	16	10	15	1	6
**Superrosidae**	82	19	57	25	62
**Superasteridae**	58	8	20	38	50
**Outside partitions**	0	1	0	0	0

Phyllotaxis

Min origins= Minimum number of origins; Max origins= Maximum number of origins; Min reversals= Minimum number of reversals; Max reversals = Maximum number of reversals

RESCORED DATA (Five-char)	Step number	Min origins	Max origins	Min reversals	Max reversals

Angiosperms	**31**	**23**	**28**	**3**	**8**
ANA+Magnoliidae	11	9	11	0	2
Monocotyledoneae	0	0	0	0	0
Basal eudicots	12	6	9	3	6
Superrosidae	4	4	4	0	0
Superasteridae	4	4	4	0	0
Outside partitions	0	0	0	0	0

ORIGINAL DATA (Five-char)	Step number	Min origins	Max origins	Min reversals	Max reversals
Angiosperms	**29**	**21**	**29**	**0**	**8**
ANA+Magnoliidae	11	9	11	0	2
Monocotyledoneae	0	0	0	0	0
Basal eudicots	11	5	11	0	6
Superrosidae	3	3	3	0	0
Superasteridae	4	4	4	0	0
Outside partitions	0	0	0	0	0

RESCORED DATA (PrunedMB)	Step number	Min origins	Max origins	Min reversals	Max reversals
Angiosperms	**28**	**21**	**27**	**1**	**7**
ANA+Magnoliidae	10	8	10	0	2
Monocotyledoneae	0	0	0	0	0
Basal eudicots	10	5	9	1	5
Superrosidae	4	4	4	0	0
Superasteridae	4	4	4	0	0
Outside partitions	0	0	0	0	0

Merism

Min origins= Minimum number of origins; Max origins= Maximum number of origins; Min reversals= Minimum number of reversals; Max reversals = Maximum number of reversals

RESCORED DATA (Five-char)	Step number	Min origins	Max origins	Min reversals	Max reversals
Angiosperms	**226**	**25**	**59**	**167**	**201**
ANA+Magnoliidae	6	5	6	0	1
Monocotyledoneae	1	1	1	0	0

**Basal eudicots**	9	3	5	4	6
**Superrosidae**	101	7	27	74	94
**Superasteridae**	108	8	19	89	100
**Outside partitions**	1	1	1	0	0

**ORIGINAL DATA (Five-char)**	**Step number**	**Min origins**	**Max origins**	**Min reversals**	**Max reversals**
**Angiosperms**	**199**	**33**	**96**	**103**	**166**
**ANA+Magnoliidae**	3	3	3	0	0
**Monocotyledoneae**	1	1	1	0	0
**Basal eudicots**	7	2	3	4	5
**Superrosidae**	88	17	47	41	71
**Superasteridae**	99	9	41	58	90
**Outside partitions**	1	1	1	0	0

**RESCORED DATA (PrunedMB)**	**Step number**	**Min origins**	**Max origins**	**Min reversals**	**Max reversals**
**Angiosperms**	**215**	**25**	**67**	**148**	**190**
**ANA+Magnoliidae**	6	5	6	0	1
**Monocotyledoneae**	1	1	1	0	0
**Basal eudicots**	9	2	5	4	7
**Superrosidae**	98	8	31	67	90
**Superasteridae**	100	8	23	77	92
**Outside partitions**	1	1	1	0	0

Differentiation

Min origins= Minimum number of origins; Max origins= Maximum number of origins; Min reversals= Minimum number of reversals; Max reversals = Maximum number of reversals

**RESCORED DATA (Five-char)**	**Step number**	**Min origins**	**Max origins**	**Min reversals**	**Max reversals**
**Angiosperms**	**161**	**44**	**76**	**85**	**117**
**ANA+Magnoliidae**	20	14	20	0	6
**Monocotyledoneae**	42	20	30	12	22
**Basal eudicots**	15	0	5	10	15
**Superrosidae**	51	3	8	43	48
**Superasteridae**	32	6	12	20	26

**Outside partitions**	1	1	1	0	0

**ORIGINAL DATA (Five-char)**	**Step number**	**Min origins**	**Max origins**	**Min reversals**	**Max reversals**
**Angiosperms**	**159**	**44**	**76**	**83**	**115**
**ANA+Magnoliidae**	19	13	19	0	6
**Monocotyledoneae**	42	20	30	12	22
**Basal eudicots**	16	1	6	10	15
**Superrosidae**	50	4	9	41	46
**Superasteridae**	32	6	12	20	26
**Outside partitions**	0	0	0	0	0

**RESCORED DATA (PrunedMB)**	**Step number**	**Min origins**	**Max origins**	**Min reversals**	**Max reversals**
**Angiosperms**	**151**	**33**	**66**	**85**	**118**
**ANA+Magnoliidae**	18	12	16	2	6
**Monocotyledoneae**	40	18	25	15	22
**Basal eudicots**	16	1	6	10	15
**Superrosidae**	50	1	9	41	49
**Superasteridae**	27	1	10	17	26
**Outside partitions**	0	0	0	0	0

Testing the impact of morphological rate heterogeneity on ancestral state reconstruction of five floral traits in angiosperms

Supplementary Figure S1: Original phylogeny showing the tip states for each species and the state reconstructed by parsimony for each tested node

Elisabeth Reyes, Sophie Nadot, Maria von Balthazar, Jürg Schönenberger, Hervé Sauquet

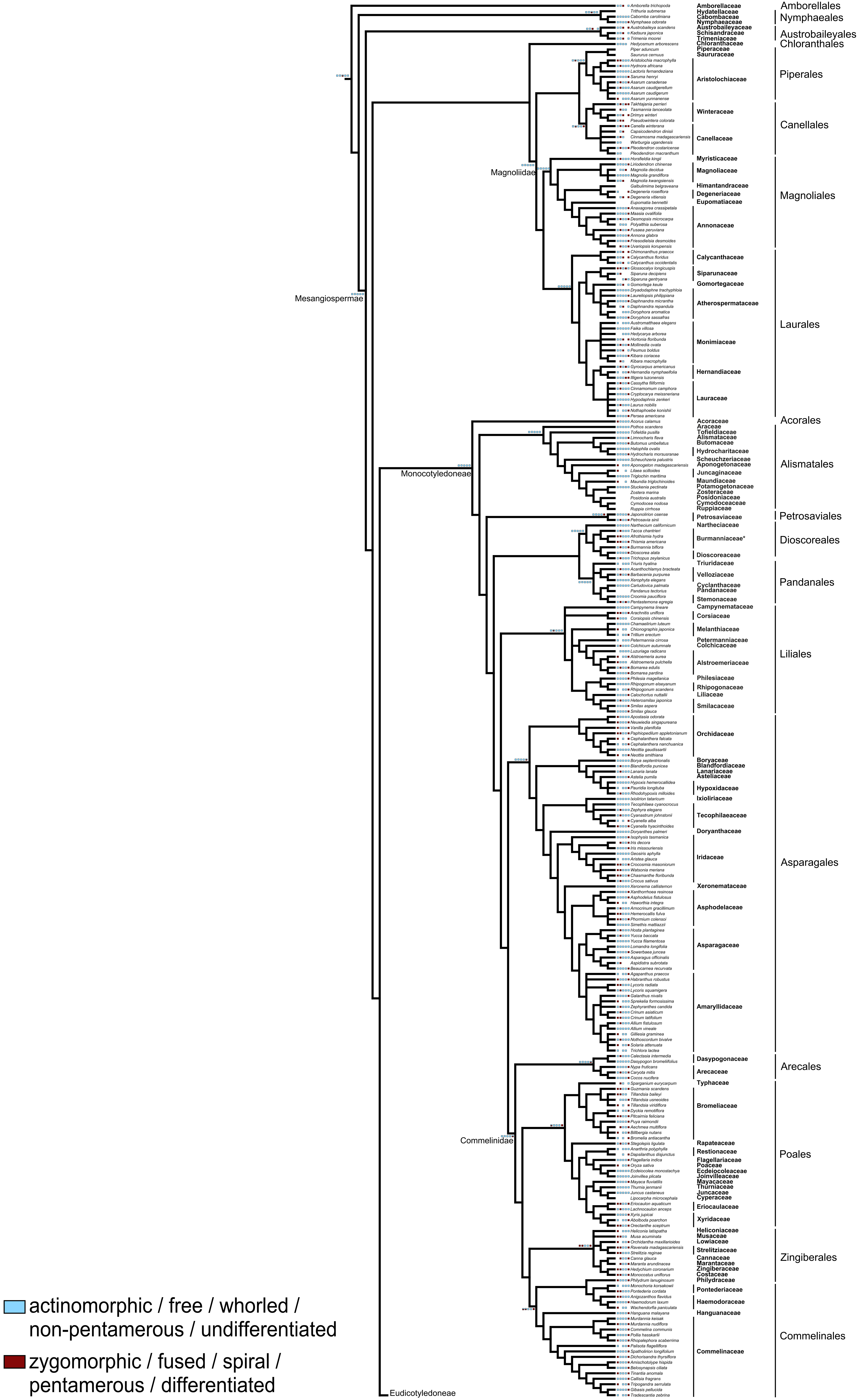

Amborellales
Nymphaeales
Austrobaileyales
Chloranthales
Piperales
Canellales
Magnoliales
Laurales
Acorales
Alismatales
Petrosaviales
Dioscoreales
Pandanales
Liliales
Asparagales
Arecales
Poales
Zingiberales
Commelinales
Amborellaceae
Hydatellaceae
Cabombaceae
Nymphaeaceae
Austrobaileyaceae
Schisandraceae
Trimeniaceae
Chloranthaceae
Piperaceae
Saururaceae
Aristolochiaceae
Winteraceae
Canellaceae
Myristicaceae
Magnoliaceae
Himantandraceae
Degeneriaceae
Eupomatiaceae
Annonaceae
Calycanthaceae
Siparunaceae
Gomortegaceae
Atherospermataceae
Monimiaceae
Hernandiaceae
Lauraceae
Acoraceae
Araceae
Tofieldiaceae
Alismataceae
Butomaceae
Hydrocharitaceae
Scheuchzeriaceae
Aponogetonaceae
Juncaginaceae
Maundiaceae
Potamogetonaceae
Zosteraceae
Posidoniaceae
Cymodoceaceae
Ruppiaceae
Petrosaviaceae
Nartheciaceae
Burmanniaceae*
Dioscoreaceae
Triuridaceae
Velloziaceae
Cyclanthaceae
Pandanaceae
Stemonaceae
Campynemataceae
Corsiaceae
Melanthiaceae
Petermanniaceae
Colchicaceae
Alstroemeriaceae
Philesiaceae
Rhipogonaceae
Liliaceae
Smilacaceae
Orchidaceae
Boryaceae
Blandfordiaceae
Lanariaceae
Asteliaceae
Hypoxidaceae
Ixioliriaceae
Tecophilaeaceae
Doryanthaceae
Iridaceae
Xeronemataceae
Asphodelaceae
Asparagaceae
Amaryllidaceae
Dasypogonaceae
Arecaceae
Typhaceae
Bromeliaceae
Rapateaceae
Restionaceae
Flagellariaceae
Poaceae
Ecdeiocoleaceae
Joinvilleaceae
Mayacaceae
Thurniaceae
Juncaceae
Cyperaceae
Eriocaulaceae
Xyridaceae
Heliconiaceae
Musaceae
Lowiaceae
Strelitziaceae
Cannaceae
Marantaceae
Zingiberaceae
Costaceae
Philydraceae
Pontederiaceae
Haemodoraceae
Hanguanaceae
Commelinaceae
Mesangiospermae
Magnoliidae
Monocotyledoneae
Commelinidae
Eudicotyledoneae
actinomorphic / free / whorled / non-pentamerous / undifferentiated
zygomorphic / fused / spiral / pentamerous / differentiated

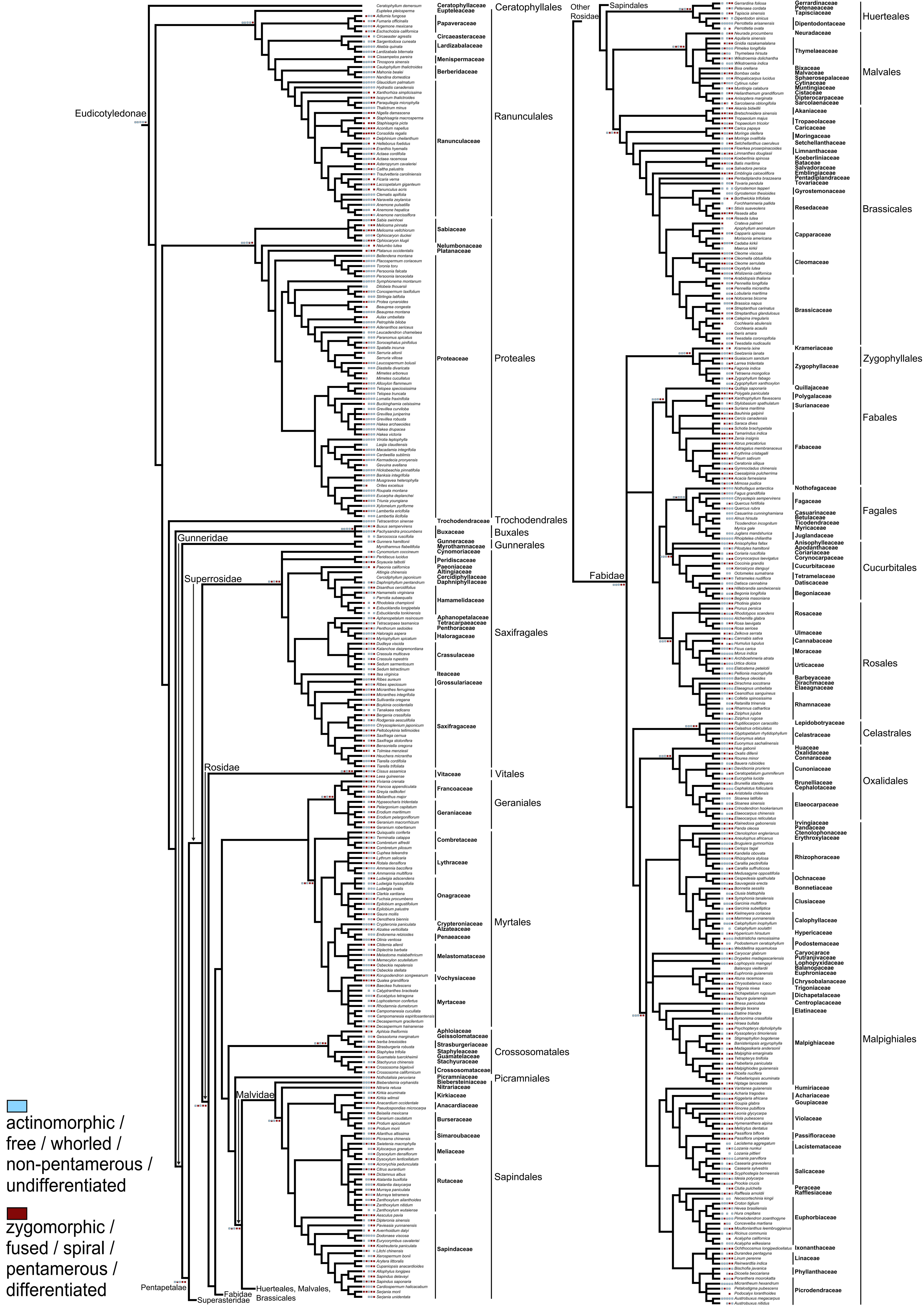

Eudicotyledonae
Ceratophyllales
Ranunculales
Proteales
Trochodendrales
Buxales
Gunnerales
Saxifragales
Vitales
Geraniales
Myrtales
Crossosomatales
Picramniales
Sapindales
Gunneridae
Superrosidae
Rosidae
Malvidae
Pentapetalae
Superasteridae
Fabidae
Huerteales, Malvales, Brassicales
Other Rosidae
Huerteales
Malvales
Brassicales
Zygophyllales
Fabales
Fagales
Cucurbitales
Rosales
Celastrales
Oxalidales
Malpighiales
Ceratophyllaceae
Eupteleaceae
Papaveraceae
Circaeasteraceae
Lardizabalaceae
Menispermaceae
Berberidaceae
Ranunculaceae
Sabiaceae
Nelumbonaceae
Platanaceae
Proteaceae
Trochodendraceae
Buxaceae
Gunneraceae
Myrothamnaceae
Cynomoriaceae
Peridiscaceae
Paeoniaceae
Altingiaceae
Cercidiphyllaceae
Daphniphyllaceae
Hamamelidaceae
Aphanopetalaceae
Tetracarpaeaceae
Penthoraceae
Haloragaceae
Crassulaceae
Iteaceae
Grossulariaceae
Saxifragaceae
Vitaceae
Francoaceae
Geraniaceae
Combretaceae
Lythraceae
Onagraceae
Crypteroniaceae
Alzateaceae
Penaeaceae
Melastomataceae
Vochysiaceae
Myrtaceae
Aphloiaceae
Geissolomataceae
Strasburgeriaceae
Staphyleaceae
Guamatelaceae
Stachyuraceae
Crossosomataceae
Picramniaceae
Biebersteiniaceae
Nitrariaceae
Kirkiaceae
Anacardiaceae
Burseraceae
Simaroubaceae
Meliaceae
Rutaceae
Sapindaceae
Gerrardinaceae
Petenaeaceae
Tapisciaceae
Dipentodontaceae
Neuradaceae
Thymelaeaceae
Bixaceae
Malvaceae
Sphaerosepalaceae
Cytinaceae
Muntingiaceae
Cistaceae
Dipterocarpaceae
Sarcolaenaceae
Akaniaceae
Tropaeolaceae
Caricaceae
Moringaceae
Setchellanthaceae
Limnanthaceae
Koeberliniaceae
Bataceae
Salvadoraceae
Emblingiaceae
Pentadiplandraceae
Tovariaceae
Gyrostemonaceae
Resedaceae
Capparaceae
Cleomaceae
Brassicaceae
Krameriaceae
Zygophyllaceae
Quillajaceae
Polygalaceae
Surianaceae
Fabaceae
Nothofagaceae
Fagaceae
Casuarinaceae
Betulaceae
Ticodendraceae
Myricaceae
Juglandaceae
Anisophylleaceae
Apodanthaceae
Coriariaceae
Corynocarpaceae
Cucurbitaceae
Tetramelaceae
Datiscaceae
Begoniaceae
Rosaceae
Ulmaceae
Cannabaceae
Moraceae
Urticaceae
Barbeyaceae
Dirachmaceae
Elaeagnaceae
Rhamnaceae
Lepidobotryaceae
Celastraceae
Huaceae
Oxalidaceae
Connaraceae
Cunoniaceae
Brunelliaceae
Cephalotaceae
Elaeocarpaceae
Irvingiaceae
Pandaceae
Ctenolophonaceae
Erythroxylaceae
Rhizophoraceae
Ochnaceae
Bonnetiaceae
Clusiaceae
Calophyllaceae
Hypericaceae
Podostemaceae
Caryocaraceae
Putranjivaceae
Lophopyxidaceae
Balanopaceae
Euphroniaceae
Chrysobalanaceae
Trigoniaceae
Dichapetalaceae
Centroplacaceae
Elatinaceae
Malpighiaceae
Humiriaceae
Achariaceae
Goupiaceae
Violaceae
Passifloraceae
Lacistemataceae
Salicaceae
Peraceae
Rafflesiaceae
Euphorbiaceae
Ixonanthaceae
Linaceae
Phyllanthaceae
Picrodendraceae
actinomorphic / free / whorled / non-pentamerous / undifferentiated
zygomorphic / fused / spiral / pentamerous / differentiated

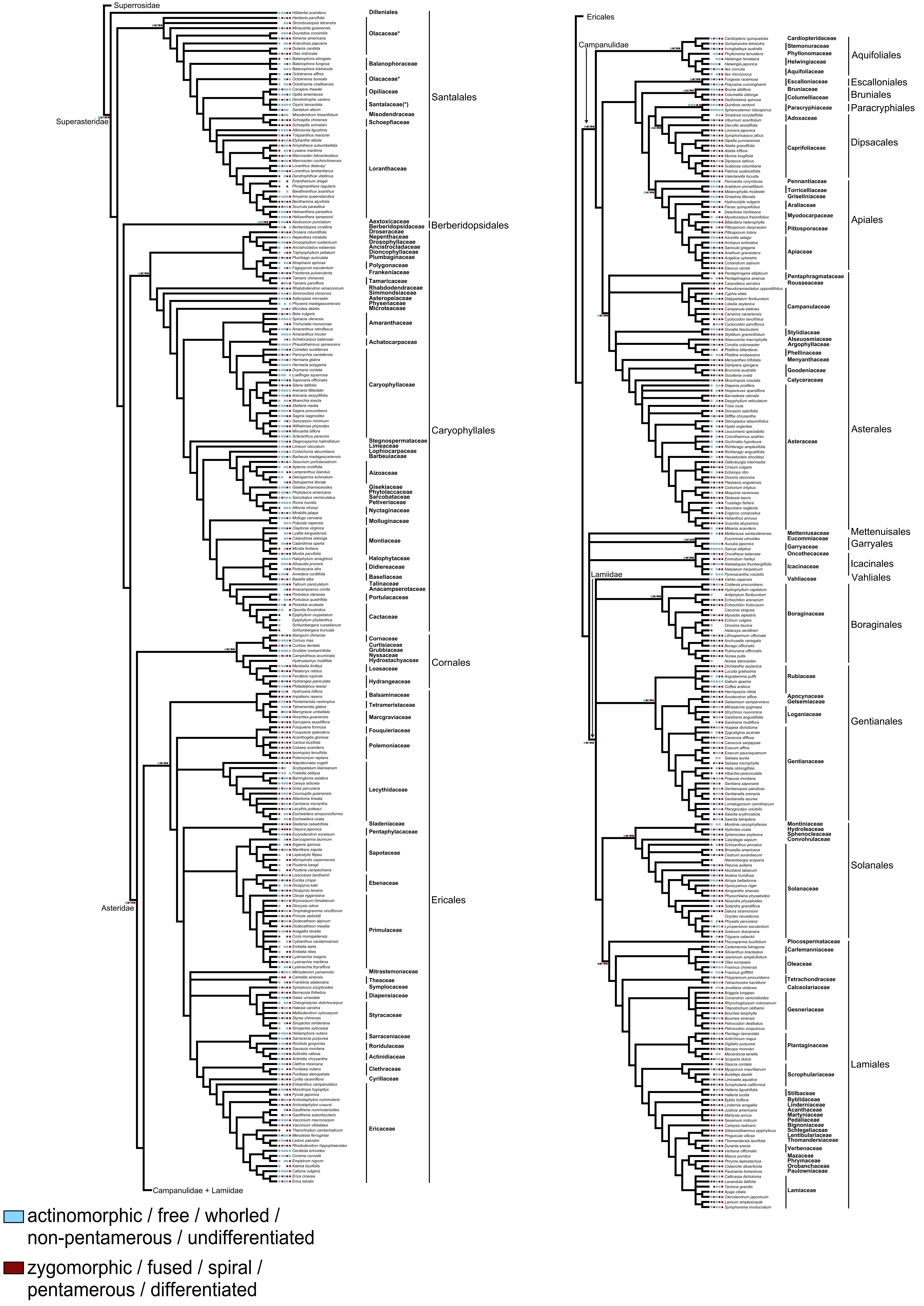

Superrosidae
Superasteridae
Asteridae
Campanulidae + Lamiidae
Ericales
Campanulidae
Lamiidae
Santalales
Berberidopsidales
Caryophyllales
Cornales
Ericales
Aquifoliales
Escalloniales
Bruniales
Paracryphiales
Dipsacales
Apiales
Asterales
Metteniusales
Garryales
Icacinales
Vahliales
Boraginales
Gentianales
Solanales
Lamiales
actinomorphic / free / whorled / non-pentamerous / undifferentiated
zygomorphic / fused / spiral / pentamerous / differentiated

Testing the impact of morphological rate heterogeneity on ancestral state reconstruction of five floral traits in angiosperms

Supplementary Figure S2: Node ages in the original tree

Elisabeth Reyes, Sophie Nadot, Maria von Balthazar, Jürg Schönenberger, Hervé Sauquet

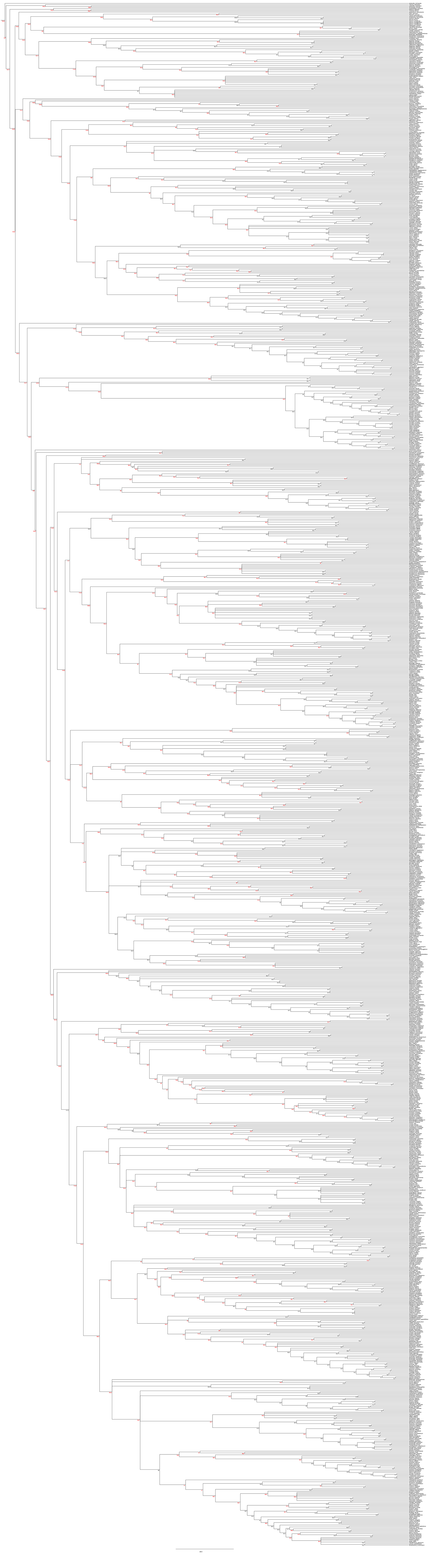

Testing the impact of morphological rate heterogeneity on ancestral state reconstruction of five floral traits in angiosperms

Supplementary Data 1: Nexus file for the original tree

Elisabeth Reyes, Sophie Nadot, Maria von Balthazar, Jürg Schönenberger, Hervé Sauquet

Testing the impact of morphological rate heterogeneity on ancestral state reconstruction of five floral traits in angiosperms

Supplementary Data 2: PROTEUS data used to produce the original Nexus file

Elisabeth Reyes, Sophie Nadot, Maria von Balthazar, Jürg Schönenberger, Hervé Sauquet

Testing the impact of morphological rate heterogeneity on ancestral state reconstruction of five floral traits in angiosperms

Supplementary Data 3: Nexus file for the pruned Smith & Brown tree

Elisabeth Reyes, Sophie Nadot, Maria von Balthazar, Jürg Schönenberger, Hervé Sauquet
